# Biomimetic Nanoplatform for Dual Target Nano‐Metabolic Therapy in Diabetes‐Associated Biofilm Infections

**DOI:** 10.1002/advs.76411

**Published:** 2026-07-03

**Authors:** Mingzhang Li, Yonglong Li, Jiren Yan, Changming Wang, Jinlong Yu, Feng Jiang, Boyong Wang, Yi Yang, Deyi Ding, Jin Tang, Pei Han, Botao Song, Geyong Guo, Hao Shen

**Affiliations:** ^1^ Department of Orthopedics Shanghai Sixth People's Hospital Affiliated to Shanghai Jiao Tong University School of Medicine Shanghai Jiao Tong University Shanghai P. R. China; ^2^ Key Laboratory of Synthetic and Natural Functional Molecule of the Ministry of Education, College of Chemistry and Materials Science Northwest University Xi'an P. R. China; ^3^ Department of Clinical Laboratory, Shanghai Sixth People's Hospital Affiliated to Shanghai Jiao Tong University School of Medicine Shanghai Jiao Tong University Shanghai P. R. China

**Keywords:** biofilm, diabetes‐associated infection, macrophage, nano‐metabolic therapy

## Abstract

Metabolism alterations significantly influence the behavior of both bacteria and immune cells in the microenvironment of diabetes‐associated biofilm infections, ultimately determining the outcome of infections. Here, we propose a dual‐target nano‐metabolic therapy based on a biomimetic nanoplatform to combat these infections. The nanoplatform, coated with cellular membranes from pre‐infected macrophages and loaded with glucose oxidase (GOx) and L‐arginine (Arg), facilitates targeted drug delivery. Nitric oxide (NO), generated in situ through the catalytic cascade reaction of GOx and Arg, acts as a dual‐target metabolic regulator. It disrupts bacterial carbon and nitrogen metabolism, particularly the tricarboxylic acid (TCA) cycle and amino acid metabolism, effectively eliminating biofilms. Simultaneously, NO modulates macrophage metabolism, shifting it from oxidative phosphorylation to aerobic glycolysis by suppressing TCA cycle enzymes and electron transport chain complexes, while preserving mitochondrial integrity. This energy metabolism transition reverses macrophage immunosuppression, enhancing their phagocytic and invasive functions to promote infection clearance. Experiments with multiple clinical bacterial strains and various diabetic infection models highlight the therapeutic potential of combining metabolism interference with immune modulation, offering new insights for the treatment of diabetes‐associated biofilm infections.

## Graphical abstract

A biomimetic GOx/Arg‐loaded nanoplatform produces nitric oxide through an in situ catalytic cascade reaction, enabling dual‐target metabolic regulation in diabetes‐associated biofilm infections. Bacterial metabolism is disrupted, and macrophage immunometabolism is reprogrammed, resulting in biofilm eradication, immune restoration, and enhanced infection clearance.

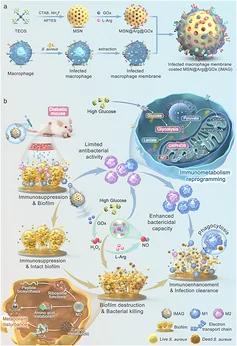

## 1 Introduction

Diabetes‐associated infections, including wound and implant‐associated infections, pose serious risks such as surgical failure, sepsis, and death, largely due to the challenges of biofilm formation exacerbated by high glucose and hypoxia at infection sites [1, 2, 3]. The dense structure of biofilms hinders the penetration of antimicrobials and invasion of macrophages, while the virulence factors secreted by bacteria suppress macrophage immune function through various pathways, leading to anti‐inflammatory polarization and reduced bactericidal activities [4]. Hyperglycemia‐induced immune disorders further enhance the biofilm's resistance and promote immune suppression [5, 6]. Due to the unique characteristics of diabetes‐associated biofilm infections, the efficacy of various treatments remains limited [7]. When the treatment targets the biofilm exclusively, suppressed immune cells fail to manage the residual bacteria released from lysed biofilms, resulting in pathogen dissemination and re‐colonization [8, 9]. Additionally, strategies that focus solely on enhancing immune cell function can be inadequate, as the physical barrier of the biofilm limits the antimicrobial activity of these immune cells [10, 11]. Therefore, it is crucial to explore a novel therapeutic strategy that simultaneously disrupts the biofilm, eliminates bacteria, and restores macrophage function to improve treatment outcomes for diabetes‐associated biofilm infections [12].

Bacterial invasion, colonization, proliferation, and biofilm formation are fundamentally dependent on energy metabolism [13]. The tricarboxylic acid (TCA) cycle not only generates ATP for cellular activities but also plays a crucial role in regulating virulence proteins and promoting the synthesis of carbohydrates and proteins in the biofilm matrix [14, 15]. Furthermore, biofilms adeptly exploit metabolic pathways, such as carbohydrate and amino acid metabolism, to resist host immune defenses [16]. Meanwhile, cellular metabolism is also significant for immune function. Through glycolysis, activated macrophages generate intermediates necessary for the synthesis of pro‐inflammatory cytokines and produce NADPH to support respiratory bursts for bacterial killing [17]. However, in the context of biofilm infections, macrophages undergo metabolic reprogramming, with energy metabolism shifting toward the TCA cycle and oxidative phosphorylation (OXPHOS). This transition leads to a change from a pro‐inflammatory to an anti‐inflammatory phenotype, resulting in immune suppression [18]. Moreover, the high‐glucose microenvironment in diabetes provides metabolic substrates that stimulate biofilm formation [19], exacerbating immune dysfunction in macrophages. Thus, leveraging the abundant glucose in the diabetic environment to disrupt bacterial metabolism for antibacterial and anti‐biofilm effects, while simultaneously reshaping macrophage metabolism to restore immune function, may represent a promising therapeutic strategy for diabetes‐associated biofilm infections [12, 20].

Glucose oxidase (GOx) has garnered great attention in the diagnosis and treatment of diabetes‐associated infections due to its excellent biocompatibility and specific glucose‐catalyzing ability [21, 22]. Although GOx rapidly catalyzes glucose into large amounts of hydrogen peroxide (H_2_O_2_), exerting antibacterial effects, the resulting burst of oxidative stress raises concerns about tissue damage, and the inherent instability of H_2_O_2_ limits its therapeutic efficacy. The conversion of H_2_O_2_ to nitric oxide (NO) offers a promising alternative. As a gaseous molecule, NO can penetrate biofilm barriers and exert bactericidal effects internally. Additionally, its lower cytotoxicity makes NO an ideal metabolic regulator. L‐arginine (Arg), a natural amino acid, serves as a precursor for NO, which is catalytically generated from Arg in response to reactive oxygen species (ROS) [23, 24]. This ROS‐triggered NO production helps reduce excessive oxidative stress and drug toxicity, while appropriate Arg supplementation compensates for potential deficits in NO synthesis. Moreover, mesoporous silica nanoparticles (MSNs), known for their excellent biocompatibility, controllable pore sizes, and high specific surface area, emerge as ideal drug delivery nanoplatforms [25, 26]. However, precise drug delivery and effective accumulation at the infection site remain challenges to be addressed.

In recent years, biomimetic nanotechnology based on naturally derived cellular membrane‐camouflaged drug cores has been widely applied in drug delivery [27, 28, 29]. These cellular membrane‐derived drug delivery systems benefit from the protection of lipid bilayers and receptor‐ligand‐specific recognition, enabling drugs to evade biological clearance and effectively accumulate at the affected sites [30, 31]. Notably, decorating nanocarriers with cell membranes derived from bacteria‐infected macrophages not only enhances their stability in vivo but also allows the abundant pattern recognition receptors (PRRs) on the cell membrane surface to actively identify and bind to pathogens [32]. This improves the targeting capability of the nanocarriers, reduces off‐target effects, and enhances biosafety.

In this study, a biomimetic drug delivery nanoplatform, IMAG, was designed by combining cell membranes derived from *Staphylococcus aureus* (*S. aureus*)‐infected macrophages with Arg and GOx‐loaded MSNs, proposing a bacteria‐macrophage dual‐target nano‐metabolic therapy for diabetes‐associated biofilm infections (Figure 1a). In contrast to conventional GOx‐based nanoplatforms that primarily rely on oxidative stress‐mediated antibacterial effects, IMAG was designed to function as a metabolic regulation‐driven therapeutic system [33, 34, 35]. The cell membranes derived from infected macrophages guided nanoparticles to the infection site, facilitating bacterial recognition and adhesion. H_2_O_2_, generated through GOx‐catalyzed glucose oxidation, reacts with Arg to produce NO in situ. As a dual‐target metabolic regulator, NO reduces bacterial viability and interferes with biofilm formation by disrupting the bacterial TCA cycle and amino acid metabolism. Meanwhile, NO shifts macrophage metabolism from OXPHOS to aerobic glycolysis by inhibiting TCA cycle‐related enzymes and respiratory chain complexes, thereby restoring macrophage immune functions and enhancing its phagocytic and invasive activities. This dual‐target nano‐metabolic therapy, mediated by the metabolic regulator generated through the cascade catalysis of the biomimetic nanoplatform, ultimately accelerates infection clearance, offering a novel perspective for the treatment of diabetes‐associated biofilm infections (Figure 1b).

**FIGURE 1:**
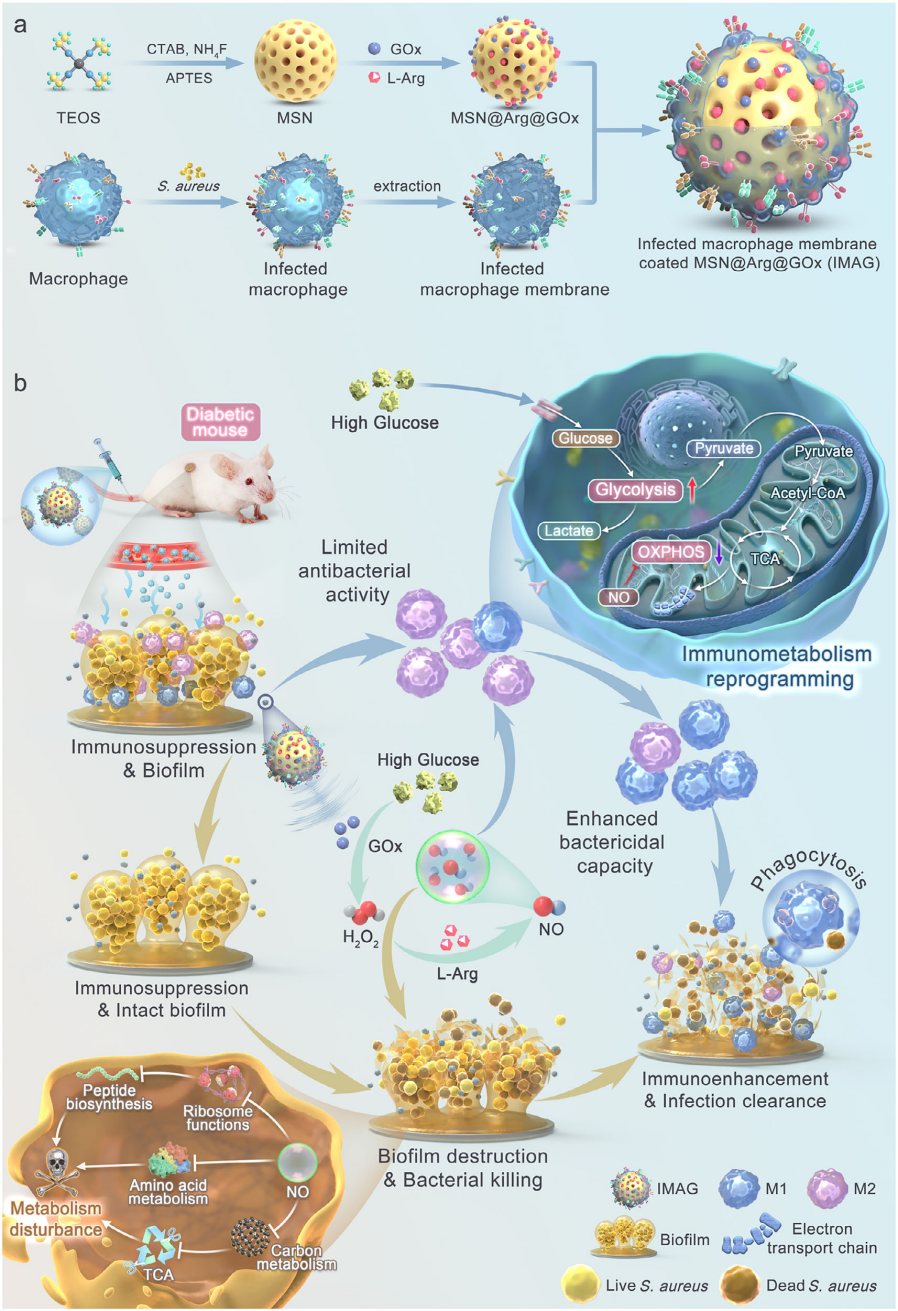
The biomimetic nanoplatform‐based dual‐target nano‐metabolic therapy for diabetes‐associated biofilm infections. (a) Schematic diagram of the synthesis of IMAG. (b) Schematic diagram for IMAG‐mediated bacteria‐macrophage dual‐target nano‐metabolic therapy for diabetes‐associated biofilm infections.

## 2 Results and Discussion

### 2.1 Synthesis and Characterization of IMAG

As reported by Song et al., pristine MSNs (pMSNs) were synthesized using the sol‐gel process [36], and the residual fluoride and bromide levels were confirmed to be negligible after calcination (Figure S1a). Compared with pMSNs reported previously, the MSNs prepared here were gently amino‐functionalized with a small amount of APTES to facilitate GOx immobilization while preserving surface charge stability and biological compatibility (Figure S1b–e). GOx and Arg were subsequently infiltrated into the MSNs using the optimized feeding ratios determined from encapsulation efficiency and loading content measurements, yielding MSN@Arg@GOx composite nanoparticles (Figure S2 and Figure 1a).

Scanning electron microscopy (SEM) and transmission electron microscopy (TEM) showed uniform particle sizes for both MSNs and MSN@Arg@GOx, with no significant morphological changes observed in MSN@Arg@GOx after drug loading (Figure 2a). Elemental mapping of MSN@Arg@GOx showed a homogeneous distribution of C, N, O, and Si, which is consistent with its composition (Figure 2b). In the FTIR spectra, the strong and broad absorption band around 1050 cm^−1^ is attributed to the Si─O─Si asymmetric stretching vibrations, which is a characteristic feature of the mesoporous silica framework (Figure 2c). Additionally, peaks at 799 cm^−1^ and 467 cm^−1^ correspond to the symmetric stretching and bending vibrations of the Si─O bond, respectively. For GOx, two characteristic protein absorption bands at approximately 1662 cm^−1^ and 1538 cm^−1^ were observed. The presence of Arg is confirmed by an absorption band observed at 1562 cm^−1^, along with corresponding peaks at 1558 cm^−1^ and 1556 cm^−1^, all of which are characteristic of the C═O group in Arg. In the spectrum of MSN@Arg@GOx, the distinct signals of GOx and Arg can be observed. Furthermore, UV–vis absorption spectra revealed that GOx exhibits significant absorbance at 200 and 280 nm (Figure 2d). Arg also shows UV absorbance at 200 nm. After loading these two drugs onto the MSNs, the characteristic absorption peaks of both drugs were observed in the UV spectra, appearing at 195 and 260 nm, respectively. The average hydrodynamic diameter of the MSNs is approximately 130 nm, with a surface zeta potential of −29.2 mV. After drug loading, the average hydrodynamic diameter of MSN@Arg@GOx increased to 150 nm, and the surface zeta potential rose to −22.7 mV (Figure 2e,f). The decrease in specific surface area after co‐loading further verified the successful encapsulation of both GOx and Arg (Figure S3). The release profiles and half‐lives of GOx and L‐Arg under different conditions further demonstrated controlled, environment‐responsive sustained release from the nanoparticles (Figure S4).

**FIGURE 2:**
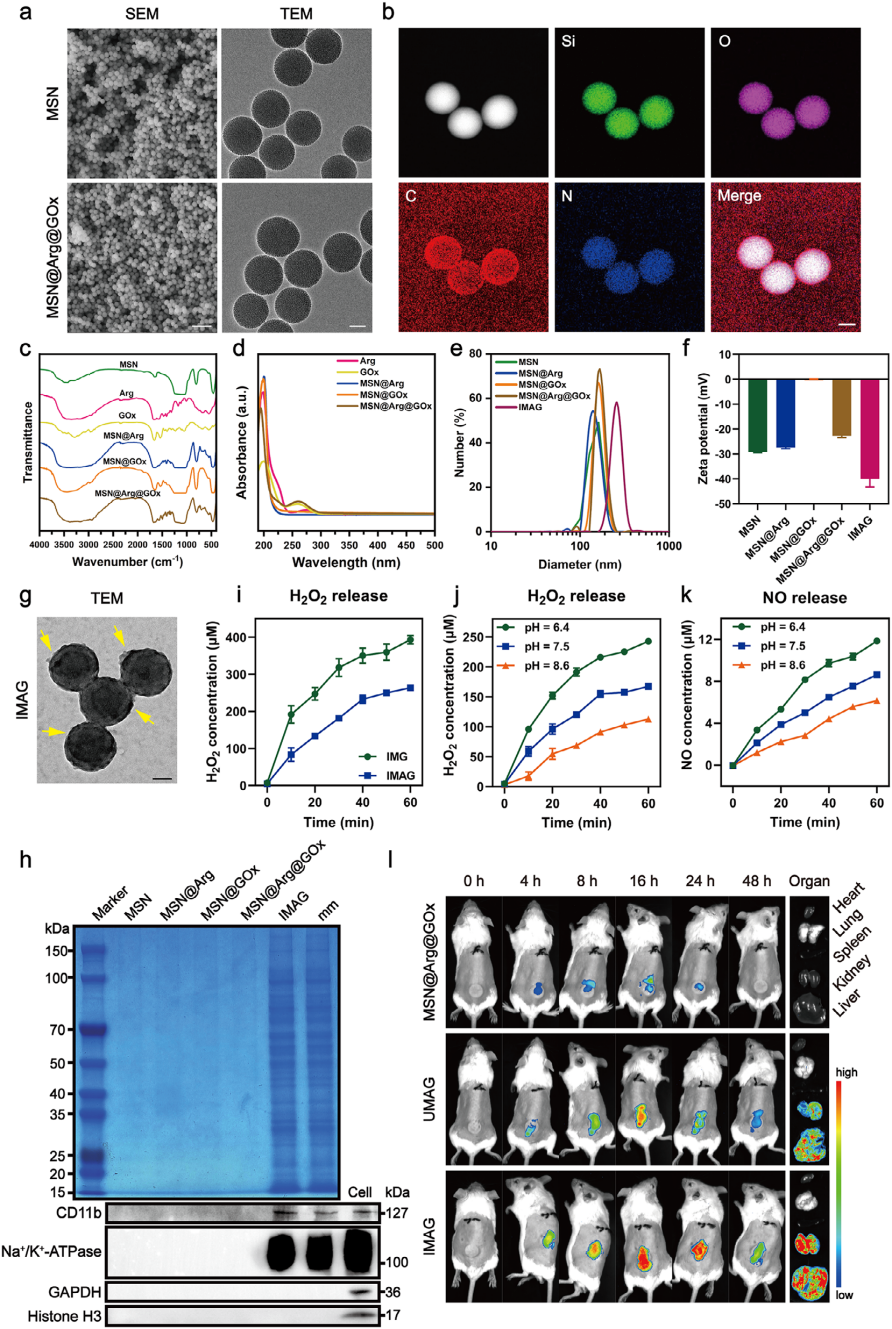
Characteristics of MSN@Arg@GOx and IMAG. (a) SEM and TEM images of MSN and MSN@Arg@GOx. Scale bars, 500 nm and 50 nm. (b) Elemental mapping images of MSN@Arg@GOx. Scale bar, 50 nm. (c) FTIR spectra of Arg, GOx, MSN@Arg, MSN@GOx, and MSN@Arg@GOx. (d) UV–vis absorption spectra of MSN, Arg, GOx, MSN@Arg, MSN@GOx, and MSN@Arg@GOx. (e) Hydrodynamic diameters of MSN, MSN@Arg, MSN@GOx, MSN@Arg@GOx, and IMAG. (f) Zeta potentials of MSN, MSN@Arg, MSN@GOx, MSN@Arg@GOx, and IMAG. (g) TEM images of IMAG, with yellow arrows pointing to the macrophage membranes coating the nanoparticles. Scale bar, 50 nm. (h) SDS‐PAGE gel images for detecting protein contents in different groups, along with western blot (WB) analysis of characteristic proteins (CD11b, Na^+^/K^+^‐ATPase, GAPDH, and Histone H3). (i) H_2_O_2_ generation capability of IMG and IMAG (*n* = 3). (j) H_2_O_2_ generation capability of IMAG under different pH conditions (*n* = 3). (k) NO generation capability of IMAG under different pH conditions (*n* = 3). (l) Fluorescence images of infected sites and major organs at 0, 4, 8, 16, 24, and 48 h postinjection.

After collecting the infected macrophages, the cell membranes were extracted to encapsulate MSN@Arg@GOx, forming IMAG. TEM results for IMAG revealed a distinct structure of cell membranes coating the nanoparticles (Figure 2g). SDS‐PAGE analysis further confirmed that the membrane protein composition remained largely consistent before and after encapsulation, indicating successful membrane retention and preservation of membrane surface protein functions (Figure 2h). Furthermore, western blot analysis demonstrated the presence of CD11b, a characteristic marker of macrophage membranes, validating the successful coating of membranes onto MSN@Arg@GOx nanoparticles. Compared to whole cells, IMAG exhibited only the membrane protein Na^+^/K^+^‐ATPase, but lacked nuclear protein Histone H3 and cytoplasmic protein GAPDH, further confirming the purity of the membrane coating. Compared to MSN@Arg@GOx, IMAG showed an increased hydrodynamic diameter and decreased zeta potential, reflecting the successful coverage of the cellular membrane (Figure 2e,f).

Subsequently, MSN, MSN@Arg, and MSN@GOx were similarly coated with membranes from pre‐infected macrophages to form IM, IMA, and IMG. To systematically benchmark the performance of IMAG, a set of well‐defined control nanoplatforms was established under identical experimental conditions. GOx‐based and NO‐donor‐based systems (IMG and IMA) were used as catalytic metabolic controls, while macrophage membrane‐coated systems with or without infection priming (UMAG and IMAG) were used to evaluate infection‐adaptive targeting capability. This design enabled direct comparison of the functional contributions of each module within the IMAG platform.

To ensure biosafety and determine the working concentration for subsequent experiments, a series of in vitro evaluations was performed. Nanoparticles were subjected to sterility assays, confirming sterility, with no detectable viable bacteria (Figure S5). The in vitro cytotoxicity assays showed that IMAG at 500 µg mL^−1^ did not substantially affect cell viability, whereas a higher dose (750 µg mL^−1^) significantly reduced viability in macrophages (RAW264.7) and fibroblasts (L929) (Figure S6). At the selected working concentration (500 µg mL^−1^), CCK8 and live/dead staining confirmed biocompatibility and no obvious cytoskeletal damage across all nanoparticle groups (Figure S7). Hemolysis assays also showed no significant red blood cell lysis (Figure S8). Therefore, a concentration of 500 µg mL^−1^ was used as the same experimental concentration for both antibacterial and cell‐related experiments.

To investigate the catalytic efficiency, IMG and IMAG were incubated with glucose solutions, and the concentration of H_2_O_2_ was measured over time. IMAG generated less H_2_O_2_ than IMG over the same period despite equal concentrations (Figure 2i), likely due to in situ generation of NO in response to H_2_O_2_. Further assays demonstrated that IMAG exhibited enhanced catalytic efficiency under mildly acidic conditions (Figure 2j,k), highlighting the potential application of IMAG in diabetic infections, where the microenvironment is characterized by high glucose and acidity.

The role of macrophage membrane coating in infection‐site targeting was further evaluated. Compared with non‐membrane‐coated nanoparticles (MSN@Arg@GOx) and uninfected macrophage membrane‐coated nanoparticles (UMAG), IMAG exhibited stronger adhesion to *S. aureus* and *Escherichia coli* (*E. coli*) (Figures S9 and S10), consistent with the increased expression of PRRs, including Toll‐like receptors (TLR2, TLR4, and TLR6) on infected macrophage membranes (Figure S11). In vivo, diabetic mice with implant infections were intravenously injected with Cy5.5‐labeled MSN@Arg@GOx, UMAG, or IMAG. Real‐time fluorescence imaging and plasma fluorescence analysis were used to monitor biodistribution and accumulation at the infection site (Figure 2l and Figure S12). IMAG showed the highest signal intensity, fastest accumulation, and longest retention at the infection site. At 48 h post‐injection, minimal fluorescence remained in the MSN@Arg@GOx group, whereas IMAG retained significantly higher signal intensity than UMAG, with corresponding accumulation also observed in the liver and kidneys. These results indicate that IMAG exhibits excellent infection‐site targeting ability and prolonged retention.

### 2.2 Antibacterial and Anti‐Biofilm Effects of IMAG In Vitro

The antibacterial effect of IMAG in vitro was evaluated in vitro (Figure 3a). Nanoparticles from different groups were co‐cultured with *S. aureus* or *E. coli*, using an equal volume of PBS as the control. After treatment with IMAG, bacterial proliferation was inhibited (Figure 3b and Figure S13), and the bacterial viability was significantly lower than that of the control group (Figure 3c and Figure S14). Live/dead staining images and flow cytometry revealed consistent results, with a significantly higher proportion of dead bacteria (red fluorescence) in the IMAG‐treated group compared to others (Figure 3d,e and Figures S15 and S16). While IMG exhibited some antibacterial activity, its effect was significantly weaker than that of IMAG. Combined with the results from H_2_O_2_ and NO release assays (Figure 2i–k), these findings suggest that IMAG's antibacterial effect is primarily attributed to NO rather than H_2_O_2_. Overall, these results demonstrate the strong antibacterial activity of IMAG against planktonic *S. aureus* and *E. coli*.

**FIGURE 3:**
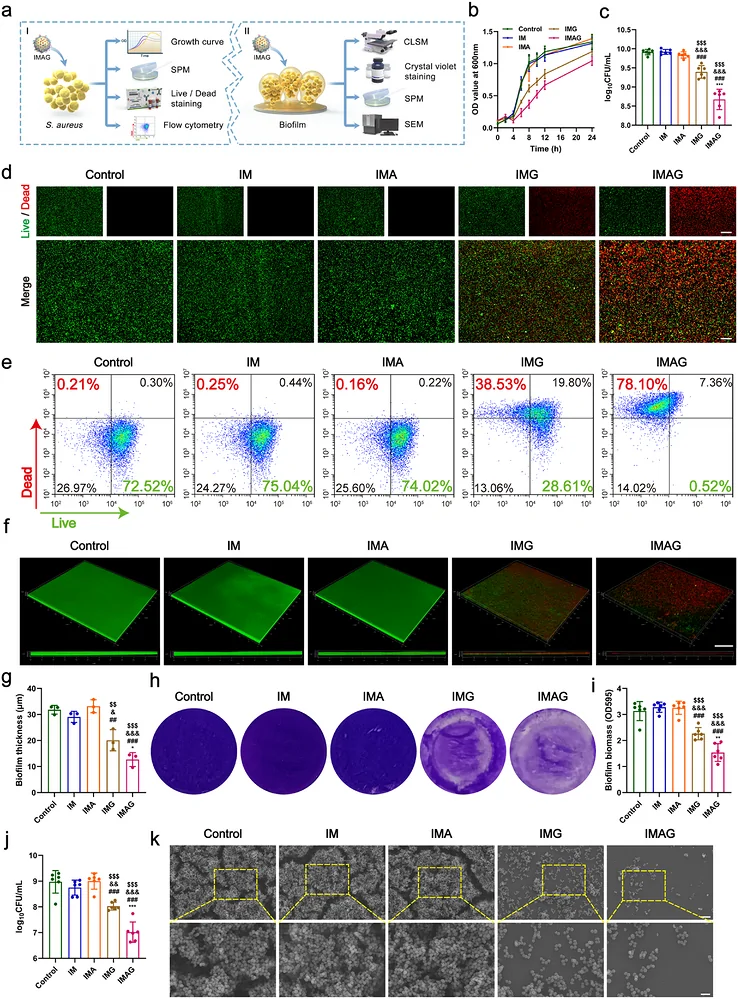
Antibacterial and antibiofilm effects of IMAG in vitro. (a) Schematic diagram of the in vitro antibacterial experiment. (b) Growth curves of *S. aureus* in each group (*n* = 6). (c) CFU count of *S. aureus* (*n* = 6). (d) Live/dead staining of *S. aureus*, with live bacteria emitting green fluorescence and dead bacteria emitting red fluorescence. Scale bars, 200 µm and 100 µm. (e) Representative live/dead flow cytometry results for *S. aureus* in each group. (f) 3D reconstruction of *S. aureus* biofilm using CLSM. Green represents live bacteria, while red represents dead bacteria. Scale bar, 200 µm. (g) Thickness of *S. aureus* biofilms (*n* = 3). (h) Representative images of crystal violet‐stained *S. aureus* biofilms. (i) Absorbance at OD595 after dissolving the crystal violet‐stained biofilm to assess biofilm biomass (*n* = 6). (j) Bacterial load in different groups of biofilms (*n* = 6). (k) SEM images of the *S. aureus* biofilms in each treatment group. Scale bars, 5 µm and 2 µm. Note: ^$$^ *p* < 0.01 and ^$$$^ *p* < 0.001 vs. the control group; ^&^ *p *< 0.05, ^&&^ *p *< 0.01 and ^&&&^ *p *< 0.001 vs. IM group; ^##^ *p *< 0.01 and ^###^ *p *< 0.001 vs. IMA group; ^*^ *p *< 0.05, ^**^ *p *< 0.01 and ^***^ *p *< 0.001 versus IMG group. Data are presented as mean ± SD. Statistical significance was determined by one‐way ANOVA.

Unlike planktonic bacteria, biofilms contain proteins, polysaccharides, and DNA, forming a polymeric barrier that impedes antibiotic penetration and immune cell clearance, thereby contributing to the chronicity of orthopedic implant and wound infections [37, 38, 39]. We further evaluated the effect of IMAG against biofilms. Confocal laser scanning microscopy (CLSM) revealed that IMAG‐treated biofilms were sparse and loose, with significantly reduced bacterial viability and biofilm thickness compared to the control group, which exhibited high bacterial activity and dense biofilm structure (Figure 3f,g and Figure S17). Consistent with these findings, crystal violet staining revealed reduced biofilm biomass following IMAG treatment, with the IMAG group showing the lowest bacterial load, suggesting effective eradication of bacteria within the biofilm (Figure 3h–j and Figures S18 and S19). Furthermore, SEM analysis showed fewer bacterial colonies in the IMAG group, whereas the control group exhibited substantial clustering of *S. aureus* and *E. coli* (Figure 3k and Figure S20). Collectively, these results indicate the high efficacy of IMAG in biofilm clearance.

Importantly, these in vitro experiments were performed in glucose‐containing bacterial culture media, including TSB (2.5 g/L glucose) and TSBG (TSB supplemented with 0.25% glucose), which provide sufficient substrate to support GOx‐mediated catalytic activity and facilitate NO generation, thereby mimicking a glucose‐replete microenvironment relevant to diabetic infection conditions in vivo.

### 2.3 Mechanisms Underlying the Antibacterial and Anti‐Biofilm Effects of IMAG In Vitro

To investigate the antibacterial and anti‐biofilm mechanisms of IMAG, RNA sequencing (RNA‐seq) was performed on *S. aureus* co‐cultured with IMAG, with PBS treatment as a control (Figure 4a). The volcano plot revealed that 179 genes were upregulated and 168 genes were downregulated following IMAG treatment compared to the control (Figure 4b). The heatmap of differentially expressed genes (DEGs) indicated significant heterogeneity between groups and similarity within the groups (Figure S21). Kyoto Encyclopedia of Genes and Genomes (KEGG) and Gene Ontology (GO) analyses showed that IMAG treatment notably disrupted the carbon and nitrogen metabolism of *S. aureus*, as evidenced by the downregulation of pathways related to ABC transporters, the TCA cycle, amino acid metabolism (cysteine and methionine metabolism, glycine, serine, and threonine metabolism), and ribosome function (Figure 4c,d). Further analysis of the heatmap for DEGs in these pathways indicated a significant decrease in the expression of genes encoding ABC transport proteins, enzymes involved in amino acid metabolism and the TCA cycle, as well as various ribosomal protein subunits (Figure 4e,f, and Figure S22a).

**FIGURE 4:**
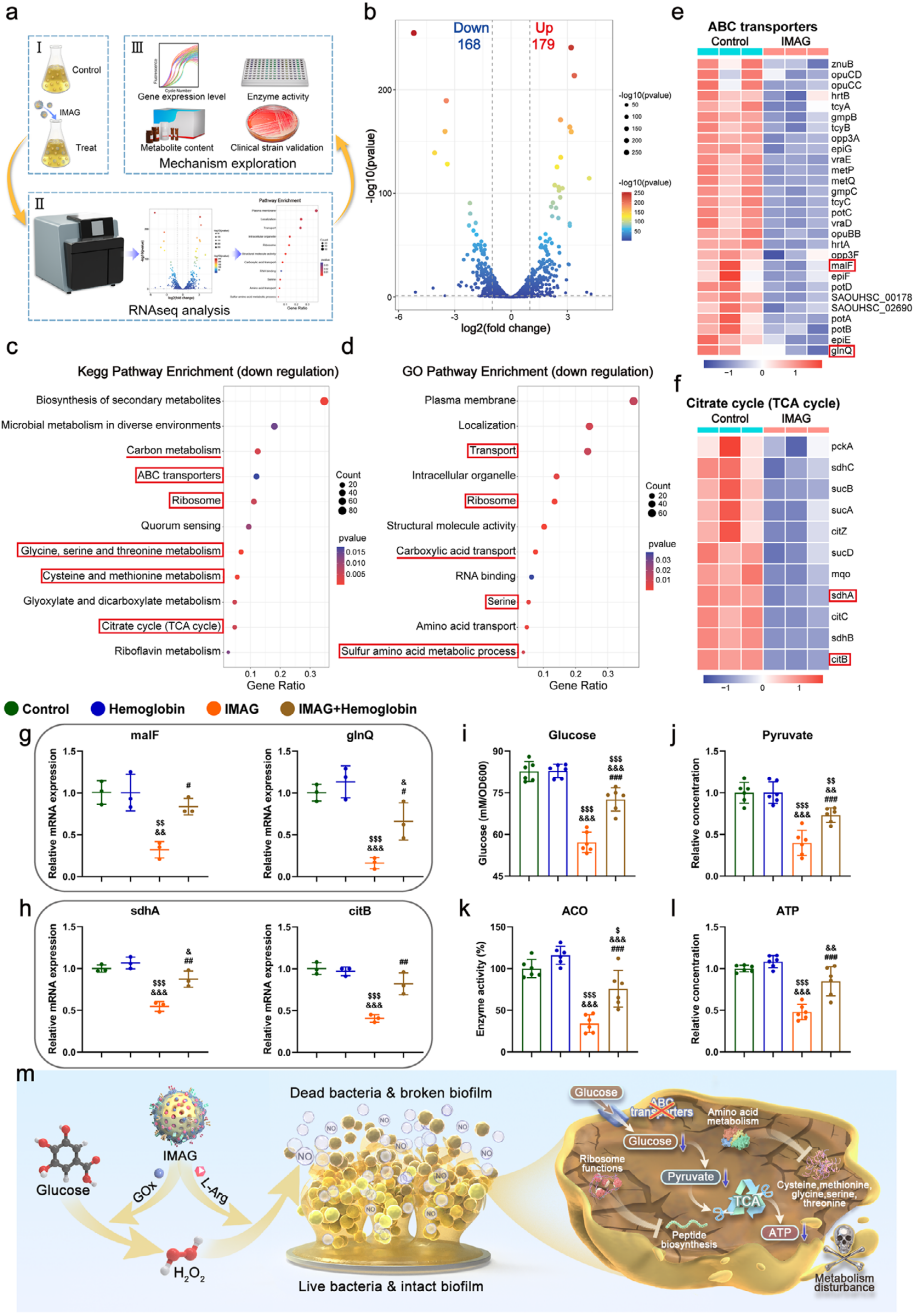
IMAG interference with *S. aureus* metabolism in vitro. (a) Experimental workflow exploring the antibacterial mechanism of IMAG. (b) Volcano plot illustrating differential gene expression of *S. aureus* between the IMAG‐treated and control groups. (c) KEGG enrichment analysis of downregulated genes in *S. aureus* after IMAG treatment. (d) GO enrichment analysis of downregulated genes in *S. aureus* after IMAG treatment. (e, f) Heatmaps of genes in the ABC transporters and citrate cycle pathways from KEGG enrichment analysis. (g, h) RT‐qPCR validation results for representative genes involved in ABC transporters (malF and glnQ) and citrate cycle (sadA and sucB) pathways (*n* = 3). (i) Glucose uptake in *S. aureus* with different treatments (*n* = 6). (j) Relative concentration of pyruvate within different groups of *S. aureus* (*n* = 6). (k) Activity of ACO in *S. aureus* (*n* = 6). (l) ATP level within *S. aureus* after different treatments (*n* = 6). (m) IMAG contributes to bacterial eradication and infection clearance by interfering with bacterial metabolism. Note: ^$^ *p *< 0.05, ^$$^ *p *< 0.01 and ^$$$^ *p *< 0.001 vs. the control group; ^&^ *p *< 0.05, ^&&^ *p *< 0.01 and ^&&&^ *p *< 0.001 vs. hemoglobin group; ^#^ *p *< 0.05, ^##^ *p *< 0.01 and ^###^ *p *< 0.001 vs. IMAG group. Data are presented as mean ± SD. Statistical significance was determined by one‐way ANOVA.

Typically, *S. aureus* utilizes ABC transporters to uptake nutrients like glucose, which are essential for sustaining basic metabolic activities and pathogenicity [40, 41, 42]. Genes for various TCA cycle enzymes, including succinate dehydrogenase (composed of sdhA, B, and C subunits), are usually upregulated in *S. aureus* biofilms [43, 44]. Increased TCA cycle activity not only optimizes oxygen consumption to produce more energy but also facilitates the reconsumption of metabolic byproducts such as acetate and lactate, preventing excessive acidification of the growth environment [16, 45]. Additionally, the TCA cycle is involved in the synthesis of various intermediate metabolites, which can indirectly affect biofilm formation by influencing the production of carbohydrates and proteins in the biofilm matrix [43]. Inhibiting the TCA cycle can reduce the energy available for physiological processes and interfere with signaling systems, thereby regulating biofilm development and antibiotic sensitivity [46, 47]. Moreover, the metabolism of amino acids like cysteine and methionine, as well as ribosomal pathways, plays a crucial role in bacterial activities. Methionine is essential for biofilm formation [48]. Cysteine is a precursor of glutathione (GSH). Inhibiting cysteine metabolism reduces GSH synthesis, thereby weakening the oxidative defense of *S. aureus* [49, 50]. Glycine, serine, and threonine are involved in the transfer and metabolism of one‐carbon units, which are vital for DNA synthesis and repair [51, 52]. Additionally, protein synthesis relies on these amino acids as precursors, along with the assistance of ribosomes. Based on these observations, we propose that NO generated by IMAG may contribute to the observed metabolic disruption in *S. aureus*, which is associated with impaired bacterial growth and biofilm formation. To prove this, *S. aureus* was treated with the NO scavenger hemoglobin (20 µM) alone or in combination with IMAG, and the expression of pathway‐related genes was assessed. The IMAG‐induced downregulation of genes related to ABC transporters, the TCA cycle, amino acid metabolism, and ribosomal function was alleviated by hemoglobin treatment (Figure 4g,h, and Figure S22b). Further metabolite analysis supported the interference of IMAG with *S. aureus* energy metabolism. As shown in Figure 4i, the glucose content in IMAG‐treated *S. aureus* decreased, potentially associated with decreased expression of ABC transporters. Additionally, pyruvate levels were significantly reduced (Figure 4j). The activities of SDH (encoded by the sdhA, sdhB, and sdhC genes) and ACO (encoded by the citB gene) in the TCA cycle were also inhibited (Figure 4k and Figure S23), suggesting consistent changes at both transcript and protein levels [53]. ATP levels in IMAG‐treated *S. aureus* were decreased (Figure 4l). Consistent metabolic disturbances were also observed in *E. coli* (Figure S24). Co‐treatment with hemoglobin largely reversed these effects, supporting that NO generated by IMAG mediates the observed metabolic perturbations. Further dose‐response and time‐course analyses demonstrated that NO generation by IMAG increased with increasing nanoparticle concentration and incubation time, accompanied by corresponding reductions in intracellular ATP levels (Figure S25), further supporting a dynamic association between IMAG‐mediated NO generation and bacterial metabolic suppression. Moreover, pyruvate supplementation partially restored ATP levels and biofilm biomass in IMAG‐treated *S. aureus* (Figure S26). Collectively, these results suggest that IMAG‐induced metabolic perturbations in bacteria contribute to reduced bacterial viability and impaired biofilm formation.

To further evaluate the translational relevance of IMAG, we next examined its antibacterial and anti‐biofilm efficacy against clinical isolates. Clinical *S. aureus* strains isolated from orthopedic infections, including five MSSA and five MRSA isolates, were randomly selected. Live/dead staining revealed that IMAG exerted potent and consistent bactericidal effects against all strains (Figure S27a). Representative gene analysis revealed that IMAG suppressed pathways involved in material transport, the TCA cycle, amino acid metabolism, and ribosomal function, with a marked decrease in intracellular ATP and inhibition of biofilm formation (Figure S27b–e). Moreover, co‐treatment with IMAG and vancomycin significantly reduced the minimum biofilm eradication concentration (MBEC) of vancomycin against *S. aureus* biofilms across multiple strains (Table S1), indicating that IMAG‐mediated metabolic interference weakens biofilm‐associated bacterial tolerance and enhances susceptibility to vancomycin treatment. Furthermore, considering that diabetic infections are frequently polymicrobial, we evaluated IMAG in a polymicrobial biofilm model consisting of *S. aureus* and *Pseudomonas aeruginosa* (*P. aeruginosa*). IMAG significantly reduced biofilm biomass and decreased the bacterial burden of both species (Figure S28).

Taken together, these results suggest the potential of IMAG‐based nano‐metabolic therapy, in which NO generated by IMAG functions as a bacterial metabolic regulator to disrupt energy metabolism and biofilm homeostasis. This confers consistent antibacterial and anti‐biofilm efficacy across different strains and enhances antibiotic susceptibility, highlighting its translational potential for the treatment of biofilm infections (Figure 4m).

### 2.4 Immunomodulatory Properties of IMAG In Vitro

Macrophages, as vital components of the innate immune system, play crucial roles in resisting pathogen invasion and maintaining tissue homeostasis [54, 55]. Therefore, the immunomodulatory effects of IMAG on macrophages were investigated. Macrophage activation states were operationally characterized using the classical M1/M2 framework to describe the overall pro‐inflammatory and anti‐inflammatory tendencies observed in vitro [56, 57]. Here, naïve (M0‐like) macrophages were used as the baseline state and were co‐cultured with different nanoparticles. Flow cytometry results showed that IMAG treatment significantly upregulated CCR7 expression (Figure 5a), indicating a shift toward a pro‐inflammatory phenotype [58]. In contrast, CD206 expression remained consistently low across all groups, suggesting preferential polarization toward a pro‐inflammatory phenotype without evident M2 activation. Consistently, immunofluorescence staining demonstrated increased CD86 expression and reduced Arg‐1 expression following IMAG treatment (Figure 5b). IMAG treatment also enhanced both the mRNA expression and protein secretion levels of inflammatory cytokines, as shown by RT‐qPCR and ELISA analyses (Figure 5c,d, and Figure S29). These results suggest that IMAG can effectively promote pro‐inflammatory activation of macrophages in vitro.

**FIGURE 5:**
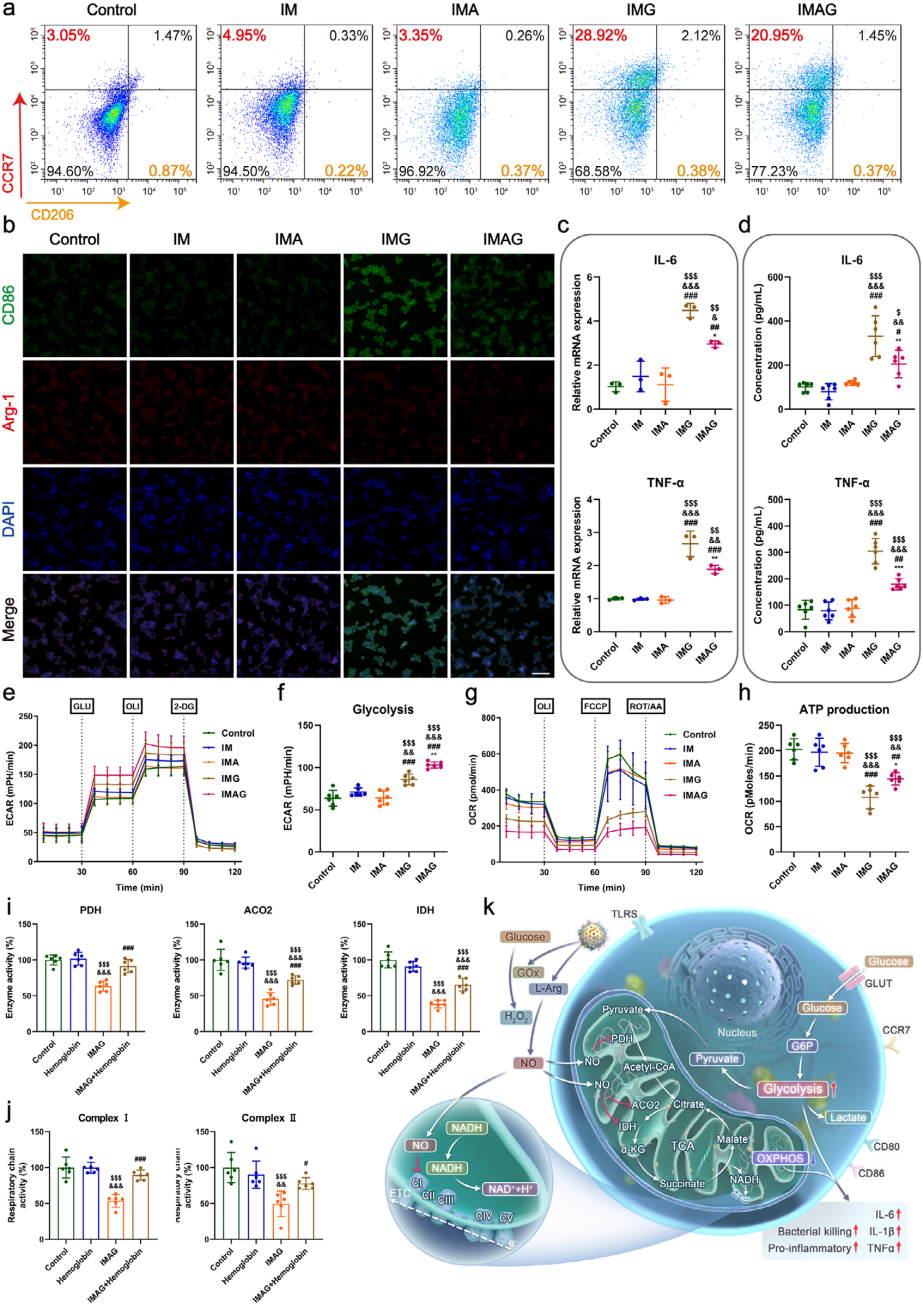
IMAG regulates the immunophenotype of macrophages by reshaping energy metabolism patterns. (a) Flow cytometry results for anti‐inflammatory macrophage (CD206) and pro‐inflammatory macrophage (CCR7). (b) Representative immunofluorescence images of macrophages stained for CD86 (green), Arg‐1 (red), and nuclei (DAPI, blue). Scale bar, 50 µm. (c) Gene expression levels of IL‐6 and TNF‐α in macrophages across different treatments (*n* = 3). (d) Cytokine secretion levels of IL‐6 and TNF‐α in macrophages for each treatment group (*n* = 6). (e) ECAR of macrophages at various time points. (f) Glycolysis in macrophages under various treatments (*n* = 6). (g) OCR of macrophages in each group. (h) ATP production in various groups of macrophages (*n* = 6). (i) Activity of PDH, ACO2, and IDH in macrophages (*n* = 6). (j) Mitochondrial electron transport chain complex I and complex II activity in macrophages after different treatments (*n* = 6). (k) IMAG modulates macrophage immune phenotype by reshaping metabolic processes. Note: ^$^ *p *< 0.05, ^$$^ *p *< 0.01 and ^$$$^ *p *< 0.001 vs. the control group; ^&^ *p *< 0.05, ^&&^ *p *< 0.01 and ^&&&^ *p *< 0.001 vs. IM or hemoglobin group; ^#^ *p *< 0.05, ^##^ *p *< 0.01 and ^###^ *p *< 0.001 vs. IMA or IMAG group; ^*^ *p *< 0.05, ^**^ *p *< 0.01 and ^***^ *p *< 0.001 vs. IMG group. Data are presented as mean ± SD. Statistical significance was determined by one‐way ANOVA.

As previously mentioned, changes in macrophage phenotype and immune function are regulated by cellular metabolism [59, 60]. The pro‐inflammatory and antibacterial functions of macrophages depend on aerobic glycolysis, whereas their anti‐inflammatory functions are driven by mitochondrial OXPHOS [4, 61]. Using a Seahorse metabolic analyzer, the metabolic characteristics of macrophages in each group were assessed. The extracellular acidification rate (ECAR) demonstrated a significant enhancement of aerobic glycolysis in the IMAG group, while the oxygen consumption rate (OCR) reflected a decrease in OXPHOS levels (Figure 5e‐h and Figure S30). These findings elucidate the shift to a pro‐inflammatory phenotype following IMAG treatment.

Next, the mechanisms by which IMAG alters macrophage metabolism patterns were investigated. Normally, glucose taken up by cells is enzymatically converted into pyruvate. The pyruvate then enters the mitochondria, where it is catalyzed by PDH to form acetyl‐CoA, subsequently entering the TCA cycle [62]. NADH generated in the TCA cycle is then oxidized by the respiratory chain, and the resulting electron transport drives OXPHOS to produce ATP [63]. IMAG likely impacts the activity of enzymes and respiratory chain complexes in this process, shifting metabolism patterns. The NO released from IMAG acts as the metabolic regulator, inhibiting key enzymes in the TCA cycle through several mechanisms, including modification of specific amino acid residues, destabilization of Fe‐S clusters, and nitrosylation of cysteine residues [64, 65, 66]. Additionally, NO exerts inhibitory effects on the respiratory chain complexes [67, 68]. Treating macrophages with 20 µM hemoglobin alone or in combination with IMAG, we subsequently assessed the activity of enzymes and respiratory chain complexes. IMAG treatment significantly inhibited PDH activity, leading to reduced carbon entry into the TCA cycle, while also suppressing ACO2 and IDH activities, which obstructed the conversion of citrate to α‐KG (Figure 5i). Assessments of respiratory chain complex I and II demonstrated similar trends (Figure 5j), and the reversal of activity by hemoglobin supported this hypothesis.

In conclusion, the NO generated from IMAG alters macrophage energy metabolism from OXPHOS to glycolysis by inhibiting the activities of TCA cycle‐related enzymes and respiratory chain complexes, thereby modulating macrophage immunophenotypes (Figure 5k). This metabolic reprogramming in macrophages, coupled with the disruption of bacterial metabolism, underscores the dual‐target effect of IMAG‐based nano‐metabolic therapy, enhancing infection clearance.

### 2.5 Effects of IMAG on Macrophage Phagocytosis and Antibacterial Functions In Vitro

To further investigate the properties of IMAG on macrophage immune functions, RNA‐seq analysis was conducted to compare the gene transcription profiles of macrophages treated with IMAG vs. those treated with an equal volume of PBS (control) (Figure 6a). IMAG treatment led to the upregulation of 1552 genes and downregulation of 1654 genes compared to the control group (Figure 6b). The heatmap of DEGs displayed significant heterogeneity between groups and high consistency within groups (Figure 6c). KEGG and GO pathway enrichment analyses showed that IMAG upregulated several antimicrobial pathways, including phagocytosis and the Toll‐like receptor signaling pathway (Figure 6d,e). Gene Set Enrichment Analysis (GSEA) of key pathways and heatmap analysis of pathway‐related genes further confirmed these results (Figures S31–S35).

**FIGURE 6:**
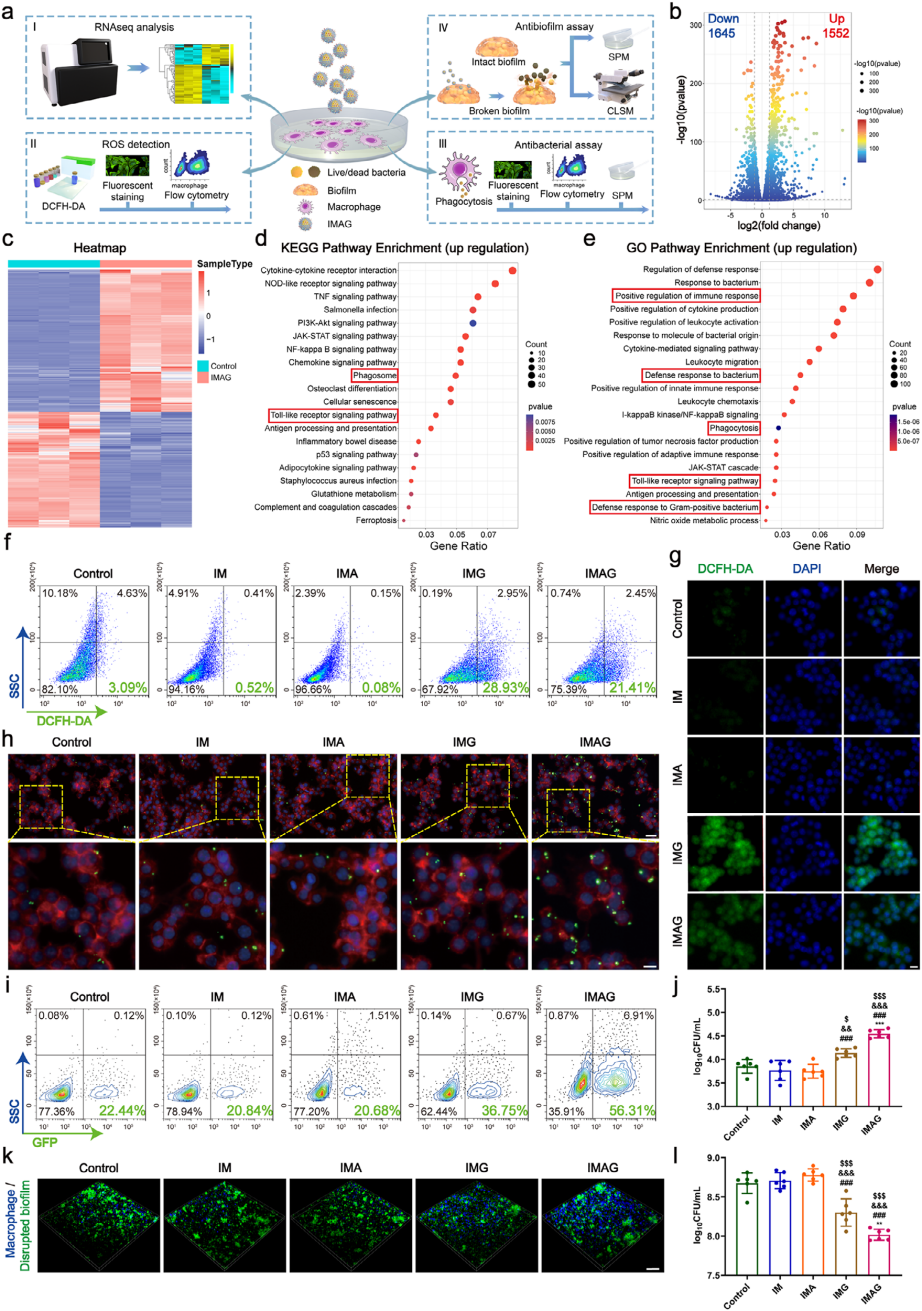
IMAG promotes antibacterial and antibiofilm functions of macrophages. (a) Diagram of the experimental workflow. (b) Volcano plot of differential gene expression in macrophages between IMAG‐treated and control groups. (c) Heatmap of DEGs in IMAG‐treated and untreated macrophages. (d) KEGG enrichment analysis showing upregulated pathways in macrophages after IMAG treatment. (e) GO enrichment result of upregulated pathways in macrophages after IMAG treatment. (f) Intracellular ROS levels detected by flow cytometry using the DCFH‐DA probe in various groups. (g) Representative fluorescence images of intracellular ROS stained with DCFH‐DA. Scale bar, 10 µm. (h) Immunofluorescence images showing macrophages phagocytosing GFP‐labeled *S. aureus*, with bacteria in green, macrophage cytoskeleton in red, and nuclei in blue. Scale bars, 25 µm and 10 µm. (i) Phagocytosis rates in macrophages analyzed by flow cytometry. (j) Quantification of macrophage phagocytic capacity using SPM (*n* = 6). (k) Representative CLSM images showing macrophage adhesion and invasion into IMAG‐pretreated biofilms in different groups, with *S. aureus* biofilms shown in green and macrophages in blue. Scale bar, 200 µm. (l) Quantification of bacterial burden in IMAG‐pretreated biofilms after co‐culture with different groups of macrophages (*n* = 6). Note: ^$^ *p *< 0.05 and ^$$$^ *p *< 0.001 vs. the control group; ^&&^ *p *< 0.01 and ^&&&^ *p *< 0.001 versus IM group; ^###^ *p *< 0.001 vs. IMA group; ^**^ *p *< 0.01 and ^***^ *p *< 0.001 vs. IMG group. Data are presented as mean ± SD. Statistical significance was determined by one‐way ANOVA.

Macrophages primarily rely on intracellular ROS for bacterial killing [69]. As detected by flow cytometry and fluorescence staining with ROS probe DCFH‐DA, the intracellular ROS level increased following IMAG treatment (Figure 6f,g), which was further supported by quantitative analysis (Figure S36). Additionally, immunofluorescence staining indicated that the IMAG treatment group exhibited the strongest phagocytic ability, with macrophages engulfing more *S. aureus ST8‐GFP* (Figure 6h), which was corroborated by flow cytometry and SPM analysis (Figure 6i,j). We then evaluated the ability of macrophages to invade and clear biofilms. *S. aureus ST8‐GFP* was cultured in the presence or absence of IMAG to form biofilms. The biofilms that were not treated with IMAG remained relatively intact (intact biofilm), while the biofilms treated with IMAG showed structural disruption (disrupted biofilm). Macrophages from each group were co‐cultured with either the intact biofilm or the disrupted biofilm. Although there was a slight increase in macrophage adhesion in the IMAG treatment group with the intact biofilm compared to other groups, SPM showed no significant difference in biofilm clearance (Figure S37). However, IMAG pre‐treatment loosened and disrupted the biofilm structure, resulting in a substantial increase in macrophage adhesion and biofilm clearance capacity in the IMAG treatment group (Figure 6k,l).

Macrophages can undergo anti‐inflammatory polarization in response to biofilm and bacterial virulence factors, leading to diminished bactericidal activity and suppressed pro‐inflammatory responses [70, 71]. This polarization hinders their ability to infiltrate biofilms and eliminate pathogens, resulting in chronic infections [4, 71]. Remodeling macrophage phenotypes toward a pro‐inflammatory state within the infected microenvironment may thus offer a promising approach for infection clearance [72]. Our results demonstrate the effectiveness of IMAG in immunophenotype reconstruction and immune function enhancement. Notably, although the IMG group showed a higher proportion of pro‐inflammatory macrophages, their aerobic glycolysis and OXPHOS levels were significantly lower than those in the IMAG group. Moreover, despite elevated intracellular ROS in the IMG group, their phagocytosis of planktonic bacteria and interaction with biofilms were inferior to those observed in the IMAG group. These differences may stem from the distinct products of IMG and IMAG. While IMG treatment results in excessive H_2_O_2_, which induces oxidative stress and impairs mitochondrial function [73], IMAG converts H_2_O_2_ into NO, mitigating toxicity. Mitochondrial membrane potential staining showed that IMG significantly reduced membrane potential, as indicated by JC‐1 monomer fluorescence (green), whereas IMAG preserved higher potential, suggesting better mitochondrial functionality (Figure S38). TEM imaging further confirmed mitochondrial damage in the IMG group, characterized by cristae disruption, membrane blurring, and vacuolization, in contrast to the intact mitochondrial morphology seen in the IMAG group (Figure S39). The above results elucidate the cause of changes in macrophage metabolism patterns, transitions in polarization phenotype, and alterations in immune function observed with IMG treatment. Although IMG promotes a pro‐inflammatory phenotype, the associated ROS overload causes mitochondrial dysfunction and metabolic collapse, ultimately impairing antibacterial capacity [74]. In contrast, IMAG preserves mitochondrial structure while inducing a controlled metabolic shift, as NO interferes with TCA cycle enzymes and respiratory chain complexes, thereby favoring a mild transition from OXPHOS to aerobic glycolysis. Importantly, under the bactericidal working concentration used in this study, this metabolic reprogramming promotes pro‐inflammatory polarization and enhances macrophage immune functions without compromising cellular viability. Collectively, these findings suggest that IMAG preserves macrophage activity while exerting antibacterial effects, thereby facilitating effective infection clearance. Moreover, IMG also showed the poorest outcomes in fibroblast migration and HUVEC angiogenesis, while IMAG significantly improved both processes, further indicating its potential utility in wound healing (Figures S40 and S41).

### 2.6 Evaluation of IMAG in Treating Diabetes‐Related Implant Infections

*S. aureus* biofilm infections represent a severe complication following orthopedic implant surgeries, particularly in diabetic patients [1]. To assess the in vivo anti‐infection and immunomodulatory effects of IMAG, a diabetic mouse model of implant‐associated infection was established (Figure 7a). Blood glucose levels were monitored throughout the experimental period, and all groups maintained stable hyperglycemia within the diabetic range without significant intergroup differences, indicating that the therapeutic outcomes were not associated with systemic glucose modulation (Figure S42). Real‐time monitoring of the infection progression was conducted. By day 4 postinfection, the control, MSN, and IMA groups developed pronounced back abscesses, which progressively enlarged and ultimately led to implant exposure (Figure S43). The IMG group demonstrated partial improvement; however, necrosis and ulceration persisted by day 14. In contrast, the IMAG group maintained intact skin throughout the observation period, with no visible tissue damage. Bioluminescence imaging revealed peak infection intensity in all groups on day 4, followed by a gradual decline. However, the control, MSN, and IMA groups remained significantly higher bioluminescence signals compared to the IMG and IMAG groups at all time points, with the IMAG group showing the lowest intensity (Figure 7b,c). On day 14, SEM imaging revealed dense bacterial aggregation on implant surfaces in the control, MSN, and IMA groups. Fewer bacteria were observed in the IMG group, while the IMAG group had minimal bacterial colonization (Figure 7d). These findings were corroborated by SPM quantification of bacteria extracted ultrasonically from the implants, as well as from surrounding tissues (Figure 7e). Giemsa staining further confirmed the reduced bacterial load in the IMAG group (Figure 7f), and H&E staining indicated notably decreased inflammatory cell infiltration (Figure 7g). Collectively, these results highlight the efficacy of IMAG in combating diabetes‐associated implant infections.

**FIGURE 7:**
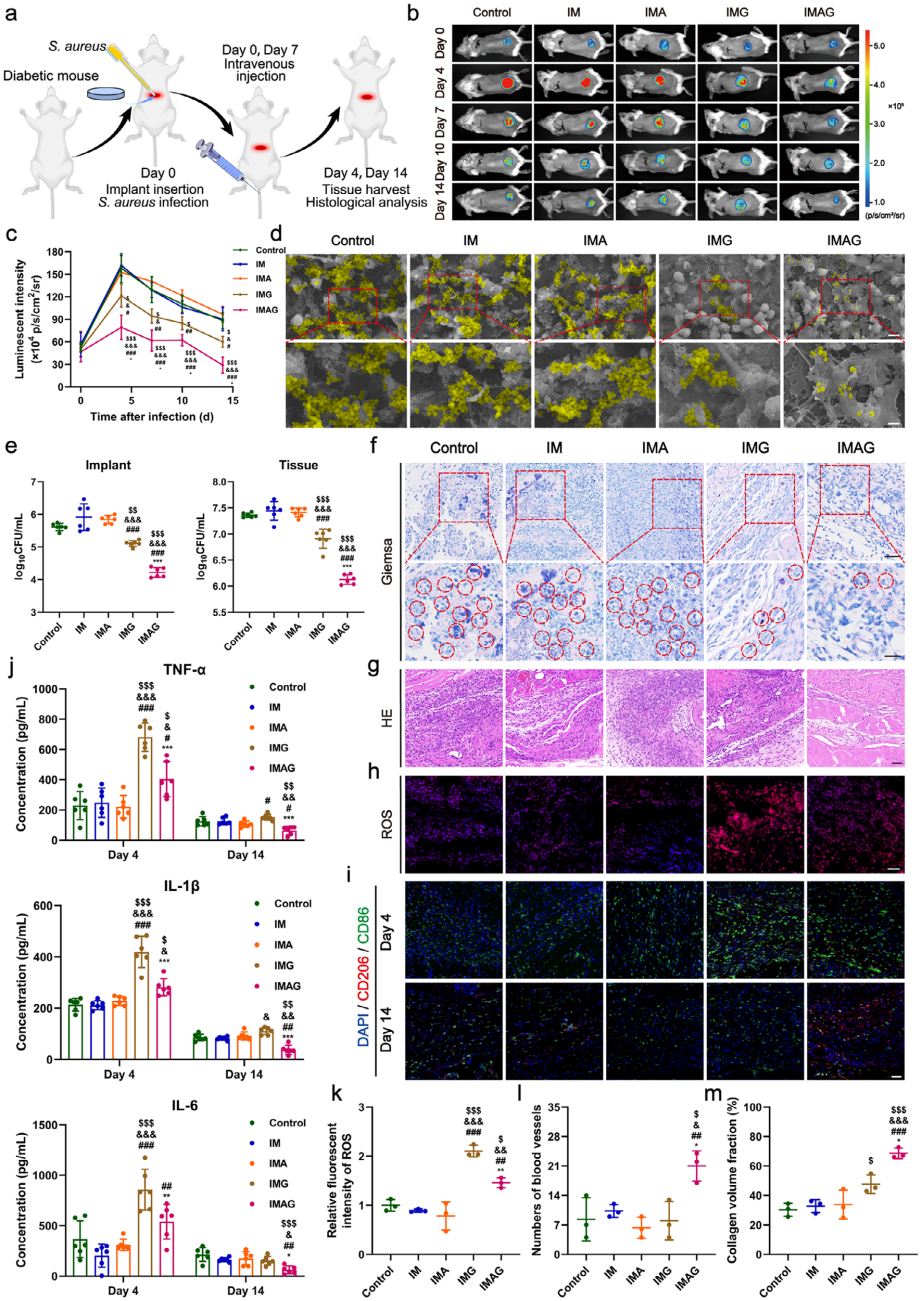
IMAG eliminates diabetes‐related implant‐associated infections in vivo. (a) Schematic diagram of the implant‐associated infection model construction and experimental workflow. (b) Representative bioluminescence images of mice from different groups on days 0, 4, 7, 10, and 14 after infection. (c) Time‐course curves of bioluminescence intensity at the infection sites in different groups (*n* = 3). (d) SEM images of implant surfaces on day 14 postinfection, with yellow indicating *S. aureus*. Scale bars, 5 µm and 2 µm. (e) Bacterial load within infected tissues and implants in different groups on day 14 post‐infection (*n* = 6). (f) Representative Giemsa staining images of infected tissues on day 14. Scale bars, 50 µm and 25 µm. (g) Representative H&E staining images on day 14 postinfection. Scale bar, 50 µm. (h, k) Representative ROS staining images and quantification of relative fluorescence intensity of infected peri‐implant tissues on day 4 post‐infection. Scale bar, 50 µm (*n* = 3). (i) Immunofluorescence images of macrophage markers CD86 (green) and CD206 (red) in infected peri‐implant tissues on days 4 and 14 postinfection. Scale bar, 50 µm. (j) ELISA results showing cytokine levels in infected peri‐implant tissues from different groups on days 4 and 14 postinfection (*n* = 6). (l) Quantification of newly formed blood vessels surrounding the implants on day 14 (*n* = 3). (m) Collagen deposition in peri‐implant tissues on day 14 (*n* = 3). Note: ^$^ *p *< 0.05, ^$$^ *p *< 0.01 and ^$$$^ *p *< 0.001 vs. the control group; ^&^ *p *< 0.05, ^&&^ *p *< 0.01 and ^&&&^ *p *< 0.001 vs. IM group; ^#^ *p *< 0.05, ^##^ *p *< 0.01 and ^###^ *p *< 0.001 vs. IMA group; ^*^ *p *< 0.05, ^**^ *p *< 0.01 and ^***^ *p *< 0.001 vs. IMG group. Data are presented as mean ± SD. Statistical significance was determined by one‐way ANOVA.

We further assessed the immunomodulatory effects of IMAG in infected mice. Immunofluorescence staining showed that by day 4 postinfection, the ROS level in the IMAG group was higher than that in the control, MSN, and IMA groups (Figure 7h,k). This was accompanied by increased accumulation of pro‐inflammatory macrophages and elevated expression of TNF‐α, IL‐1β, and IL‐6 on day 4 (Figure 7i,j). Although the IMG group exhibited the highest ROS levels and the largest proportion of pro‐inflammatory macrophages at this time point, excessive oxidative stress ultimately hindered immune functions and impaired infection clearance. By day 14, macrophages in the IMAG group transitioned to an anti‐inflammatory phenotype, with reduced inflammatory factors and enhanced tissue regeneration (Figure 7i,j). This was evidenced by the most pronounced neovascularization and collagen deposition (Figure 7l,m, and Figures S44 and S45). In contrast, by day 14, macrophages in the control, MSN, and IMA groups remained in a pro‐inflammatory state due to persistent infection, accompanied by sustained inflammatory cytokines and impaired tissue repair. While the IMG group showed moderate improvement in bacterial clearance, excessive ROS accumulation continued to compromise macrophage function, prolong the inflammatory state, and inhibit tissue repair (Figure 7i,j).

To further evaluate the contribution of macrophages to the therapeutic efficacy of IMAG in vivo, a macrophage‐depleted diabetic implant infection model was established using clodronate liposomes (CLO). As shown in Figure S46, CLO treatment alone led to more severe infection and partial mortality by day 14. Although IMAG treatment in CLO‐depleted mice (CLO‐IMAG) still reduced bacterial load compared to the CLO group, its antibacterial efficacy was substantially attenuated in CLO‐treated mice compared to immunocompetent mice (IMAG vs CLO+IMAG). These findings suggest that IMAG exerts both direct antibacterial activity and macrophage‐mediated immunotherapeutic effects in vivo, together contributing to infection clearance through a synergistic mechanism driven by GOx‐mediated NO generation, which in turn mediates bacterial metabolic disruption and macrophage immune activation under hyperglycaemic conditions.

Current strategies for combating implant infections primarily rely on generating endogenous or exogenous ROS for pathogen killing [75, 76, 77]. While these approaches show promising in vitro efficacy, their in vivo effectiveness remains limited. This discrepancy arises from the dual‐edged nature of oxidative stress and the phenotypic plasticity of macrophages [78]. While ROS promotes bacterial killing and amplifies macrophage‐mediated inflammation during early infection, persistent oxidative stress inhibits macrophage transition to an anti‐inflammatory phenotype once the infection is under control, thereby impairing tissue regeneration [79, 80]. This dilemma is exemplified in our implant infection model, where IMG exploits the high‐glucose microenvironment to catalyze H_2_O_2_ production for antibacterial action but fails to facilitate subsequent tissue repair. Conversely, IMAG leverages GOx‐mediated H_2_O_2_ generation alongside Arg supplementation to convert H_2_O_2_ into NO. This catalytic cascade effectively reconciles the conflict between bacterial eradication and tissue regeneration.

### 2.7 Evaluation of IMAG in Treating Biofilm‐Associated Wound Infections in Diabetic Mice

In addition to implant‐related infections, skin ulcers are common complications in diabetic patients, with biofilm‐associated infections posing significant clinical challenges [2]. To evaluate the therapeutic effects of IMAG on infected wounds, a biofilm‐related wound infection model was established on the backs of diabetic mice (Figure 8a). Blood glucose levels remained stable within the diabetic range throughout the experimental period, with no significant intergroup differences observed (Figure S47). As shown in Figure S48, the IMAG group exhibited less purulent discharge compared to the other groups. Bacterial loads on days 4 and 14 postsurgery were lower in the IMAG group than in the others (Figure 8b), consistent with Giemsa staining results (Figure S49). Mirroring findings from implant infections, the IMAG group had higher ROS fluorescence intensity on day 4 compared to the control, MSN, and IMA groups (Figure S50), with increased accumulation of pro‐inflammatory macrophages at the infection site (Figure S51) and elevated secretion of inflammatory factors that promote immune cell recruitment and bacterial clearance (Figure S52). During later wound healing stages, macrophages in the IMAG group polarized more rapidly towards an anti‐inflammatory phenotype, with decreased expression of inflammatory factors compared to the other four groups. These results suggest that IMAG effectively regulates macrophage immune function and eliminates infections in diabetic wound models.

**FIGURE 8:**
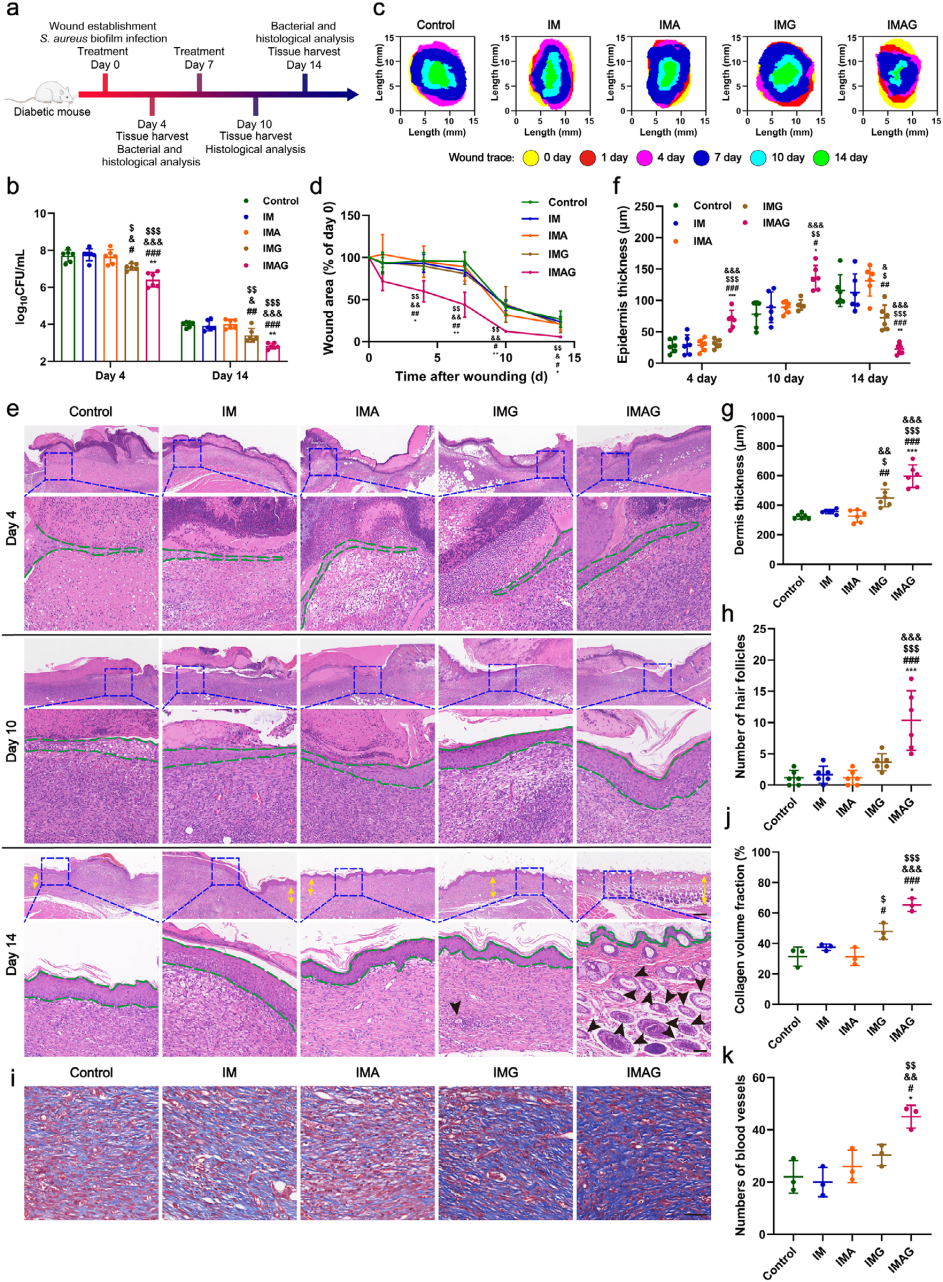
IMAG promotes the healing of biofilm‐associated diabetic wounds. (a) Schematic diagram of the construction and experimental workflow of the biofilm‐associated wound infection model. (b) Bacterial load in infected tissues on days 4 and 14 post‐infection (*n* = 6). (c) Overlay images of the wound area at different time points post‐infection across various groups. Scale bar, 15 mm. (d) Wound healing progression over time following different treatments (*n* = 3). (e) H&E staining images illustrating wound healing progress on days 4, 10, and 14 postsurgery. Green dashed lines indicate epithelium thickness; black arrows point to hair follicles; yellow arrows denote dermal thickness. Scale bars, 250 µm and 50 µm. (f) Quantification of epithelial thickness on days 4, 10, and 14 postsurgery (*n* = 6). (g) Dermal thickness in different groups on day 14 postsurgery (*n* = 6). (h) Number of hair follicles on day 14 postsurgery (*n* = 6). (i) Representative Masson staining images on day 14 postsurgery. Scale bar, 50 µm. (j) Quantitative analysis of collagen deposition in different groups (*n* = 3). (k) The Number of newly formed blood vessels on day 14 postsurgery (*n* = 3). Note: ^$^ *p *< 0.05, ^$$^ *p *< 0.01 and ^$$$^ *p *< 0.001 vs. the control group; ^&^ *p *< 0.05, ^&&^ *p *< 0.01 and ^&&&^ *p *< 0.001 versus IM group; ^#^ *p *< 0.05, ^##^ *p *< 0.01 and ^###^ *p *< 0.001 vs. IMA group; ^*^ *p *< 0.05, ^**^ *p *< 0.01 and ^***^ *p *< 0.001 versus IMG group. Data are presented as mean ± SD. Statistical significance was determined by one‐way ANOVA.

Qualitative and quantitative analyses of wound healing revealed that, while all infected wounds gradually healed over time, the IMAG group exhibited a significantly faster healing rate than the other groups (Figure 8c,d). H&E staining at various time points post‐surgery showed inflammatory infiltration in all groups; however, the IMAG group demonstrated a more rapid resolution of inflammation. In contrast, persistent inflammatory infiltration was observed in the control, MSN, and IMA groups due to inadequate infection control, while the IMG group showed delayed resolution due to oxidative stress‐induced tissue damage. Normally, the healing process involves the gradual thickening of the new epidermis, followed by a reduction to normal thickness, alongside the progressive thickening of the dermis and an increase in skin appendages such as hair follicles and blood vessels [81]. Histological staining indicated that the IMAG group most closely followed this trajectory, exhibiting the thickest dermis and the highest number of hair follicles by the end of observation (Figure 8e–h). Similar trends were observed for collagen deposition and neovascularization, both of which were markedly enhanced in the IMAG group compared to the others (Figure 8i–k and Figure S53). These results underscore the superior wound healing efficacy of IMAG in the context of diabetic infection

The biofilm matrix creates a thick physical barrier that impedes the infiltration of immune cells and the penetration of antimicrobial agents [4]. Although various methods have been developed to disrupt biofilm structures, bacterial dissemination and extensive colonization can lead to sepsis [8, 9]. Moreover, the immune system is inherently dysregulated and suppressed in diabetic patients, and bacteria within biofilms further impair immune cells through the secretion of virulence factors and modulation of quorum sensing systems [82, 83]. Emerging approaches, including sonodynamic, photodynamic, and photothermal therapies, have been explored for enhancing immune responses [84, 85, 86, 87]. However, these methods primarily rely on exogenously generated ROS, which often leads to short treatment durations and potential irreversible damage to normal tissues, ultimately impeding tissue healing. Therefore, there is an urgent need for new intervention strategies that simultaneously combat biofilms and gently enhance immune function. The outcomes of the IMAG‐treated diabetic mice demonstrate the effectiveness of the dual‐target metabolism interference strategy for converting immune function and eliminating infections. Moreover, no significant elevation of 3‐nitrotyrosine (3‐NT), a marker associated with nitrosative stress, was observed following IMAG treatment, suggesting the absence of overt systemic nitrosative toxicity under the current experimental conditions (Figure S54). Histological examination of major organs in mice treated with various nanoparticles revealed no substantial lesions, and biochemical markers showed no significant differences compared to the control group (Figures S55 and S56). Coagulation parameters and serum complement levels (C3a, C5a) were also comparable across all groups, indicating that IMAG does not induce complement activation or affect coagulation (Figure S57). Overall, these results indicate favorable in vivo biocompatibility of IMAG and support its translational potential as a nanoplatform‐based therapeutic strategy.

In this study, preinfected macrophage membranes were used to coat the nanoplatform. Systematic characterization demonstrated that this strategy significantly enhanced bacterial adhesion, infection‐site accumulation, and in vivo retention. These findings are consistent with previously reported activated or bacteria‐primed macrophage membrane strategies [88, 89, 90], while the present work further provides a direct head‐to‐head comparison with unmodified macrophage membranes in both in vitro and in vivo settings. Collectively, these results highlight the infection‐adaptive nature of the membrane coating and its role in improving targeting efficiency in diabetic biofilm‐associated infections. More importantly, the present design integrates infection targeting with coordinated regulation of bacterial metabolism and macrophage immunometabolism, thereby enabling dual‐target therapeutic modulation in diabetic biofilm‐associated infections.

The dosing regimen used in this study was optimized for consistent local exposure and model reproducibility in a preclinical setting. Future translational studies will require further optimization of dosing strategies, administration schedules, and comparison with clinically established antimicrobial delivery systems. Nevertheless, the current regimen provides a useful experimental reference for IMAG‐based nano‐metabolic therapy in diabetic infection models.

Although the therapeutic potential of IMAG in streptozotocin‐induced diabetic infection models was explored, we acknowledge that this model may not fully recapitulate the complex metabolic and immune characteristics of human type 2 diabetes. Future studies using db/db or high‐fat diet‐induced diabetic models will help further strengthen the translational potential of this nano‐metabolic therapeutic strategy. In addition, further evaluation of the long‐term biosafety and immunological effects of IMAG, including potential immune memory responses and systemic immune bias under repeated‐administration conditions, will be important for future translational development.

## 3 Conclusions

In summary, this study proposes a biomimetic nanoplatform‐based dual‐target nano‐metabolic therapy driven by the metabolic regulator for the treatment of diabetes‐related biofilm infections. By utilizing biomimetic nanotechnology, MSNs are camouflaged with membranes derived from *S. aureus*‐infected macrophages, enabling targeted drug delivery and enhanced accumulation at infection sites through improved bacterial recognition. Within the high‐glucose microenvironment, GOx loaded into the nanoparticles catalyzes the production of H_2_O_2_, which subsequently triggers the localized release of NO from Arg. This cascade not only avoids excessive oxidative stress but also modulates bacterial and host metabolism to facilitate infection eradication. Specifically, NO disrupts the TCA cycle and amino acid metabolism in bacteria, impairing biofilm formation and bacterial viability. Meanwhile, NO reprograms macrophage metabolism from OXPHOS toward aerobic glycolysis, reversing immune suppression and promoting antibacterial function. This dual‐target nano‐metabolic therapy represents a promising therapeutic paradigm for biofilm‐associated infections in diabetic settings, with potential implications for broader applications in infection‐immunometabolism.

## 4 Materials and Methods

### 4.1 Preparation and Characterization of MSN and MSN@Arg@GOx

As described previously [36], pristine mesoporous silica nanoparticles (MSNs) were prepared with slight modifications using the sol‐gel method, with CTAB as the pore template. Initially, 1.82 g of CTAB and 3.0 g of NH_4_F were added to 500 mL of ultrapure water. The mixture was stirred at 80°C for 30 min, after which 9 mL of tetraethyl orthosilicate (TEOS) was added dropwise, and the reaction was continued for 2 h. The particles were collected by centrifugation at 8000 rpm for 10 min, washed with anhydrous ethanol, and then calcined in a muffle furnace at 550°C for 24 h to remove residual CTAB. Fluoride and bromide levels in the nanoparticles were quantified before and after calcination to verify effective removal of NH_4_F‐ and CTAB‐derived residues. Next, 0.5 g of the nanoparticles was dispersed in 8 mL of ethanol, stirred at 50°C for 30 min, and 0.2 mL of (3‐aminopropyl)triethoxysilane (APTES) was added, followed by reflux condensation at 40°C for 8 h to introduce a sparse layer of surface amino groups for subsequent GOx immobilization. The functionalized MSNs were then collected by centrifugation, washed, and freeze‐dried.

The zeta potential of pristine MSNs (pMSNs) and amino‐functionalized MSNs was measured using a Malvern ZS‐90 (Malvern, UK). To assess protein‐corona formation, nanoparticles were incubated with mouse serum at 37°C for 2 h, followed by washing and quantification of adsorbed proteins using the BCA Protein Assay Kit (Epizyme, China). Hemolysis and complement activation assays were performed as described in the corresponding sections.

The encapsulation efficiency (EE) and loading content (LC) of glucose oxidase (GOx) and arginine (L‐Arg) were determined at different feeding ratios (drug: nanoparticle). GOx and Arg were quantified by BCA assay and UV–Vis, respectively. EE and LC were calculated as:

$$\mathrm{Encapsulation}\;\mathrm{efficiency}\;(\%)=((M_{i}-M_{0})/M_{i})\times 100\%$$

$$\mathrm{Loading}\;\mathrm{content}\;(\mathrm{mg}/\mathrm{mg})=(M_{i}-M_{0})/(M_{i}-M_{0}+M_{t})$$

where M*_i_* is the initial drug amount, M_0_ is the amount of unloaded drug, and M*_t_* is the mass of nanoparticles. The optimal feeding ratios were selected based on maximal EE and LC and used for subsequent nanoparticle preparation.

To prepare MSN@Arg@GOx, 4 mg of GOx (50 kU mg^−1^), 38 mg of 1‐ethyl‐3‐(3‐dimethylaminopropyl)carbodiimide (EDC), and 57 mg of N‐hydroxysuccinimide (NHS) were dissolved in 6 mL of ultrapure water, followed by the addition of 45 µL of APTES. The mixture was stirred at room temperature for 8 h to obtain amino‐functionalized GOx. Next, 4 mL of ultrapure water containing 20 mg of MSNs was added and stirred for an additional 24 h to promote the conjugation of GOx with MSNs (MSN@GOx). After collecting the MSN@GOx nanoparticles by centrifugation, 20 mg of MSN@GOx was dissolved in 10 mL of ultrapure water along with 200 mg of arginine (L‐Arg). The mixture was stirred at room temperature for 24 h to ensure that L‐Arg penetrated and was evenly dispersed within the porous structure of MSN@GOx. Finally, MSN@Arg@GOx was obtained by centrifugation and freeze‐drying.

The morphology of the nanoparticles was characterized using scanning electron microscopy (SEM, ZEISS GeminiSEM 300, Germany) and transmission electron microscopy (TEM, Hitachi‐7800, Japan). Elemental mapping was employed to evaluate the elemental distribution in the nanoparticles. The drug‐loading capacity of the nanoparticles was analyzed using a Fourier‐transform infrared (FT‐IR) spectrometer (Tensor 27, Germany) and a UV–visible spectrophotometer (UV1600, China). The specific surface area before and after drug loading was determined by nitrogen adsorption‐desorption analysis (Micromeritics ASAP 2010, USA) using the Brunauer–Emmett–Teller (BET) method. The Zeta potential and particle size of the nanoparticles were subsequently measured. The release profiles and half‐lives of GOx and L‐Arg were further examined at 37°C under varying pH, ionic strength, and FBS conditions.

### 4.2 Cell Culture

Mouse macrophages RAW264.7, mouse fibroblasts L929, and human umbilical vein endothelial cells (HUVECs) were separately cultured in high‐glucose Dulbecco's Modified Eagle Medium (DMEM, Gibco) at 37°C in a 5% CO_2_ humidified incubator. The medium was supplemented with 1% penicillin‐streptomycin solution (Beyotime, China) and 10% fetal bovine serum (FBS, Gibco).

### 4.3 Bacterial Culture and Biofilm Formation

The *Staphylococcus aureus* (*S. aureus*) strain *ST8* and Escherichia coli (*E. coli*) strain *BL21* were used in this study. The related fluorescent and bioluminescent strains, *ST8‐GFP* (green fluorescent strain) and *ST8‐lux* (bioluminescent strain), were constructed in our laboratory. All strains were stored at −80°C. Before use, bacteria were plated on blood agar plates and incubated overnight at 37°C. For each experiment, bacteria were grown overnight in tryptic soy broth (TSB) at 37°C and then diluted to the desired concentration with TSB, PBS, or DMEM. For biofilm formation, bacteria were diluted to 10^6^ colony‐forming units (CFU) mL^−1^ in TSB containing 0.25% glucose (TSBG) and inoculated into 24‐well plates with silicon flakes. After overnight incubation at 37°C, the TSBG was removed, and planktonic bacteria were gently washed away with PBS, leaving the biofilm for subsequent experiments.

### 4.4 Detection of TLR Receptors on Macrophage Membranes

RAW264.7 macrophages (1 × 10^6^ cells per well) were seeded in a 6‐well plate and co‐cultured with *S. aureus* (MOI = 10:1) for 2 h. The macrophages were then washed three times with PBS to remove bacteria. Cells were collected and stained on ice with anti‐mouse TLR2, TLR4, and TLR6 antibodies (1:50, Bio Legend, USA) for 30 min in the dark. After washing, the expression of TLR2, TLR4, and TLR6 on the macrophage membranes was analyzed by flow cytometry.

### 4.5 Extraction of Macrophage Membranes

Live *S. aureus* was used as the activating stimulus to induce macrophage membrane activation through simultaneous engagement of multiple pattern‐recognition pathways. As described previously [91], RAW264.7 cells (1 × 10^6^ cells per well) were co‐cultured with *S. aureus* (MOI = 10:1) for 2 h, followed by thorough washing with PBS and treatment with gentamicin (100 µg mL^−1^, 1 h) to eliminate residual bacteria. The cells were then collected by centrifugation at 1000 rpm for 5 min and resuspended in ice‐cold Tris‐magnesium buffer (TM buffer, pH 7.4, 0.01 M Tris and 0.001 M MgCl_2_) containing 1% protease inhibitor phenylmethylsulfonyl fluoride (PMSF, Beyotime, China). Cells were lysed by ultrasonication on ice (5 cycles, 30s each). The lysate was diluted with 1 M sucrose solution to achieve a final concentration of 0.25 M, followed by centrifugation at 2000 g for 10 min at 4°C. The resulting supernatant was then subjected to further centrifugation at 20 000 g for 30 min at 4°C to isolate the macrophage membranes. The membranes were rinsed twice with ice‐cold TM buffer containing 0.25 M sucrose and centrifuged again under the same conditions before being stored at −80°C for future use.

### 4.6 Preparation and Characterization of IMAG Nanoparticles

For IMAG preparation, pre‐infected macrophage membranes were mixed with MSN@Arg@GOx. The membranes were coated onto the nanoparticles using ultrasonication (100 W, 5 min). The mixture was then extruded repeatedly through 200 nm polycarbonate porous membranes using an extruder (Avanti Polar Lipids, USA). Excess membranes were removed by centrifugation at 12 000 rpm for 5 min, and the resulting IMAG nanoparticles were collected and resuspended in PBS at 4°C for storage and further use [92].

The coating of pre‐infected macrophage membranes on the nanoparticles was observed using TEM. The hydrodynamic diameter and zeta potential of IMAG nanoparticles were measured. To assess membrane protein content, samples were run on 10% SDS‐PAGE (Epizyme, China) at 120 V for 50 min, stained with Coomassie Brilliant Blue (Beyotime, China) for 1 h, washed, and imaged. Western blotting was performed to detect CD11b, Na^+^/K^+^‐ATPase, GAPDH, and Histone H3. Whole‐cell lysates and isolated macrophage membranes served as controls. To assess the catalytic efficiency, nanoparticles were co‐incubated with glucose (4 mg mL^−1^). The concentrations of H_2_O_2_ and NO over time were measured and plotted to create time‐dependent curves.

### 4.7 Culture‐Based sterility testing

PBS (negative control), IM suspensions, and *S. aureus* (1 × 10^7^ CFU mL^−1^, positive control) were plated on blood agar and incubated at 37°C for 24 h. In parallel, samples were inoculated into TSB medium and cultured at 37°C to monitor bacterial growth over time.

### 4.8 Bacterial Adhesion Capability

To assess the bacterial adhesion capability of different nanoparticles, *S. aureus* and *E. coli* were fixed with 4% paraformaldehyde, washed with PBS, and resuspended, respectively. Subsequently, 500 µg of FITC‐labeled nanoparticles were added to the bacterial suspension, with PBS as the blank control. After incubation at 37°C for 1 h, the binding efficiency of nanoparticles to bacteria was quantified by flow cytometry.

### 4.9 In Vitro Cytotoxicity Assessment

To evaluate the cytotoxicity of the nanoparticles, RAW264.7 and L929 cells were seeded into 24‐well plates at a density of 10^5^ cells per well and then co‐cultured with different groups of nanoparticles for 24 h. PBS‐treated cells served as the control. Subsequently, cells were washed with PBS and incubated with 10% CCK‐8 reagent for 2 h, and the absorbance at 450 nm was measured using a microplate reader (BIO‐TEK, ELX 800). Additionally, cells were stained with the Calcein/Propidium Iodide Cell Viability/Cytotoxicity Assay Kit (Beyotime, China) for 30 min in the dark and visualized using a fluorescence microscope.

For the observation of cell morphology, after co‐culture with nanoparticles, different groups of cells were fixed, permeabilized, and stained with Actin‐Tracker Red 555 for the cytoskeleton and DAPI for the nucleus. The stained cells were then observed under a fluorescence microscope.

Hemolysis assays were also used to assess the biocompatibility of the nanoparticles. Briefly, fresh mouse blood was diluted 10‐fold with PBS containing an anticoagulant and then incubated with the nanoparticles at 37°C for 2 h. PBS‐treated samples served as the negative control, while 1% Triton‐X100‐treated samples served as the positive control. After incubation, the samples were centrifuged at 3000 rpm for 5 min. Photographs were taken, and the supernatants were collected to measure the absorbance at 540 nm using a microplate reader. The hemolysis percentage was calculated as follows:

$$\begin{array}{ccc} & & \mathrm{Hemolysis}\;\mathrm{ratio}\;(\%)\\ & & \;=\left({\mathrm{OD}}_{\mathrm{sample}}-OD_{\mathrm{PBS}}\right)/\left(OD_{\mathrm{Triton}-X100}-OD_{\mathrm{PBS}}\right)\times 100\% \end{array}$$

### 4.10 Antibacterial Activity In Vitro

#### 4.10.1 Bactericidal Activity In Vitro

Different groups of nanoparticles were co‐cultured with bacteria (1 × 10^6^ CFU mL^−1^) at 37°C for 24 h, with PBS used as the control, to assess the antibacterial effects. Absorbance at 600 nm was measured for each bacterial suspension at 0, 2, 4, 6, 8, 10, 12, and 24 h using a microplate reader, and bacterial growth curves were plotted. The bacterial suspensions were also collected to evaluate the antibacterial effects of the nanoparticles using the spread plate method (SPM). Briefly, the bacterial suspensions from each group were centrifuged to remove the supernatant, and the pellets were resuspended in sterile PBS, followed by serial dilution. Next, 100 µL of the dilution was evenly spread onto blood agar plates, and colony counts were determined after 24 h of incubation at 37°C. For qualitative observation, bacterial suspensions from each group were collected after co‐incubation, centrifuged to remove the supernatant, and stained with LIVE/DEAD BacLight Bacterial Viability Kits (Invitrogen, USA) for 30 min in the dark. After removing excess dye by centrifugation, the bacterial pellets were resuspended in PBS and observed under a fluorescence microscope. Live bacteria exhibited green fluorescence, while dead bacteria showed red fluorescence. Additionally, flow cytometry was performed on the stained samples to quantitatively analyze the ratio of live and dead bacteria.

#### 4.10.2 Anti‐Biofilm Efficacy In Vitro

Different groups of nanoparticles were co‐cultured with bacterial suspensions (1 × 10^6^ CFU mL^−1^) in TSBG, with the PBS‐treated group serving as the control. After removing the supernatant and washing away planktonic bacteria with sterile PBS, biofilms were stained with LIVE/DEAD BacLight Bacterial Viability Kits (Invitrogen, USA) for 30 min in the dark. The biofilm was then observed using a confocal laser scanning microscope (CLSM) (Leica, Germany). Crystal violet staining was used to assess biofilm biomass. Biofilms were fixed with methanol and stained with 0.1% crystal violet, each for 15 min, and then dissolved in 33% acetic acid. The absorbance of the resulting solution at 595 nm was measured. Additionally, SPM was employed to quantify the bacterial load in the biofilms. For microscopic examination of biofilm morphology, biofilms were fixed at 4°C for 8 h, dehydrated through a graded ethanol series (50%, 60%, 70%, 80%, 90%, and 100% v/v, each for 10 min), freeze‐dried for 8 h, and then sputter‐coated with platinum. The biofilms were finally observed via SEM.

### 4.11 RNA‐Sequencing (RNA‐seq) Analysis for Bacteria and Macrophages

RNA‐seq was conducted to analyze the transcriptomic profiles of *S. aureus ST8* and RAW 264.7 macrophages treated with IMAG compared to controls. Sample preparation followed protocols provided by Novogene Co., Ltd (Beijing, China), which also performed RNA extraction, quality control, and library construction. Sequencing was performed on the NovaSeq‐PE150 platform (Novogene, China). The resulting data were processed using the DESeq2 package, identifying differentially expressed mRNAs with thresholds of |log2(Fold change)| > 1 and *p* < 0.05. Visualization of gene expression differences was achieved through volcano plots and heatmaps. Enrichment analyses for functional and pathway associations were carried out using the Gene Ontology (GO) and Kyoto Encyclopedia of Genes and Genomes (KEGG) databases, with significant results (*p* < 0.05) displayed in bubble plots. Furthermore, Gene Set Enrichment Analysis (GSEA) was performed to assess pathway‐specific gene expression. All data analysis was completed using the Novomagic platform (https://magic.novogene.com/customer/main#/login). The sequencing data generated in this study have been deposited in the NCBI Sequence Read Archive (SRA) under accession codes PRJNA1471373 and PRJNA1471368.

### 4.12 Bacterial and Cellular RNA Extraction and Quantitative Real‐Time PCR (RT‐qPCR)

After co‐culturing, bacteria from each group were collected and transferred to a tissue lyser (ScientzTM, Ningbo, China). Samples were disrupted at 70 Hz for 40 s at 4°C, repeated three times to break bacterial cell walls. Total bacterial RNA was extracted using the Ezscript Reverse Transcription Kit (EZBioscience, USA), while total cellular RNA was extracted using the RNA Extraction Column Kit (EZBioscience, USA) following the manufacturer's instructions. The RNA from both bacteria and cells was then reverse‐transcribed into cDNA using the SYBR PrimerScript RT‐PCR Kit (EZBioscience, USA). Finally, RT‐qPCR was performed using SYBR Green Master Mix (EZBioscience, USA) and a LightCycler 480 (Roche, USA) to measure the expression levels of target genes. β‐actin was used as the housekeeping gene for cells, and gyrB for bacteria. The relevant primers are listed in Table S2.

### 4.13 Bacterial Metabolic Function Analysis

Bacteria from each treatment group were collected and analyzed for energy metabolism using glucose assay kits (Beyotime, China), pyruvate assay kits (Solarbio, China), and ATP assay kits (Beyotime, China). Activities of key TCA cycle enzymes, including aconitase (ACO) and succinate dehydrogenase (SDH), were measured using specific activity assay kits (Abbkine, China).

### 4.14 Synergistic Anti‐Biofilm Activity of IMAG and Vancomycin

Mature biofilms were formed in 96‐well plates (1 × 10^6^ CFU mL^−1^, 200 µL per well). After washing to remove planktonic bacteria, biofilms were treated with IMAG and vancomycin either alone or in combination. The MBEC was determined by CFU counting after treatment.

### 4.15 Immunomodulation Ability In Vitro

#### 4.15.1 Immunophenotypes of Macrophages

For the detection of immunophenotype, macrophages (1 × 10^6^ cells per well) were seeded in 6‐well plates and co‐cultured with various nanoparticles for 24 h, with PBS‐treated macrophages serving as controls. Cells were then collected, washed with PBS, and stained with PE‐labeled anti‐CD206 antibody (Bio Legend, USA) and APC‐labeled anti‐CCR7 antibody (Bio Legend, USA). Flow cytometry was performed following resuspension in 500 µL PBS.

For immunofluorescence staining, macrophages from each group were washed with PBS, fixed with an immunostaining fixation solution (Beyotime, China), and blocked with an immunostaining blocking buffer (Beyotime, China). Cells were subsequently incubated with primary antibodies against arginase‐1 (Arg‐1, Beyotime, China) and CD86 (Abcam, USA), followed by corresponding fluorescence‐labeled secondary antibodies. After DAPI staining, samples were observed using a fluorescence microscope.

RT‐qPCR and ELISA were used to evaluate the gene expression and protein secretion of inflammatory factors in macrophages from each treatment group. RT‐qPCR was performed as described above. For ELISA, culture supernatants were collected and centrifuged, and the levels of IL‐1β, IL‐6, and TNF‐α were measured using ELISA kits (BioTNT, China).

#### 4.15.2 Metabolism and Mitochondrial Functions of Macrophages

The metabolic status of macrophages in each group was evaluated using the Seahorse XF96 Extracellular Flux Analyzer (Agilent, USA). Cells (5 × 10^4^ cells per well) were seeded into XF96 microplates and co‐cultured with the respective nanoparticles, as mentioned above. Following the manufacturer's instructions, the Seahorse XF Cell Mito Stress Test Kit (Agilent, USA) and Seahorse XF Glycolytic Rate Assay Kit (Agilent, USA) were used to measure the oxygen consumption rate (OCR) and extracellular acidification rate (ECAR). For OCR measurement, the XF medium was supplemented with 10 mM glucose, 2 mM L‐glutamine, and 1 mM sodium pyruvate. Then, 1 µM oligomycin, 1 µM carbonyl cyanide 4‐(trifluoromethoxy)phenylhydrazone (FCCP), and 1 µM antimycin A/ rotenone were added at specified intervals. For ECAR measurement, 10 mM glucose, 1 µM oligomycin, and 50 mM 2‐deoxyglucose (2‐DG) were sequentially added into the XF medium under basal conditions at different time points. All compounds mentioned above were purchased from Sigma‐Aldrich.

The activities of enzymes related to the tricarboxylic acid (TCA) cycle, including pyruvate dehydrogenase (PDH), aconitase2 (ACO2), and isocitrate dehydrogenase (IDH), as well as the mitochondrial electron transport chain complex I (NADH dehydrogenase) and complex II (succinate‐coenzyme Q reductase), were measured using corresponding activity assay kits (Abbkine, China) following the manufacturer's protocols. For ACO2 activity assessment, cells were pretreated with 0.007% digitonin to remove cytoplasmic isoenzyme ACO1 [68].

To detect changes in mitochondrial membrane potential in macrophages, cells (4 × 10^5^ cells per well) were seeded and incubated with 2 µM JC‐1 dye (Beyotime, China) at 37°C for 20 min. After washing with PBS, the cells were observed under a fluorescence microscope. JC‐1 aggregates in healthy mitochondria emit red fluorescence, whereas a decrease in mitochondrial membrane potential results in JC‐1 monomers emitting green fluorescence. Additionally, TEM was employed to observe the mitochondrial ultrastructure in each group.

#### 4.15.3 Antibacterial Ability of Macrophages In Vitro

PBS‐treated macrophages served as the control. After co‐culturing the macrophages with nanoparticles, the following experiments were conducted to assess the impact of the materials on the antibacterial ability of macrophages. To detect intracellular ROS, macrophages were stained with 10 µM DCFH‐DA and Hoechst 33342 at 37°C, observed under a fluorescence microscope, and subsequently analyzed by flow cytometry. The fluorescence intensity was quantitatively assessed using a fluorescence microplate reader (excitation 488 nm, emission 525 nm).

For the assessment of phagocytic ability, cells (1 × 10^5^ cells per well) were treated with nanoparticles in 24‐well plates. After co‐incubation with *S. aureus ST8‐GFP* (MOI = 10:1) for 1 h, cells were treated with lysostaphin for 15 min and washed with PBS to remove extracellular bacteria. The cells were then fixed, permeabilized, and sequentially stained with Actin‐Tracker Red 555 and DAPI, followed by fluorescence microscopy observation. Flow cytometry was used to analyze the phagocytosis rate, and bacterial uptake was quantified using SPM.

To evaluate the anti‐biofilm ability of macrophages, *S. aureus ST8‐GFP* biofilms were induced with or without IMAG treatment in TSBG, as described previously. Biofilms without IMAG treatment remained relatively intact (“intact biofilm”), whereas those treated with IMAG exhibited disrupted structures (“disrupted biofilm”). Macrophages (1 × 10^5^ cells per well), pretreated with various nanoparticles as described earlier, were stained with 5 µM CellTracker Blue for 30 min and then co‐incubated with either the “intact biofilm” or “disrupted biofilm” at 37°C for 2 h. The invasion and adhesion of macrophages to the biofilms were imaged with CLSM, and residual bacteria in the biofilms after co‐incubation were quantified using SPM, as described previously [93].

### 4.16 Cell Migration Assay

L929 cells (1 × 10^6^ cells per well) were seeded into 6‐well plates and cultured overnight. A sterile 200 µL pipette tip was used to create a straight‐line scratch in the cell monolayer, followed by washing with sterile PBS. PBS was added to the control group, while experimental groups were treated with different nanoparticles. After incubation at 37°C for 0 and 24 h, cells were fixed with methanol and stained with 0.1% crystal violet. Following PBS washes, the stained cells were observed under an optical microscope. Migration rates were analyzed using ImageJ software.

### 4.17 Tube Formation Assay

Before the experiment, a layer of Matrigel matrix (BD Biosciences, USA) was coated on a 24‐well plate. HUVECs (1 × 10^5^ cells per well) were then seeded onto the matrix and co‐cultured with various nanoparticles at 37°C, with PBS‐treated cells as controls. After 8 h of incubation, cells were fixed, permeabilized, and stained with Actin‐Tracker Red 555 (Beyotime, China) for 2 h to visualize the cytoskeleton, followed by DAPI staining for nuclei. Images were captured using a fluorescence microscope, and the number of circles formed by HUVECs was counted.

### 4.18 Animal Ethics Approval

In this study, all animal experiments were approved by the Animal Welfare Ethics Committee of Shanghai Sixth People's Hospital (DWLL2025‐0875). All procedures were conducted following established guidelines.

### 4.19 Diabetic Mouse Model

To induce diabetes, 6‐week‐old male BALB/c mice were intraperitoneally injected with 60 mg/kg streptozotocin (STZ) (Sigma, USA) for 5 consecutive days. The diabetes model was considered established if blood glucose levels were ≥16.7 mmol/L two weeks after injection.

### 4.20 Treatment of Implant‐Associated Infections in Diabetes In Vivo

#### 4.20.1 Diabetic Mouse Implant‐Associated Infection Model

After anesthetizing the diabetic mice, their back fur was shaved and disinfected. A 1 cm incision was made on the back, and a sterile titanium plate (d = 1 cm) was implanted subcutaneously before the incision was sutured. Subsequently, 50 µL of *S. aureus ST8* suspension (1 × 10^7^ CFU mL^−1^) was injected onto the surface of the implant. For macrophage depletion experiments, mice received an intraperitoneal injection of clodronate liposomes (200 µL per mouse, MCE, USA) 48 h prior to infection, and re‐injection every 3 days until sacrifice.

#### 4.20.2 Pharmacokinetic and Targeting Capability of IMAG In Vivo

The infected mice were randomly divided into three groups, following intravenous injection with 200 µL Cy5.5‐labeled MSN@Arg@GOx, UMAG, or IMAG solutions (500 µg mL^−1^) on day 1 postinfection, respectively. Blood samples were collected at predetermined time points (0.25, 0.5, 1, 2, 4, 8, 24, and 48 h) to determine nanoparticle pharmacokinetics by measuring plasma fluorescence intensity. Subsequently, fluorescence images were captured at 0, 4, 8, 16, 24, and 48 h postinjection. At 48 h, major organs were harvested for imaging and biodistribution analyses.

### 4.21 Microbiological Assessment

To continuously monitor the infection process, another batch of diabetic mice underwent titanium plate implantation and incision suturing, followed by injection of 50 µL of *S. aureus ST8‐lux* suspension (1 × 10^7^ CFU mL^−1^) onto the surface of the implants. Mice were then randomly assigned to five groups. On days 0 and 7 postinfection, the control group received 200 µL of PBS intravenously, while the experimental groups were treated with various nanoparticles intravenously (500 µg mL^−1^). Bioluminescent images of the infection sites were captured on days 0, 4, 7, 10, and 14, and the infection progression was observed.

On day 14 postinfection, the implants and surrounding soft tissues were collected, and bacterial load was determined using SPM. Additionally, implants from day 14 were fixed, dehydrated, freeze‐dried, coated with platinum, and examined by SEM.

### 4.22 Immunological Evaluation and Histological Analysis

Mice were euthanized at various time points to collect infected tissues for subsequent experiments. On day 4 postinfection, infected tissues were prepared as frozen sections for ROS staining. Tissues harvested from days 4 and 14 were fixed in 4% paraformaldehyde, dehydrated through an ethanol gradient, embedded in paraffin, and sliced into sections. Sections were then subjected to Giemsa staining, H&E staining, and Masson staining to determine the status of infection, inflammation, and collagen formation, respectively. Furthermore, slices were stained with primary antibodies against iNOS (Abcam, USA) and CD206 (Abcam, USA), along with corresponding secondary antibodies, to assess immune cell infiltration and subtype changes over time, while CD31 (Abcam, USA) and α‐SMA (Abcam, USA) were used to evaluate angiogenesis. All the sections were visualized using optical or fluorescence microscopy. Finally, ELISA was employed to measure the levels of inflammatory cytokines in the surrounding tissues of the implants on days 4 and 14 post‐infection.

### 4.23 Biofilm‐Infected Wound Healing Capability in Diabetic Mice In Vivo

#### 4.23.1 Diabetic Mouse Biofilm‐Related Wound Infection Model

The model was constructed as described before [81]. The STZ‐induced diabetic BALB/c mice were anesthetized, and their dorsal skin was shaved and disinfected. A full‐thickness circular skin wound (15 mm) was created. *S. aureus ST8* biofilms were prepared on PCL fiber membranes, which were then applied to the wounds. The membranes were removed after 2 h, leaving the biofilms on the wounds. Mice were randomly assigned to five groups. Different groups of nanoparticles (500 µg mL^−1^) were applied to the wounds at 0 and 7 days postoperatively, with an equal volume of sterile PBS used as the control.

#### 4.23.2 Microbiological Assessment, Immunological Evaluation, and Histological Analysis

The progression of wound healing was evaluated on days 0, 1, 4, 7, 10, and 14 postsurgery. SPM was performed to assess bacterial load using infected tissues collected on days 4 and 14. Frozen sections for ROS staining were prepared from fresh wound tissues collected on day 4. ELISA was conducted on tissues from days 4 and 14 to measure inflammatory factors. Additionally, tissues collected on days 4, 10, and 14 postsurgery were fixed, dehydrated, embedded in paraffin, and sectioned into 4 µm slices. Giemsa staining was used to assess bacterial infiltration, while H&E staining was employed for both qualitative evaluation of tissue inflammation and quantitative measurement of wound healing indicators, including epidermal thickness on days 4, 10, and 14, hair follicle count, and dermal thickness on day 14. Collagen deposition was assessed via Masson staining. Immunofluorescence staining with antibodies against iNOS (Abcam, USA), CD206 (Abcam, USA), VEGF (Abcam, USA), α‐SMA (Abcam, USA), and CD31 (Abcam, USA) was conducted on slices to evaluate macrophage phenotype changes and angiogenesis. All slices were observed under optical or fluorescence microscopy, and immunofluorescence intensity was quantified using ImageJ software. 3‐NT was evaluated on day 14 after the surgery for potential nitrosative stress. For in vivo biocompatibility assessment, serum biochemistry tests were performed on day 14 after the surgery, and major organs were collected for H&E staining. In addition, coagulation parameters and serum complement levels (C3a, C5a) were measured to evaluate the interaction of the nanoparticles with blood components and to preliminarily assess hemocompatibility and immunocompatibility.

### 4.24 Statistical Analysis

Statistical analyses were conducted using GraphPad Prism 8 software. Results were expressed as mean ± standard deviation (SD). Statistical significance among multiple groups was analyzed using one‐way ANOVA followed by Tukey's multiple comparisons test, with a significance threshold of *p* < 0.05. Noteworthy labels and annotations were added to ensure clarity in the study.

## Author Contributions

**Mingzhang Li**: conceptualization, methodology, data curation, writing – original draft, writing – review and editing, investigation, validation, formal analysis, visualization. **Changming Wang**: investigation, validation, visualization. **Feng Jiang**: methodology, validation, visualization. **Jiren Yan**: data curation, formal analysis, methodology, investigation. **Jinlong Yu**: validation, data curation, methodology, investigation. **Hao Shen**: funding acquisition, writing – review and editing, conceptualization, investigation, data curation, project administration. **Boyong Wang**: investigation, formal analysis, methodology. **Yi Yang**: investigation, visualization, formal analysis, data curation. **Botao Song**: conceptualization, writing – original draft, writing – review and editing, validation, data curation. **Pei Han**: investigation, data curation, validation, methodology. **Deyi Ding**: data curation, validation, investigation. **Jin Tang**: investigation, validation. **Geyong Guo**: investigation, validation, methodology, writing – review and editing, visualization, funding acquisition. **Yonglong Li**: investigation, methodology, writing – original draft, data curation.

## Conflicts of Interest

The authors declare no conflicts of interest.

## Supporting information




**Supporting File**: advs76411‐sup‐0001‐SuppMat.docx.

## Data Availability

The data that support the findings of this study are available from the corresponding author upon reasonable request.
