# Supplementary material for: Biomimetic Nanoplatform for Dual Target Nano‐Metabolic Therapy in Diabetes‐Associated Biofilm Infections
Source: Adv Sci (Weinh). 2026 Jul 3:e76411. Online ahead of print. doi: 10.1002/advs.76411 (PMC13334585; doi:10.1002/advs.76411)
Supplement: Supplementary file 1 — Supporting File: advs76411‐sup‐0001‐SuppMat.docx. [file ADVS-9999-e76411-s001.docx]

**Biomimetic Nanoplatform for Dual-target Nano-metabolic Therapy in Diabetes-associated Biofilm Infections**

Mingzhang Li^1^, Yonglong Li^2^, Jiren Yan^1^, Changming Wang^1^, Jinlong Yu^1^, Feng Jiang^1^, Boyong Wang^1^, Yi Yang^1^, Deyi Ding^1^, Jin Tang^3^, Pei Han^1^, Botao Song^2^*, Geyong Guo^1^*, Hao Shen^1^*

M. Li, J. Yan, C. Wang, J. Yu, F. Jiang, B. Wang, Y. Yang, D. Ding, P. Han, G. Guo, H. Shen

1 Department of Orthopedics, Shanghai Sixth People’s Hospital Affiliated to Shanghai Jiao Tong University School of Medicine, Shanghai Jiao Tong University, Shanghai 200233, P. R. China.

Y. Li, B. Song

2 Key Laboratory of Synthetic and Natural Functional Molecule of the Ministry of Education, College of Chemistry and Materials Science, Northwest University, Xi'an 710069, P. R. China.

J. Tang

3 Department of Clinical Laboratory, Shanghai Sixth People's Hospital Affiliated to Shanghai Jiao Tong University School of Medicine, Shanghai Jiao Tong University, Shanghai 200233, P. R. China.

Corresponding Author:

Botao Song (ORCID: 0000-0002-0563-7196, Email: [botaosong@nwu.edu.cn](mailto:botaosong@nwu.edu.cn))

Geyong Guo (ORCID: 0000-0002-8536-5062, Email: guoyou2014@alumni.sjtu.edu.cn)

Hao Shen (ORCID: 0009-0004-6766-6066, Email: [shenhao7212@shsmu.edu.cn](mailto:shenhao7212@shsmu.edu.cn))

**Supplementary Figures**

**
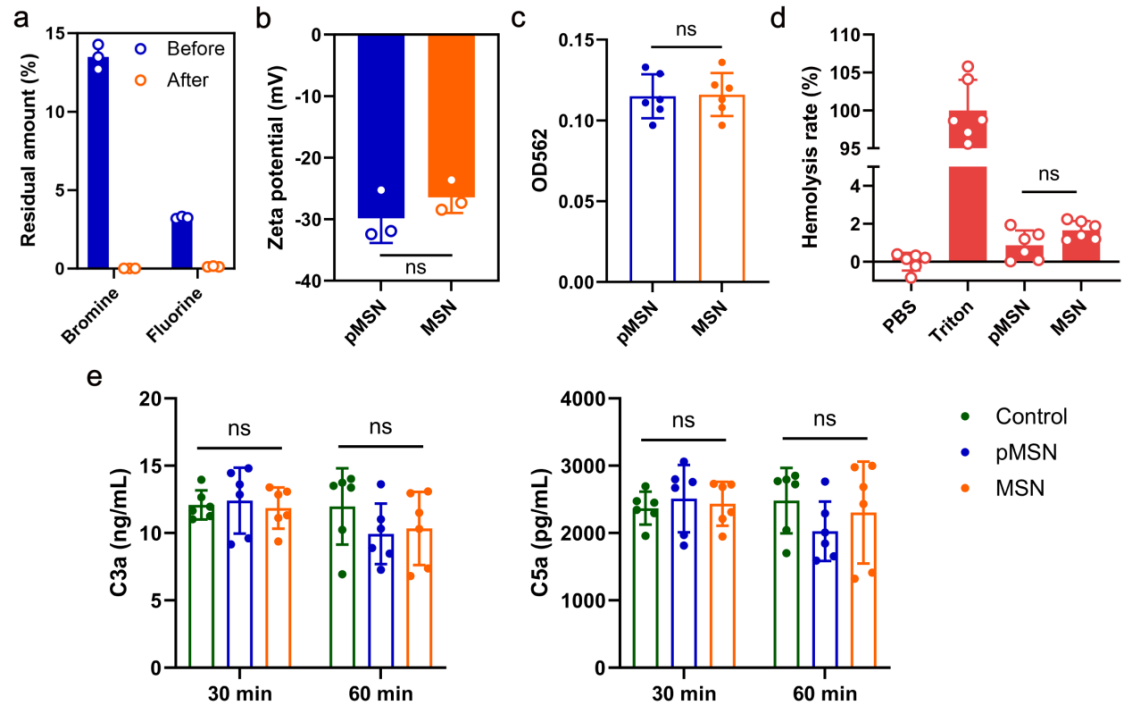
**

**Supplementary Fig. 1. Residual ions, surface charge, and biocompatibility evaluation of nanoparticles. a,** fluoride and bromide levels before and after calcination (n=3). **b,** Zeta potential of pMSNs and amino-functionalized MSNs (MSNs) (n=3). **c,** Protein-corona formation of pMSNs and MSNs after incubation with mouse serum (n=6). **d,** Hemolysis ratio of pMSNs and MSNs (n=6). **e,** Complement activation levels (C3a and C5a) in serum following exposure to pMSNs and MSNs (n=6). Data are presented as mean ± SD. ns: no significant. Statistical significance was determined by one-way ANOVA.

**
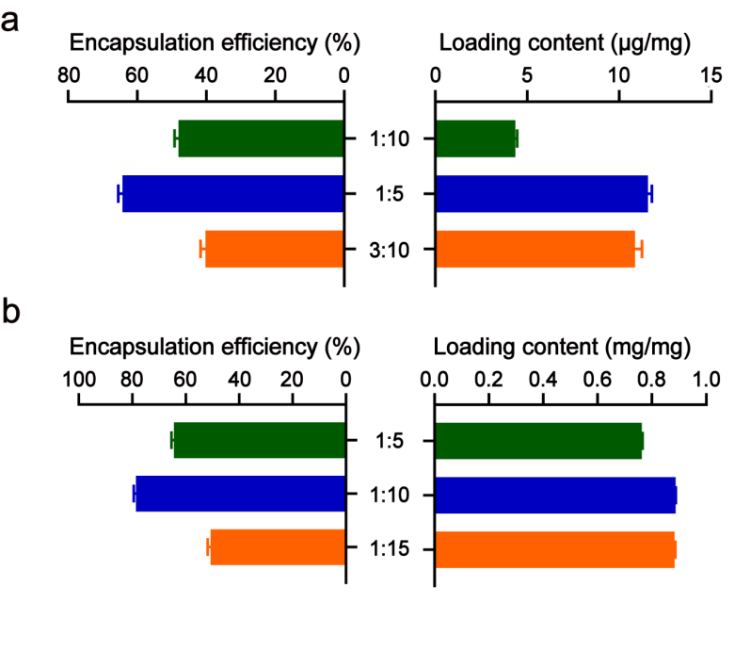
**

**Supplementary Fig. 2. Encapsulation efficiency and loading content of GOx (a) and Arg (b) at different feeding ratios to the carrier (n=3).** Data are presented as mean ± SD.

**
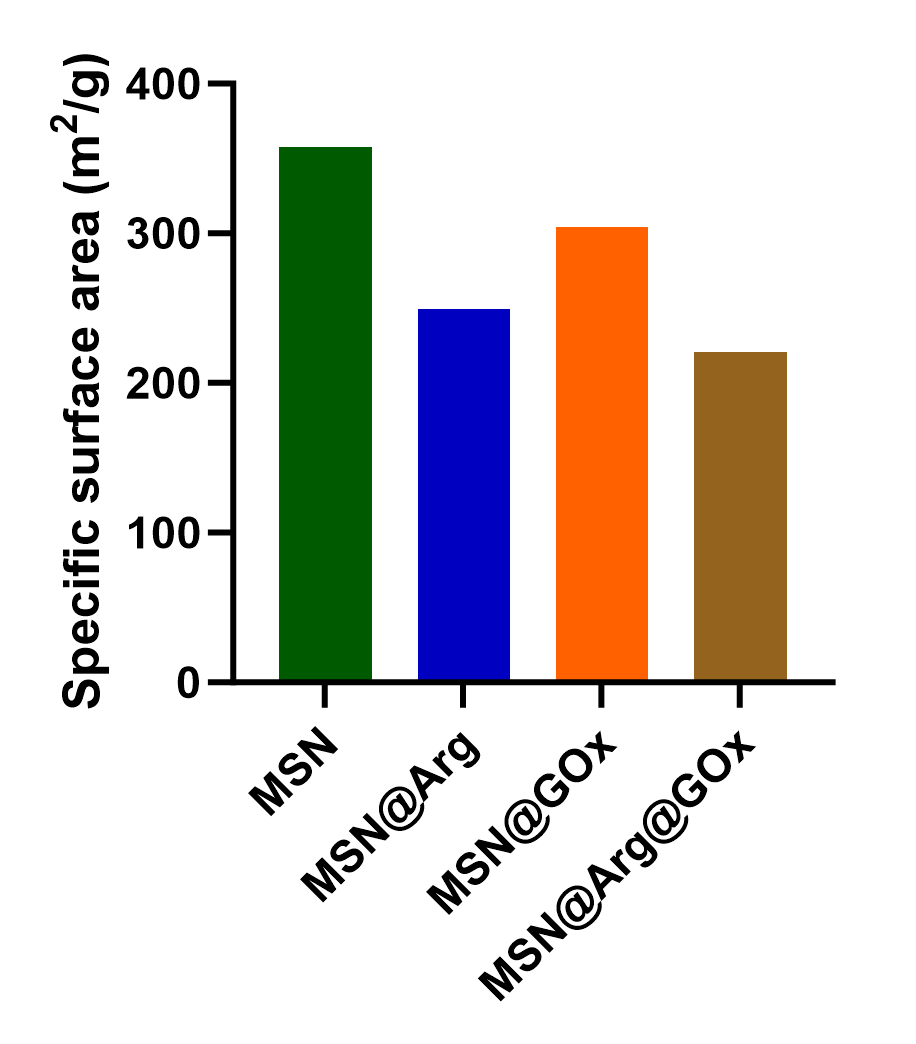
**

**Supplementary Fig. 3. Specific surface areas of different groups of nanoparticles.**

**
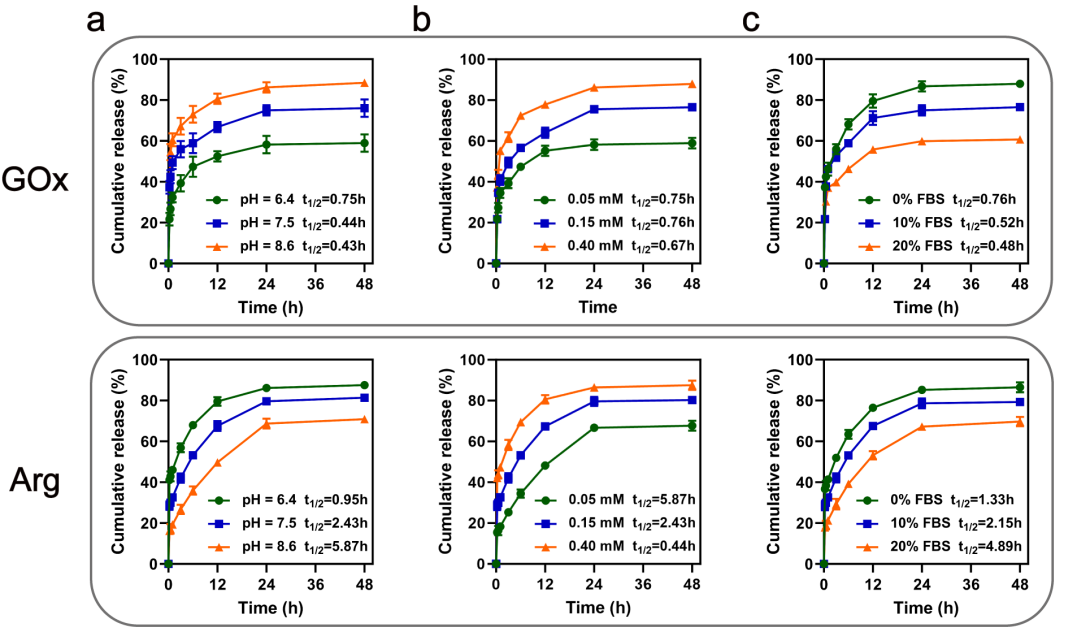
**

**Supplementary Fig. 4. Release profiles and half-lives of GOx and Arg from MSN@Arg@GOx under different conditions (n=3). a,** Release at pH 6.4, 7.5, and 8.6 with constant ionic strength (0.15 mM NaCl) and 10% FBS. **b,** Release at ionic strengths of 0.05, 0.15, and 0.4 mM NaCl at pH 7.5 and 10% FBS. **c,** Release at 0%, 10%, and 20% FBS at pH 7.5 and ionic strength of 0.15 mM NaCl. Data are presented as mean ± SD.

**
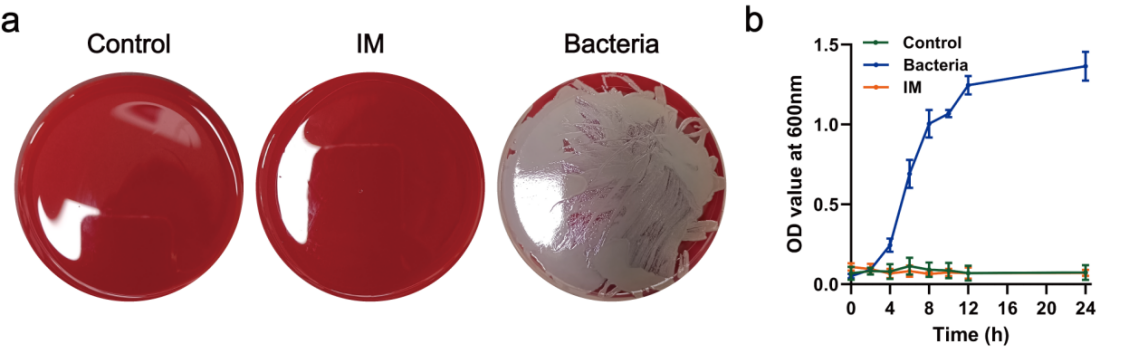
**

**Supplementary Fig. 5. Sterility and bioburden assessment of the nanoparticle. a,** Representative images of bacterial colonies on blood agar for PBS control, membrane-coated MSN (IM), and *S. aureus* positive control. **b,** Growth curves of *S. aureus* in different groups (n=6). Data are presented as mean ± SD.

**
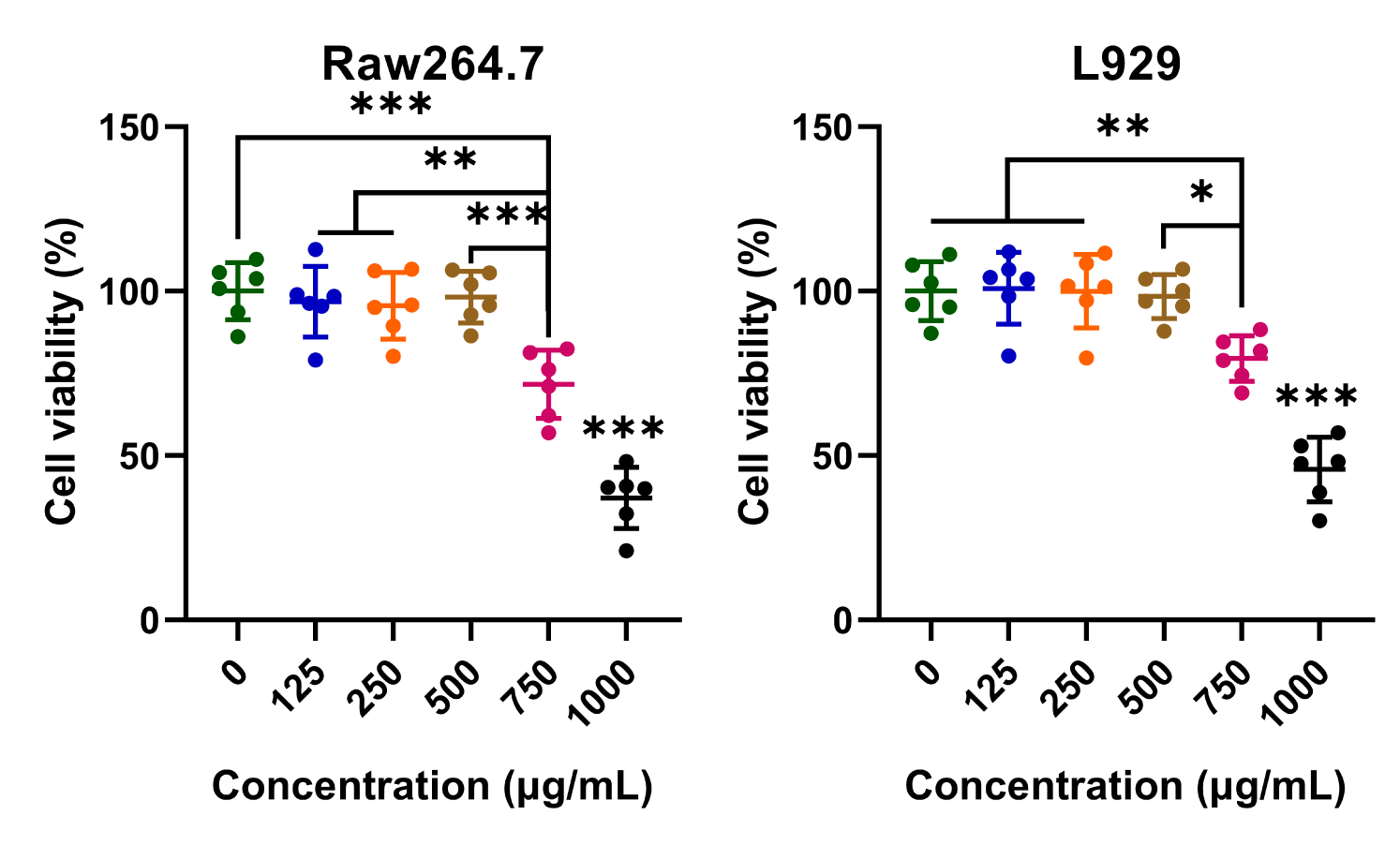
**

**Supplementary Fig. 6. Cell viability of RAW264.7 macrophages and L929 fibroblasts cultured in different concentrations of IMAG (n=6).** Note: ^*^*p* < 0.05, ^**^*p* < 0.01 and ^***^*p* < 0.001. Data are presented as mean ± SD. Statistical significance was determined by one-way ANOVA.


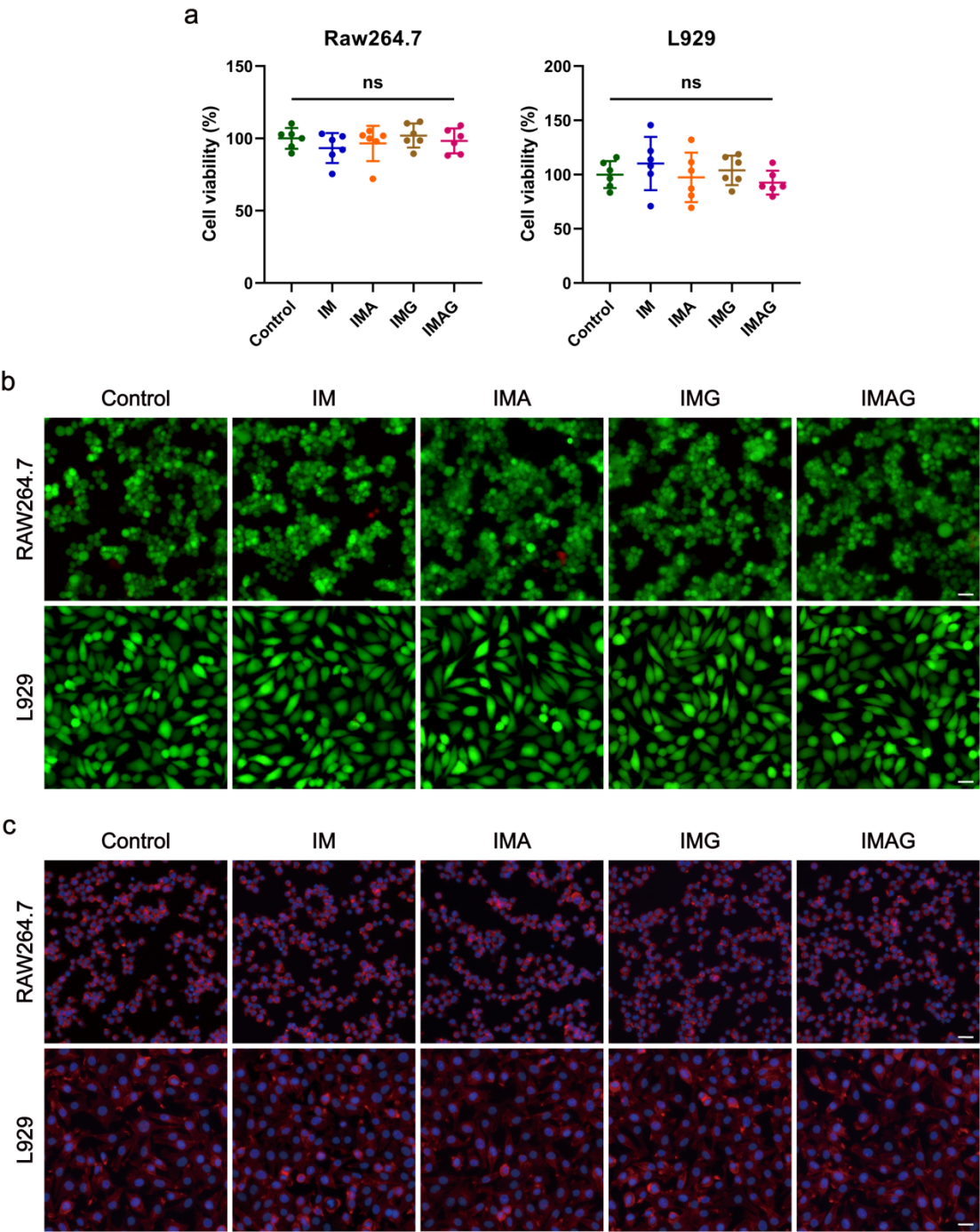


**Supplementary Fig. 7. Cellular biocompatibility of nanoparticles. a,** Cytotoxicity of different treatments on RAW264.7 macrophages and L929 fibroblasts (n=6). ns: no significant. Data are presented as mean ± SD. Statistical significance was determined by one-way ANOVA. **b,** Representative live/dead staining images of macrophages and fibroblasts after different treatments, with live cells emitting green fluorescence and dead cells emitting red fluorescence. Scale bar, 25 μm. **c,** Representative immunofluorescence images of macrophage and fibroblast cytoskeletons (red) and nuclei (blue) after different treatments. Scale bar, 25 μm.


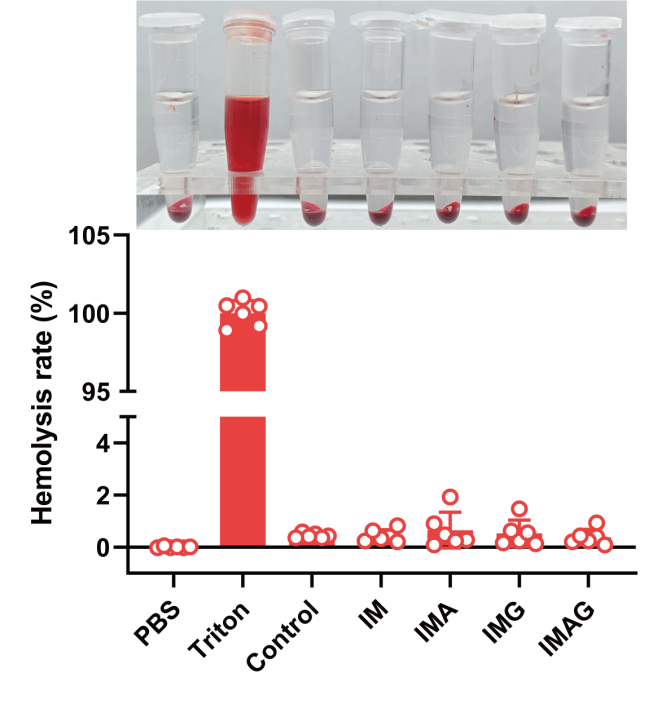


**Supplementary Fig. 8. Hemolysis images of different groups of nanoparticles, along with corresponding hemolysis rates (n=6).** PBS treatment serves as the negative control, and Triton treatment serves as the positive control.


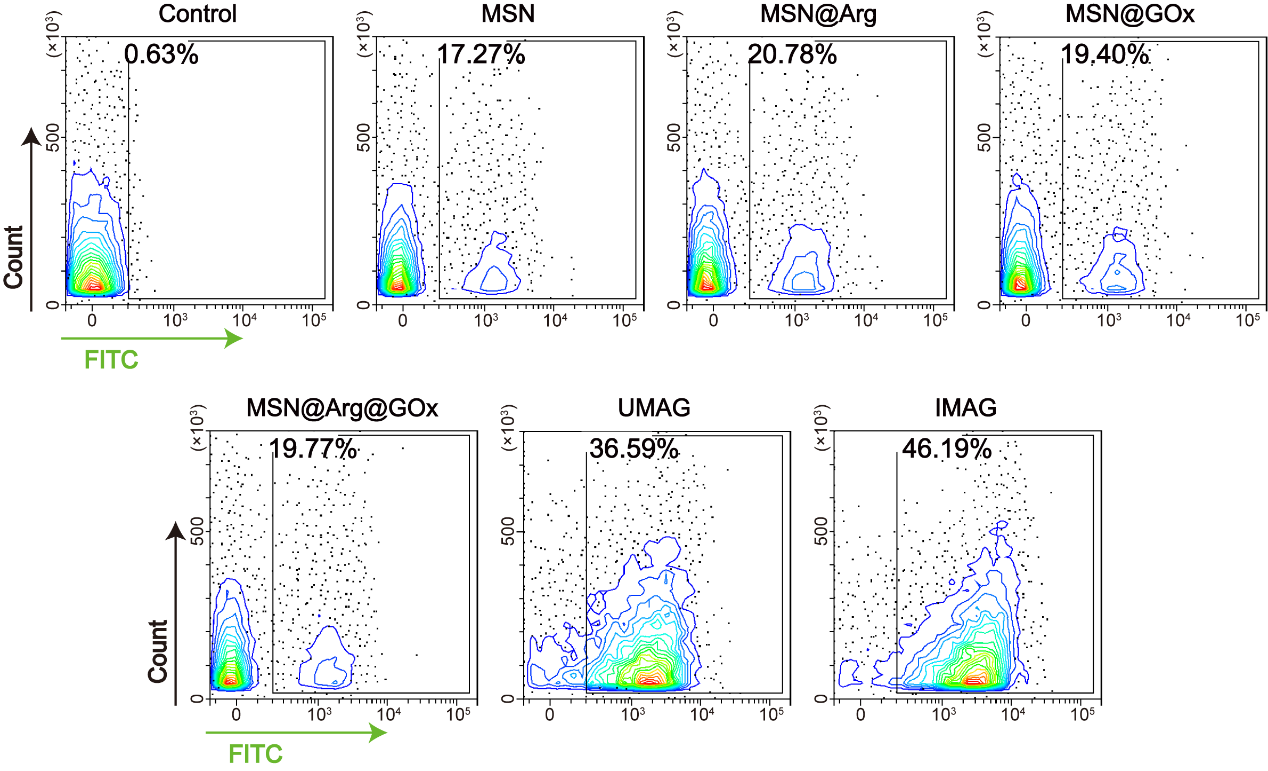


**Supplementary Fig. 9. Analysis of *S. aureus* adhesion to various nanoparticles by flow cytometry.**


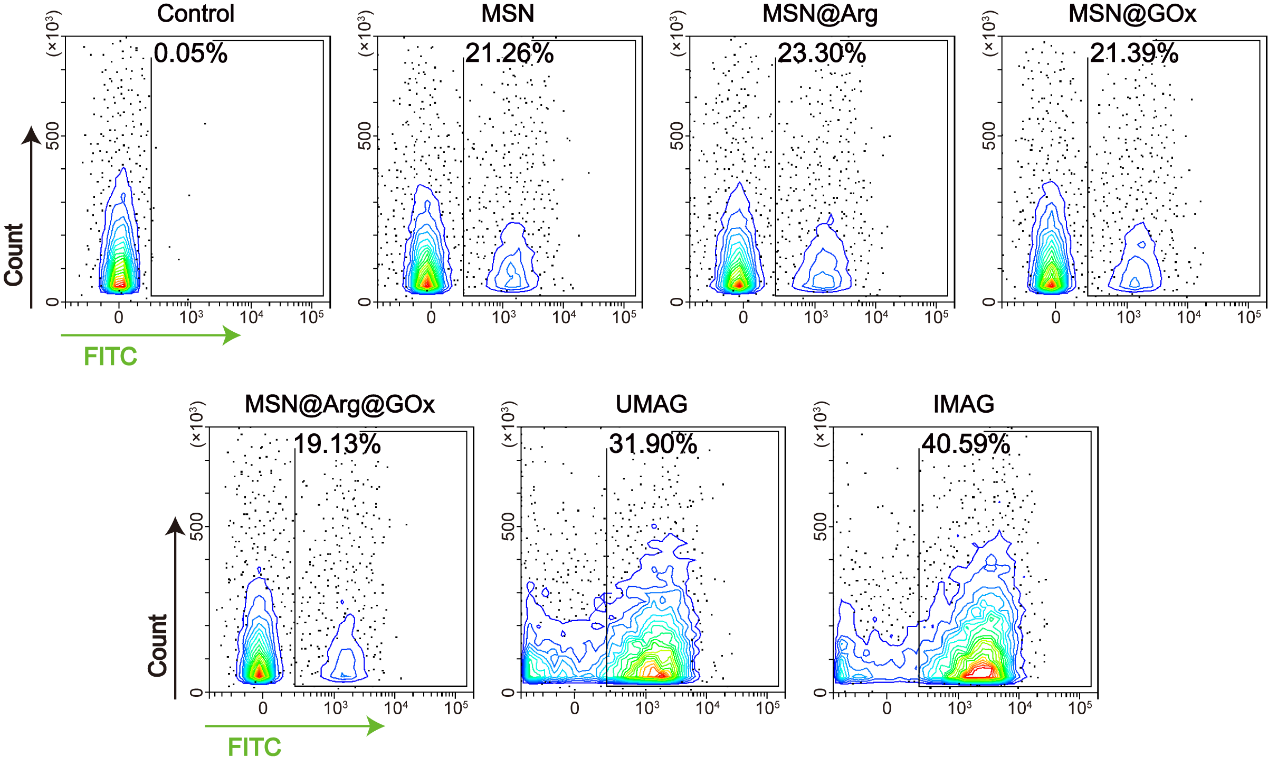


**Supplementary Fig. 10. Analysis of *E. coli* adhesion to various nanoparticles by flow cytometry.**

**
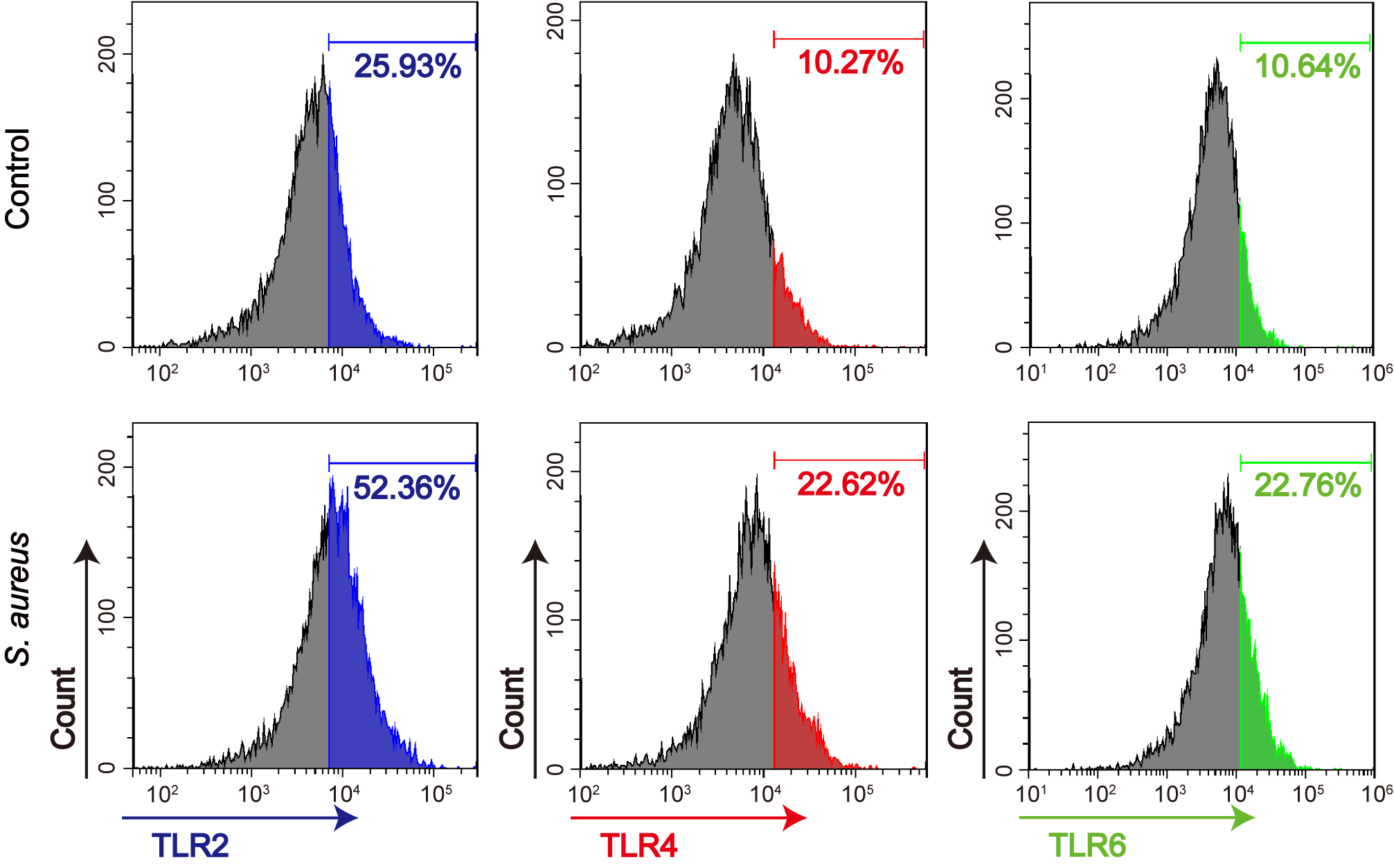
**

**Supplementary Fig. 11. Flow cytometry analysis of TLR expression on macrophage membranes before and after *S. aureus* infection.**

**
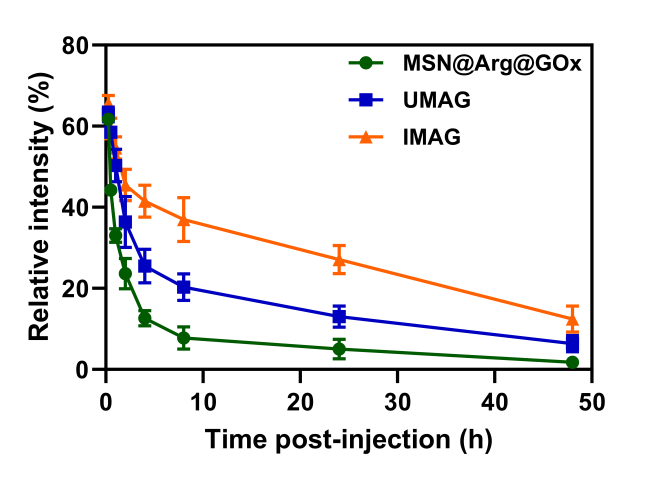
**

**Supplementary Fig. 12. Pharmacokinetic profiles of MSN@Arg@GOx, UMAG, and IMAG in infected mice (n=3).** Data are presented as mean ± SD.

**
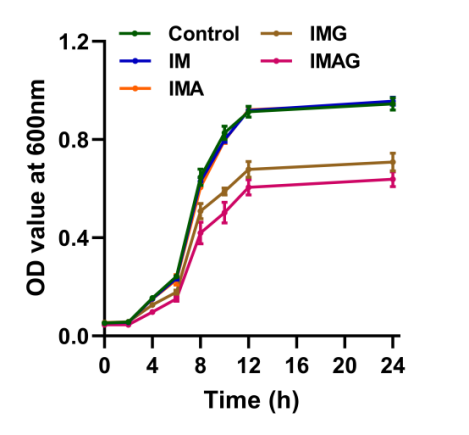
**

**Supplementary Fig. 13.** **Growth curves of** ***E. coli* after different treatments (n=6).** Data are presented as mean ± SD.

**
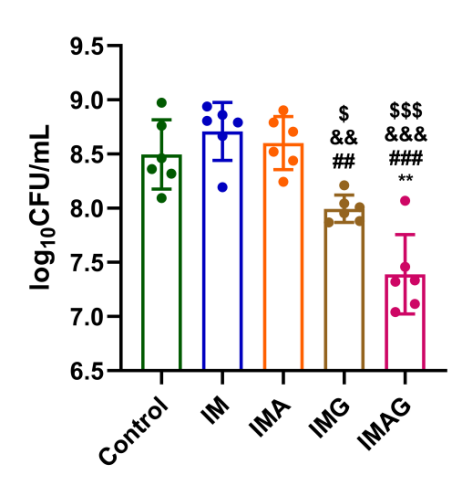
**

**Supplementary Fig. 14. CFU counting results of *E. coli* in each group (n=6).** Note: ^$^*p* < 0.05 and ^$$$^*p* < 0.001 versus the control group; ^&&^*p* < 0.01 and ^&&&^*p* < 0.001 versus IM group; ^##^*p* < 0.01 and ^###^*p* < 0.001 versus IMA group; ^**^*p* < 0.01 versus IMG group. Data are presented as mean ± SD. Statistical significance was determined by one-way ANOVA.

**
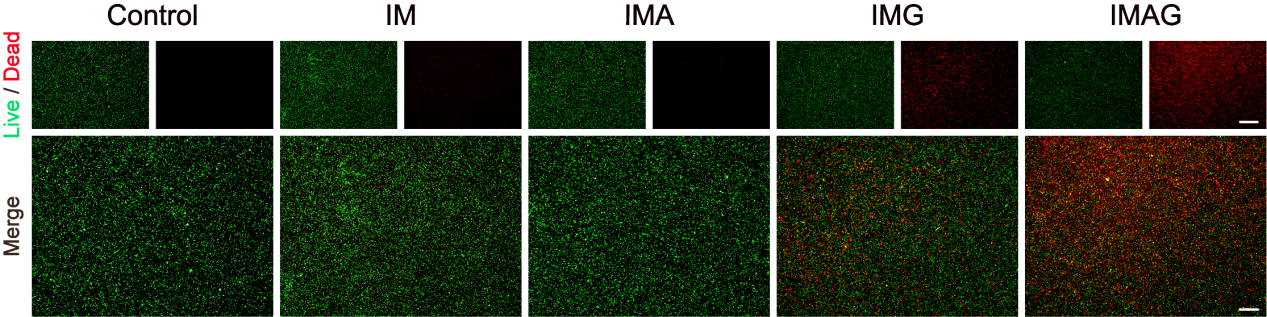
**

**Supplementary Fig. 15. Representative live/dead staining images of *E. coli*, with live bacteria emitting green fluorescence and dead bacteria emitting red fluorescence.** Scale bars, 200 μm and 100 μm.

**
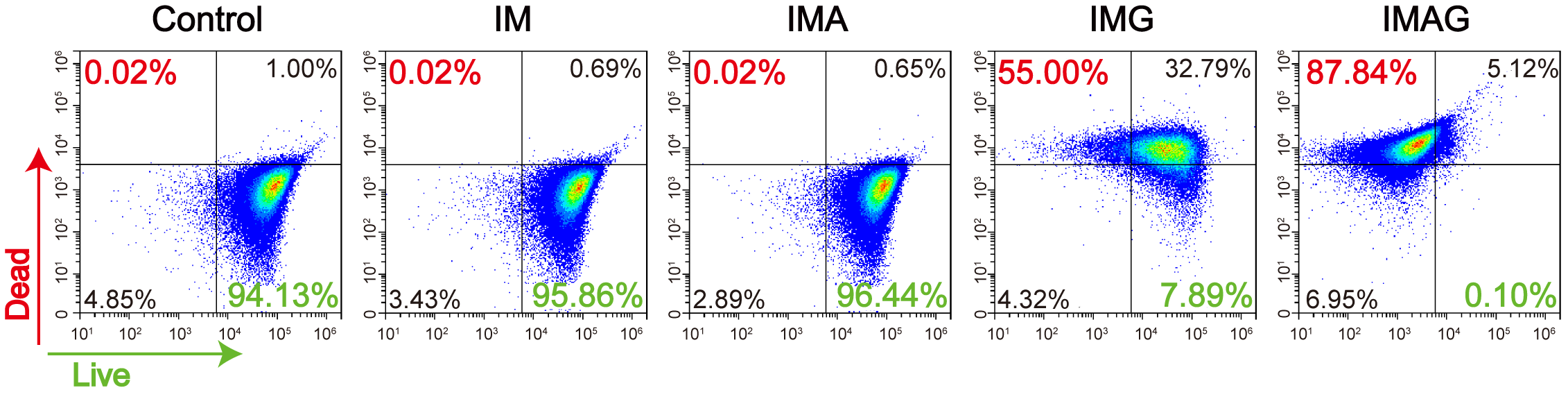
**

**Supplementary Fig. 16. Live/dead flow cytometry results for *E. coli*.**

**
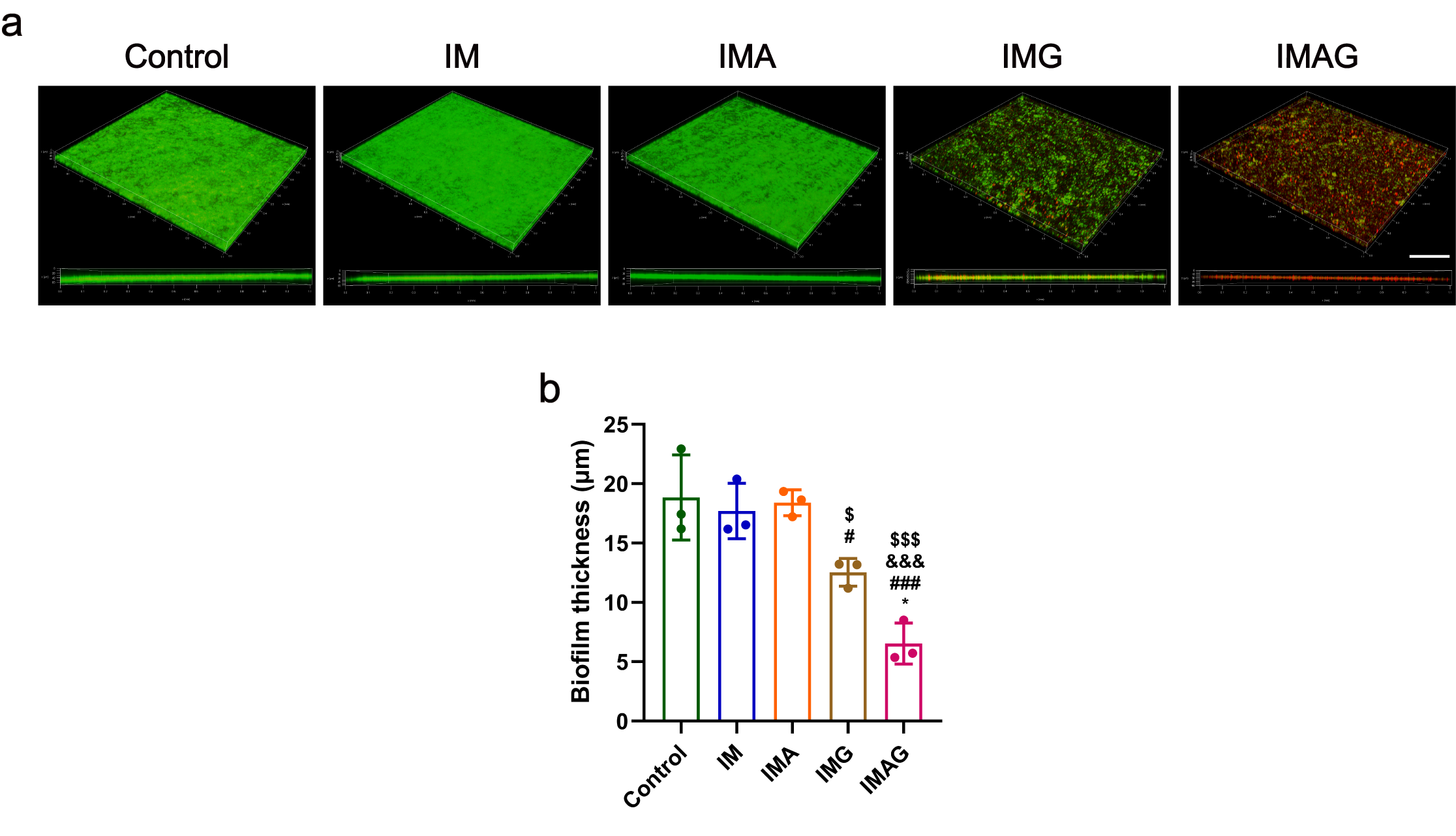
**

**Supplementary Fig. 17. a,** Live/dead staining images of *E. coli* biofilm using CLSM. Scale bar, 200 μm. **b,** Thickness of *E. coli* biofilms (n=3). Note: ^$^*p* < 0.05 and ^$$$^*p* < 0.001 versus the control group; ^&&&^*p* < 0.001 versus IM group; ^#^*p* < 0.05 and ^###^*p* < 0.001 versus IMA group; ^*^*p* < 0.05 versus IMG group. Data are presented as mean ± SD. Statistical significance was determined by one-way ANOVA.

**
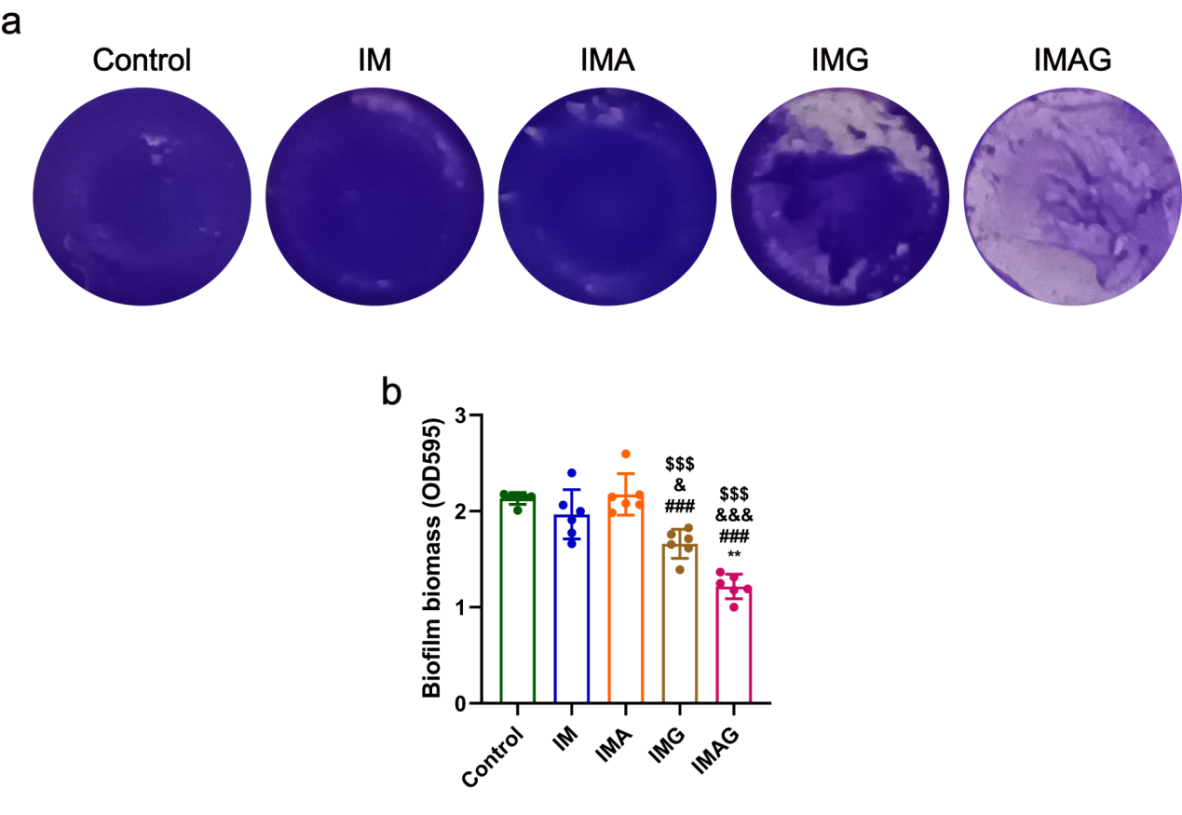
**

**Supplementary Fig. 18. Qualitative and quantitative results of crystal violet staining of *E. coli* biofilm (n=6).** Note: ^$$$^*p* < 0.001 versus the control group; ^&&&^*p* < 0.001 versus IM group; ^###^*p* < 0.001 versus IMA group; ^**^*p* < 0.01 versus IMG group. Data are presented as mean ± SD. Statistical significance was determined by one-way ANOVA.

**
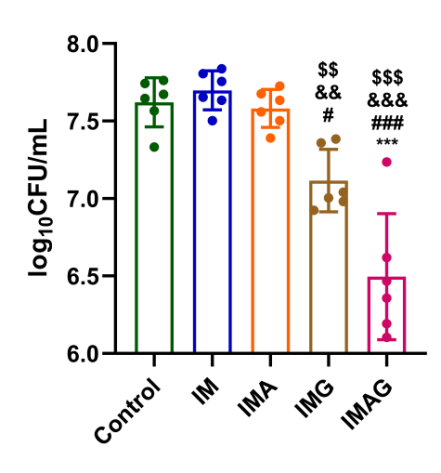
**

**Supplementary Fig. 19. Bacterial load within *E. coli* biofilms after different treatments (n=6).** Note: ^$$^*p* < 0.01 and ^$$$^*p* < 0.001 versus the control group; ^&&^*p* < 0.01 and ^&&&^*p* < 0.001 versus IM group; ^#^*p* < 0.05 and ^###^*p* < 0.001 versus IMA group; ^***^*p* < 0.001 versus IMG group. Data are presented as mean ± SD. Statistical significance was determined by one-way ANOVA.

**
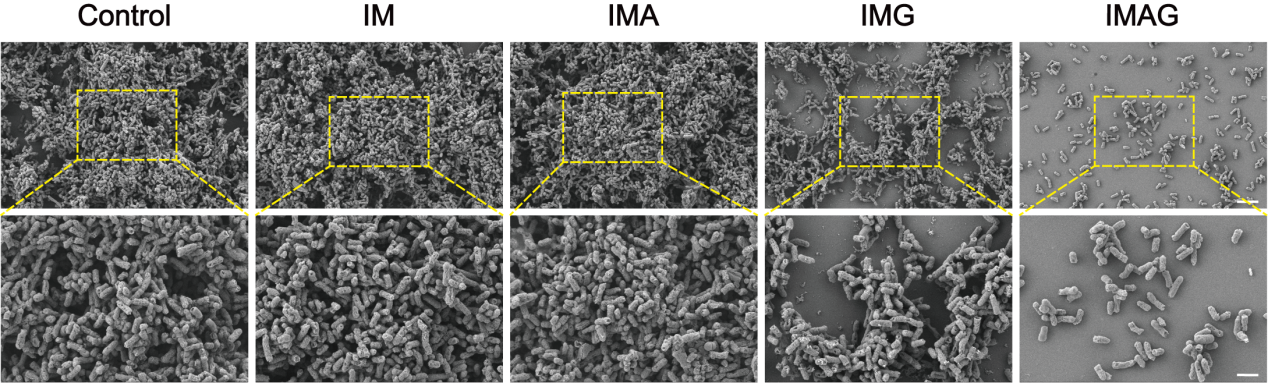
**

**Supplementary Fig. 20. SEM images of the *E. coli* biofilms in each treatment group. Scale bars, 5 μm and 2 μm.**

**
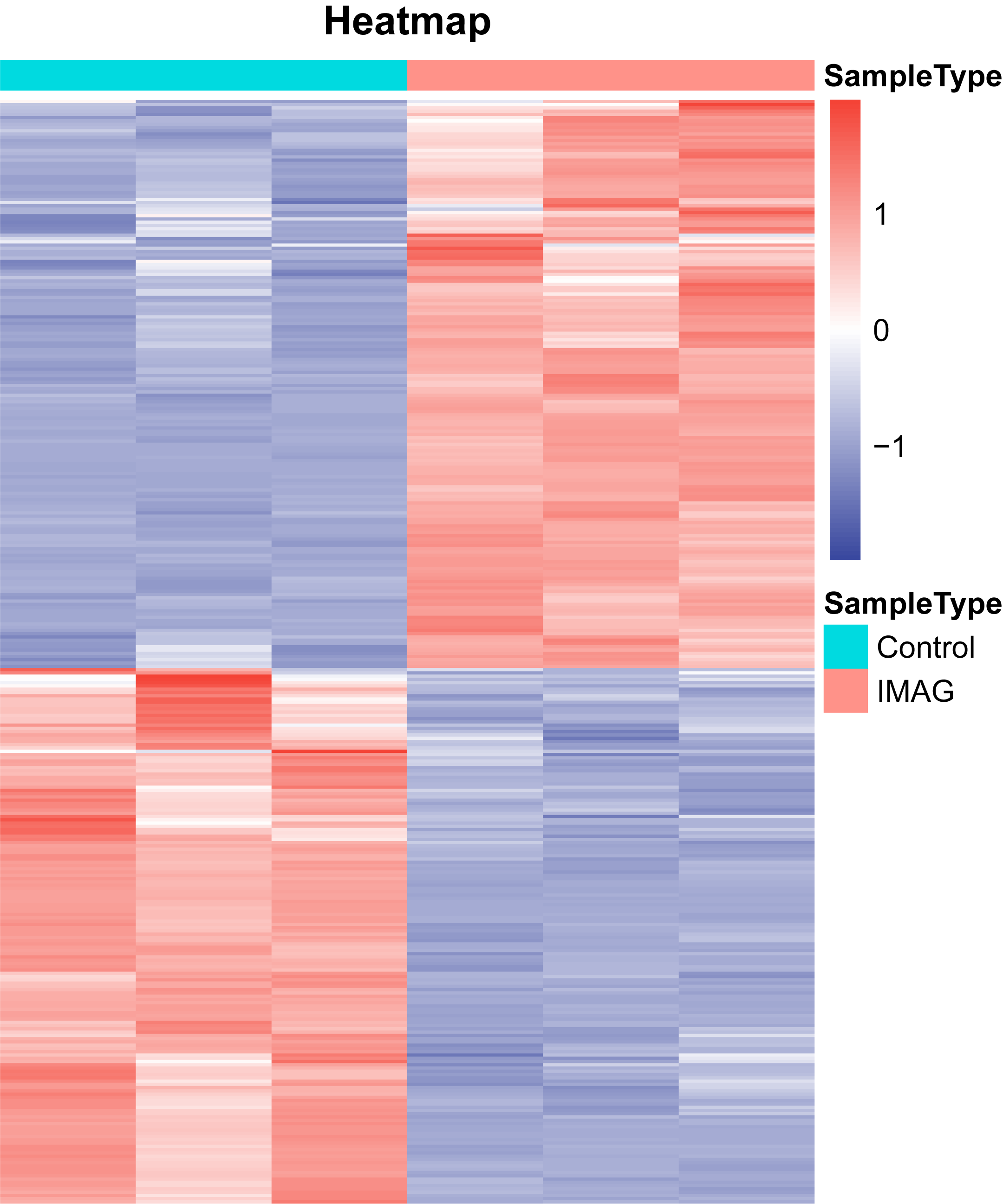
**

**Supplementary Fig. 21. Heatmap of DEGs in IMAG-treated and untreated *S. aureus*.**

**
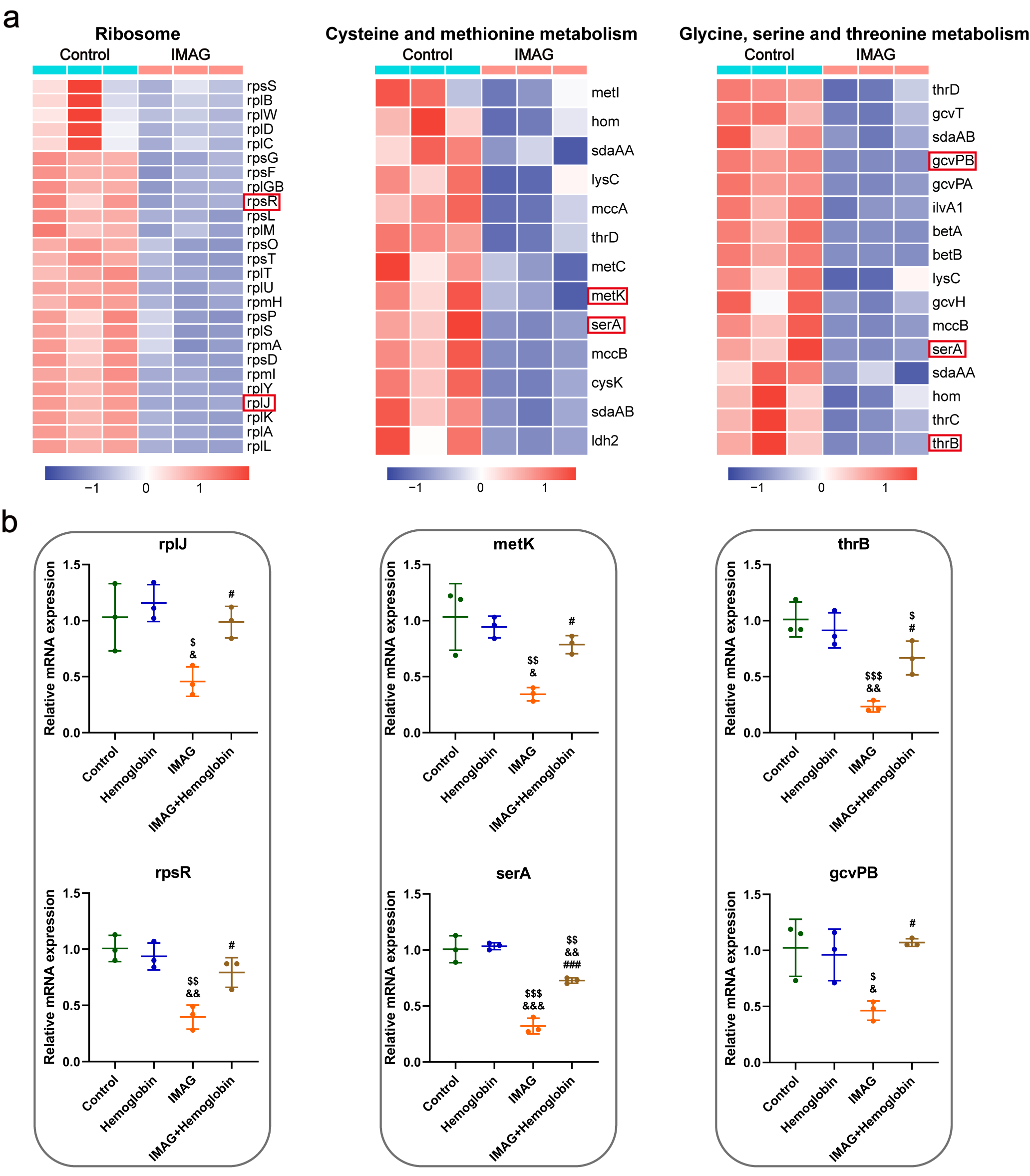
**

**Supplementary Fig. 22. a,** Heatmap of genes involved in the ribosome pathway, cysteine and methionine metabolism pathway, and glycine, serine, and threonine metabolism pathway identified by KEGG enrichment analysis. **b,** qPCR validation of representative genes from the ribosome pathway (rplJ and rpsR), cysteine and methionine metabolism (metK and serA), and glycine, serine, and threonine metabolism (thrB and gcvPB) (n=3). Note: ^$^*p* < 0.05, ^$$^*p* < 0.01 and ^$$$^*p* < 0.001 versus the control group; ^&^*p* < 0.05, ^&&^*p* < 0.01 and ^&&&^*p* < 0.001 versus hemoglobin group; ^#^*p* < 0.05 and ^###^*p* < 0.001 versus IMAG group. Data are presented as mean ± SD. Statistical significance was determined by one-way ANOVA.

**
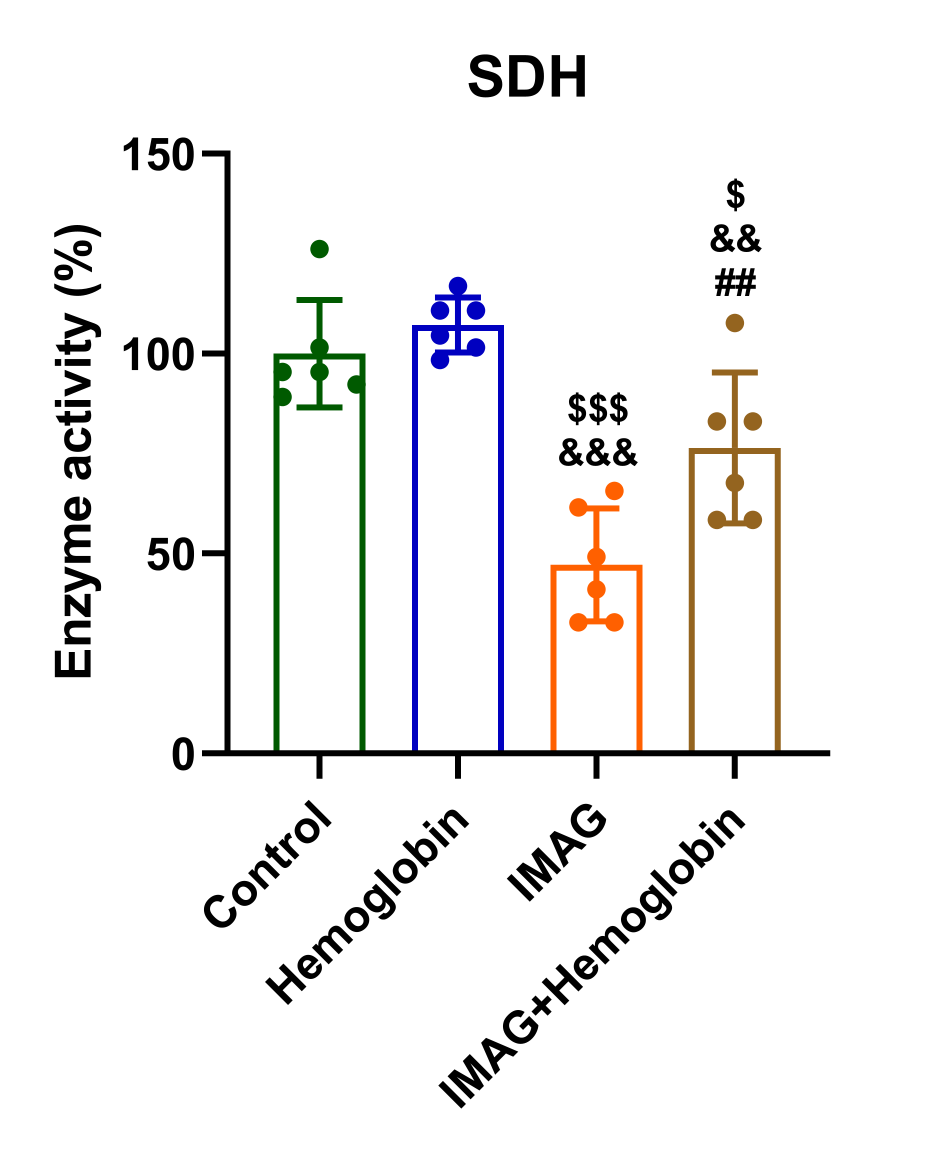
**

**Supplementary Fig. 23. Activity of SDH in *S. aureus* (n=6).** Note: ^$^*p* < 0.05 and ^$$$^*p* < 0.001 versus the control group; ^&&^*p* < 0.01 and ^&&&^*p* < 0.001 versus hemoglobin group; ^##^*p* < 0.01 versus IMAG group. Data are presented as mean ± SD. Statistical significance was determined by one-way ANOVA.


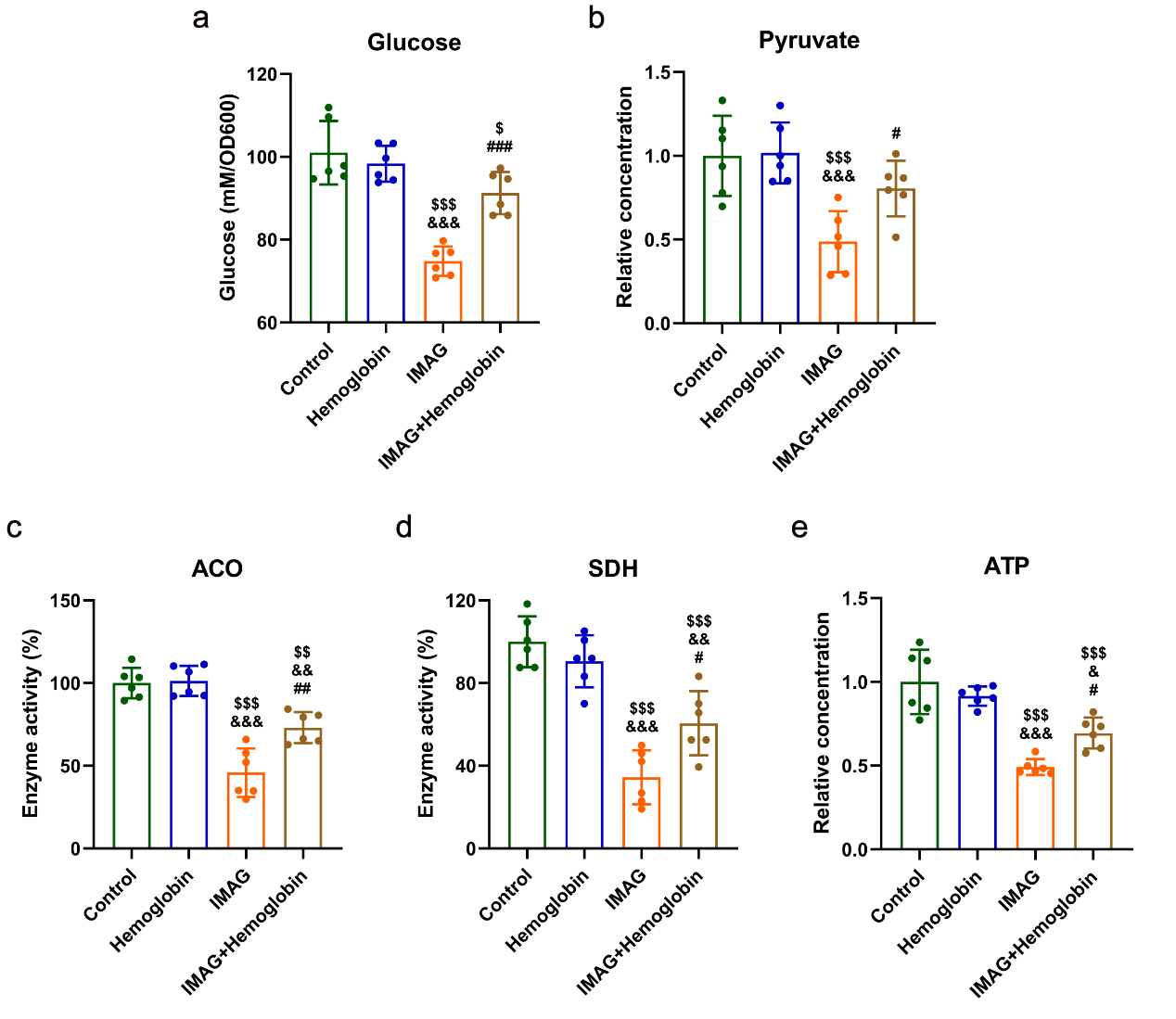


**Supplementary Fig. 24. IMAG interference with *E. coli* metabolism *in vitro*  (n=6)*.* a,** Glucose uptake of *E. coli* in different treatment groups. **b,** Relative content of pyruvate in *E. coli*. **c,** ACO activity of *E. coli* treated by each group. **d,** Activity of SDH in *E. coli*. **e,** Relative concentration of ATP in *E. coli*. Note: ^$^*p* < 0.05, ^$$^*p* < 0.01 and ^$$$^*p* < 0.001 versus the control group; ^&^*p* < 0.05, ^&&^*p* < 0.01 and ^&&&^*p* < 0.001 versus hemoglobin group; ^#^*p* < 0.05, ^##^*p* < 0.01 and ^###^*p* < 0.001 versus IMAG group. Data are presented as mean ± SD. Statistical significance was determined by one-way ANOVA.

**
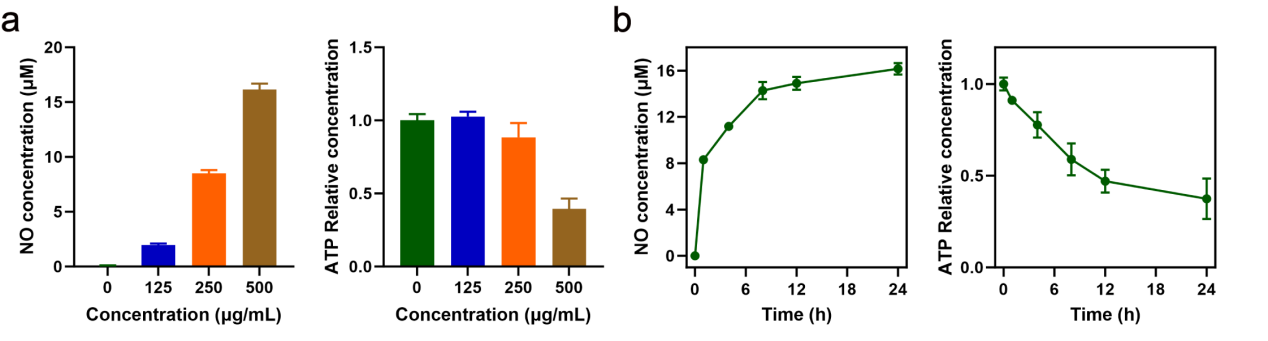
**

**Supplementary Fig. 25. Dose-response (a) and time-course (b) analyses of IMAG-mediated NO generation and metabolic effects (n=3).** Data are presented as mean ± SD.

**
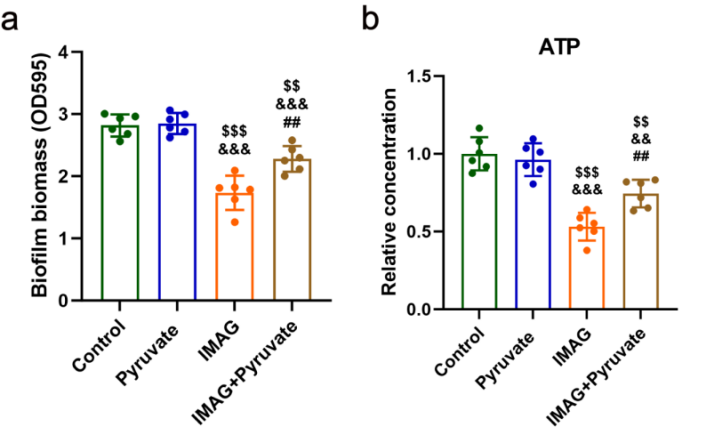
**

**Supplementary Fig. 26. Pyruvate supplementation partially rescued the metabolic suppression induced by IMAG (n=6). a,** Quantification of biofilm biomass. **b,** Intracellular ATP levels of *S. aureus* following treatment. Note: ^$$^*p* < 0.01 and ^$$$^*p* < 0.001 versus the control group; ^&&^*p* < 0.01 and ^&&&^*p* < 0.001 versus pyruvate group; ^##^*p* < 0.01 versus IMAG group. Data are presented as mean ± SD. Statistical significance was determined by one-way ANOVA.

**
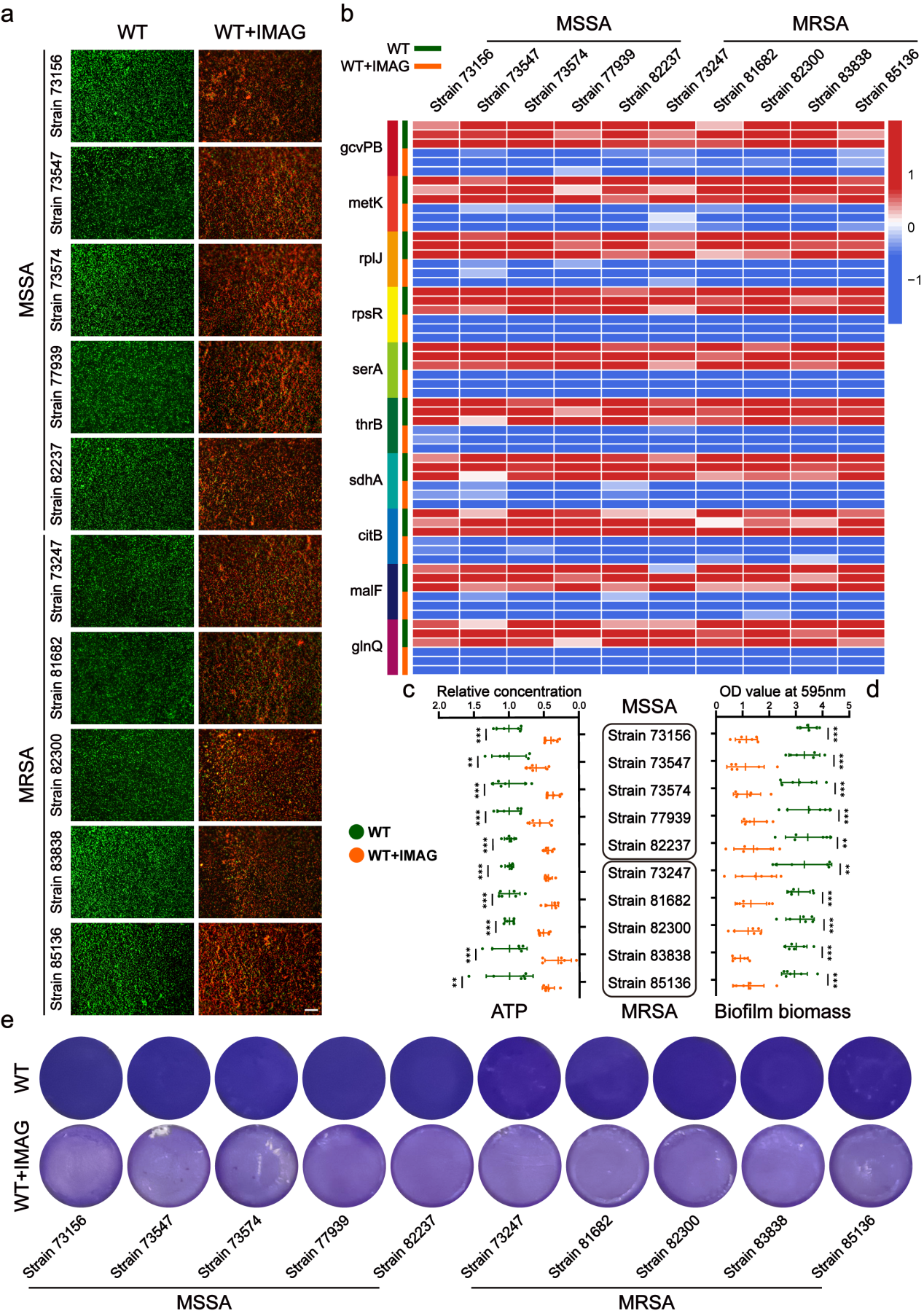
**

**Supplementary Fig. 27. IMAG exhibits excellent antibacterial efficacy against clinical *S. aureus* strains. a,** Representative live/dead staining images of clinical strains with or without IMAG treatment. Scale bar, 50 μm. **b,** Gene expression levels in IMAG-treated and untreated strains. **c,** ATP levels in clinical strains after different treatments (n=6). **d,** Biofilm biomass of IMAG-treated and untreated strains (n=6). **e,** Representative crystal violet staining images of biofilms from each clinical strain. Note: ^**^*p* < 0.01 and ^***^*p* < 0.001. Data are presented as mean ± SD. Statistical significance was determined by one-way ANOVA.


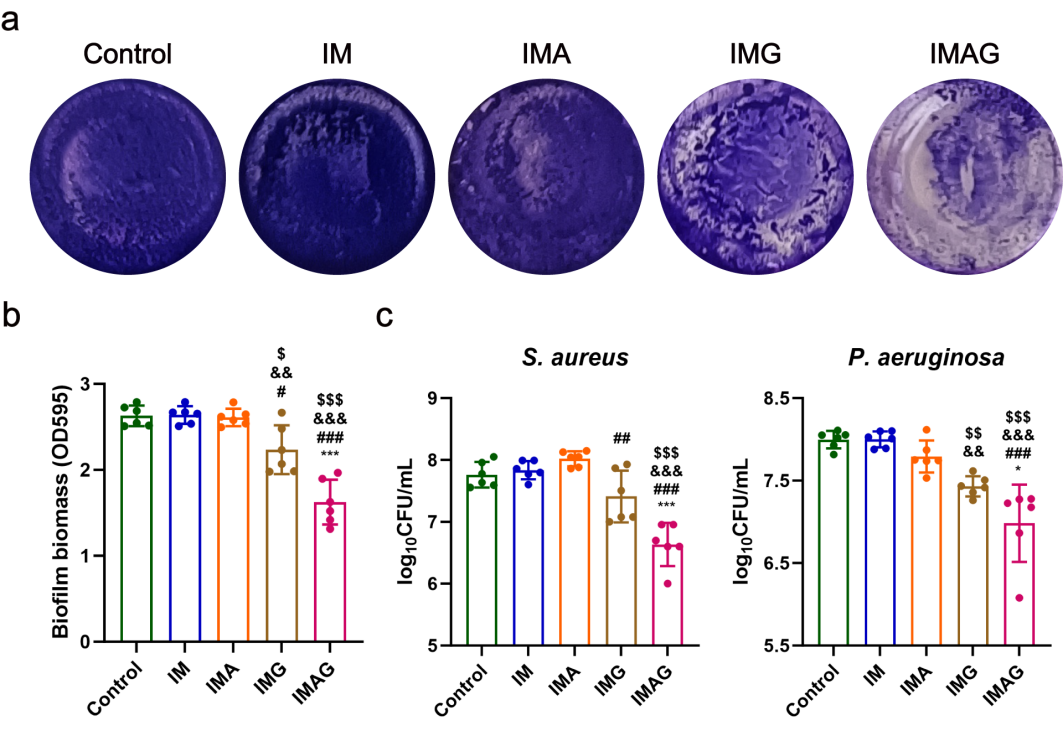


**Supplementary Fig. 28. Antibacterial effects of IMAG against polymicrobial biofilms formed by *S. aureus* and *P. aeruginosa*. a,** Representative crystal violet staining images of polymicrobial biofilms after different treatments. **b,** Quantification of polymicrobial biofilm biomass (n = 6). **c,** Quantification of bacterial burdens of *S. aureus* and *P. aeruginosa* in polymicrobial biofilms by CFU counting on selective agar plates (Mannitol Salt Agar for *S. aureus* and Cetrimide Agar for *P. aeruginosa*, n = 6). Note: ^$^*p* < 0.05, ^$$^*p* < 0.01 and ^$$$^*p* < 0.001 versus the control group; ^&&^*p* < 0.01 and ^&&&^*p* < 0.001 versus IM group; ^#^*p* < 0.05, ^##^*p* < 0.01 and ^###^*p* < 0.001 versus IMA group; ^*^*p* < 0.05 and ^***^*p* < 0.001 versus IMG group. Data are presented as mean ± SD. Statistical significance was determined by one-way ANOVA.


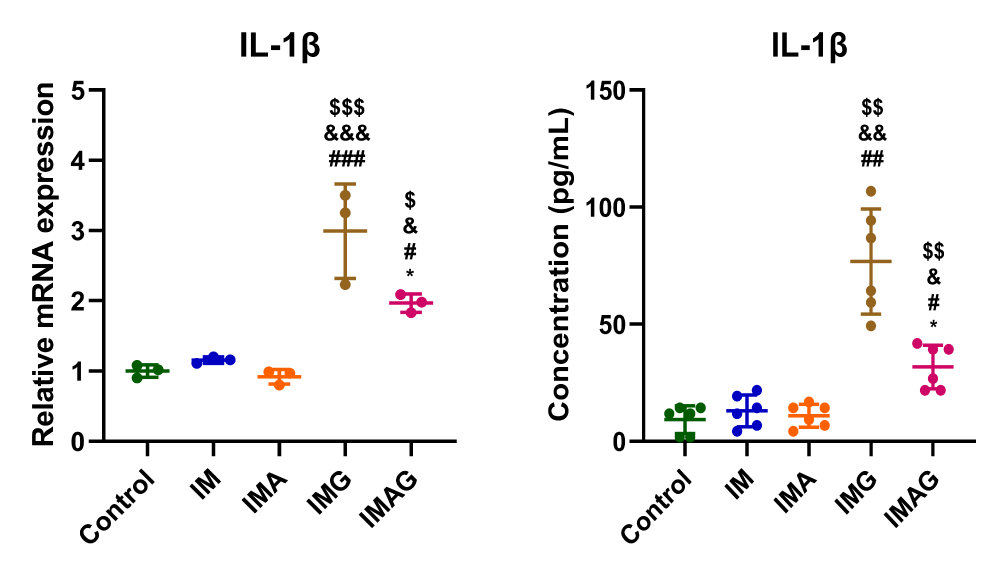


**Supplementary Fig. 29. The gene expression (n=3) and cytokine secretion level (n=6) of IL-1β in macrophages across different groups.** Note: ^$^*p* < 0.05, ^$$^*p* < 0.01 and ^$$$^*p* < 0.001 versus the control group; ^&^*p* < 0.05, ^&&^*p* < 0.01 and ^&&&^*p* < 0.001 versus IM group; ^#^*p* < 0.05, ^##^*p* < 0.01 and ^###^*p* < 0.001 versus IMA group; ^*^*p* < 0.05 versus IMG group. Data are presented as mean ± SD. Statistical significance was determined by one-way ANOVA.

**
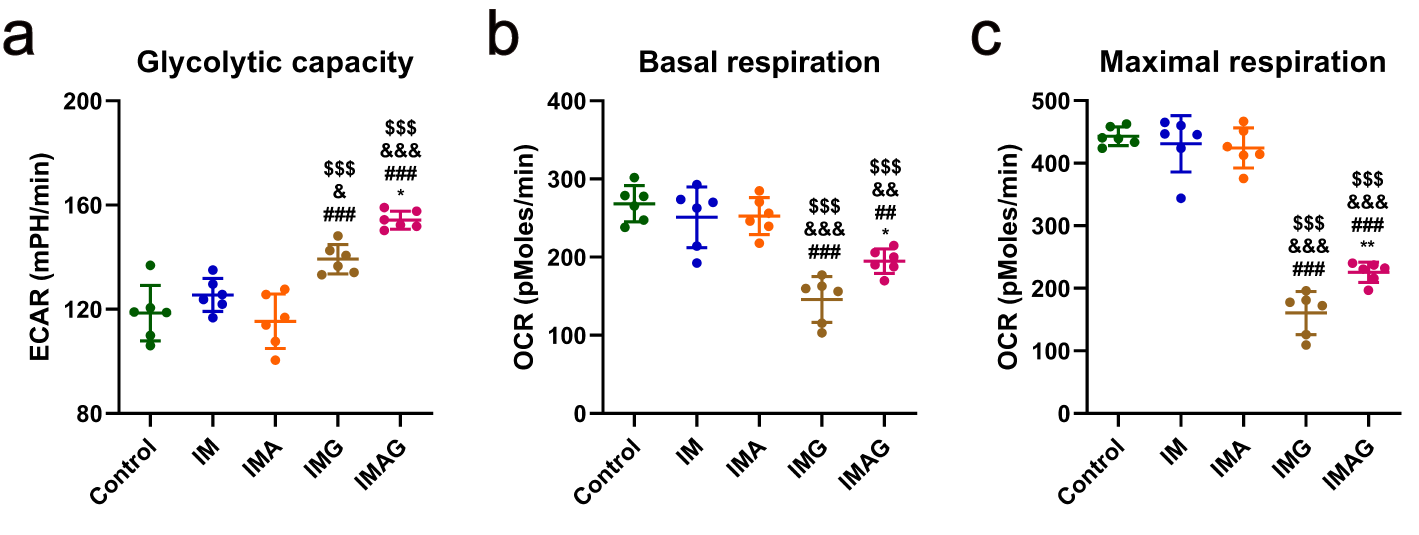
**

**Supplementary Fig. 30.** Assessment of glycolytic capacity (**a**), basal respiration (**b**), and maximal respiration (**c**) in macrophages from different treatment groups using the Seahorse XF Analyzer (n=6). Note: ^$$$^*p* < 0.001 versus the control group; ^&^*p* < 0.05, ^&&^*p* < 0.01 and ^&&&^*p* < 0.001 versus IM group; ^##^*p* < 0.01 and ^###^*p* < 0.001 versus IMA group; ^*^*p* < 0.05 and ^**^*p* < 0.01 versus IMG group. Data are /presented as mean ± SD. Statistical significance was determined by one-way ANOVA.

**
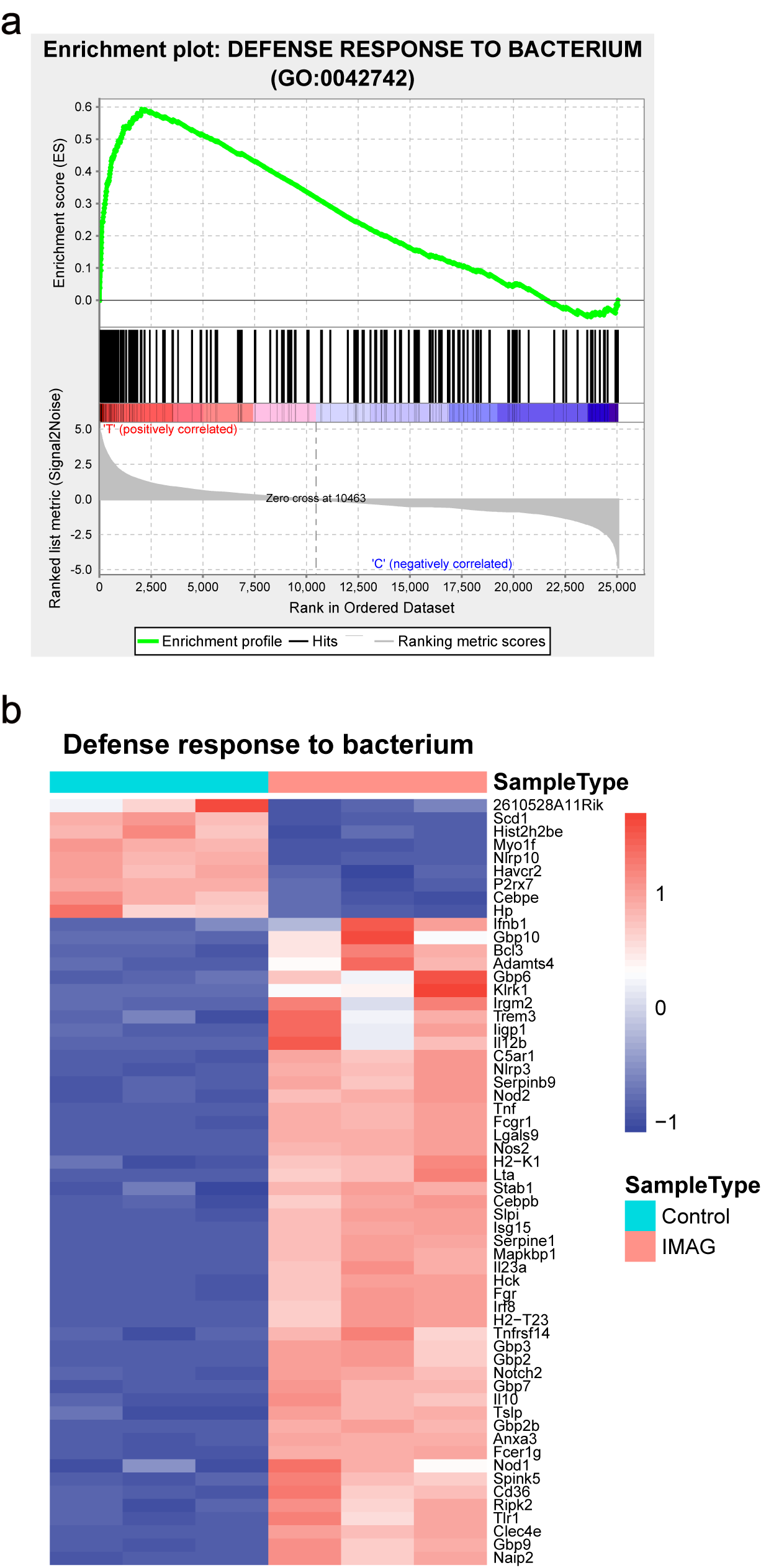
**

**Supplementary Fig. 31. GSEA and corresponding heatmap of DEGs in the defense response to bacterium pathway in IMAG-treated macrophages compared to controls.**


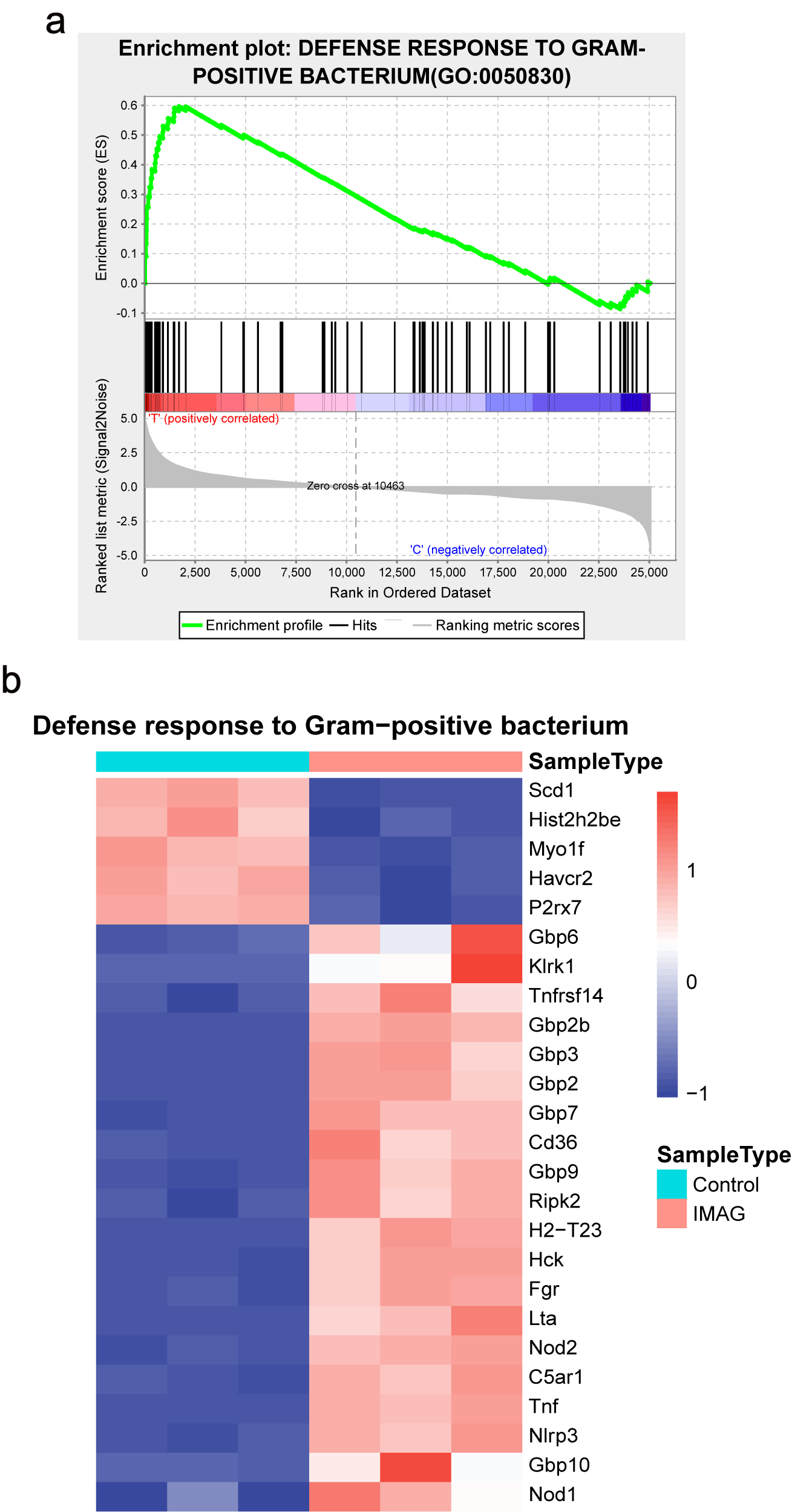


**Supplementary Fig. 32. GSEA and corresponding heatmap of DEGs in the defense response to Gram-positive bacterium pathway in IMAG-treated macrophages compared to controls.**

**
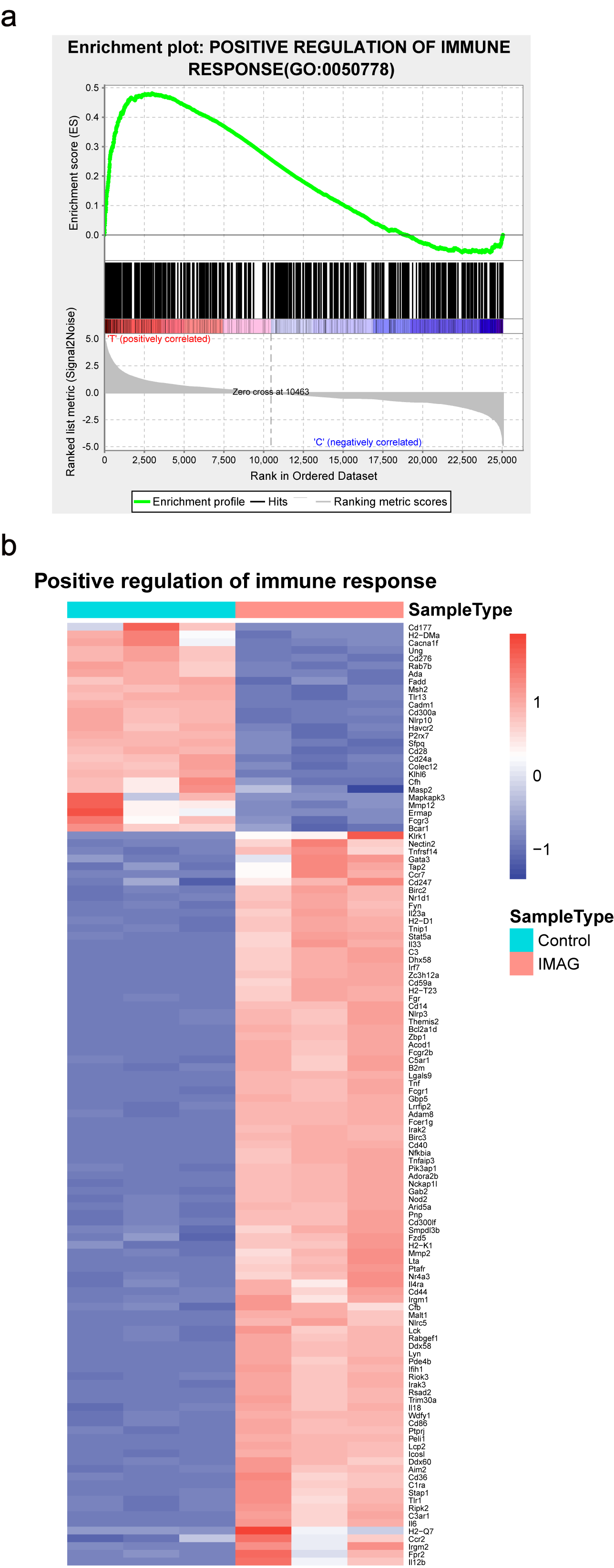
**

**Supplementary Fig. 33. GSEA and corresponding heatmap of DEGs in the positive regulation of immune response pathway in IMAG-treated macrophages compared to controls.**

**
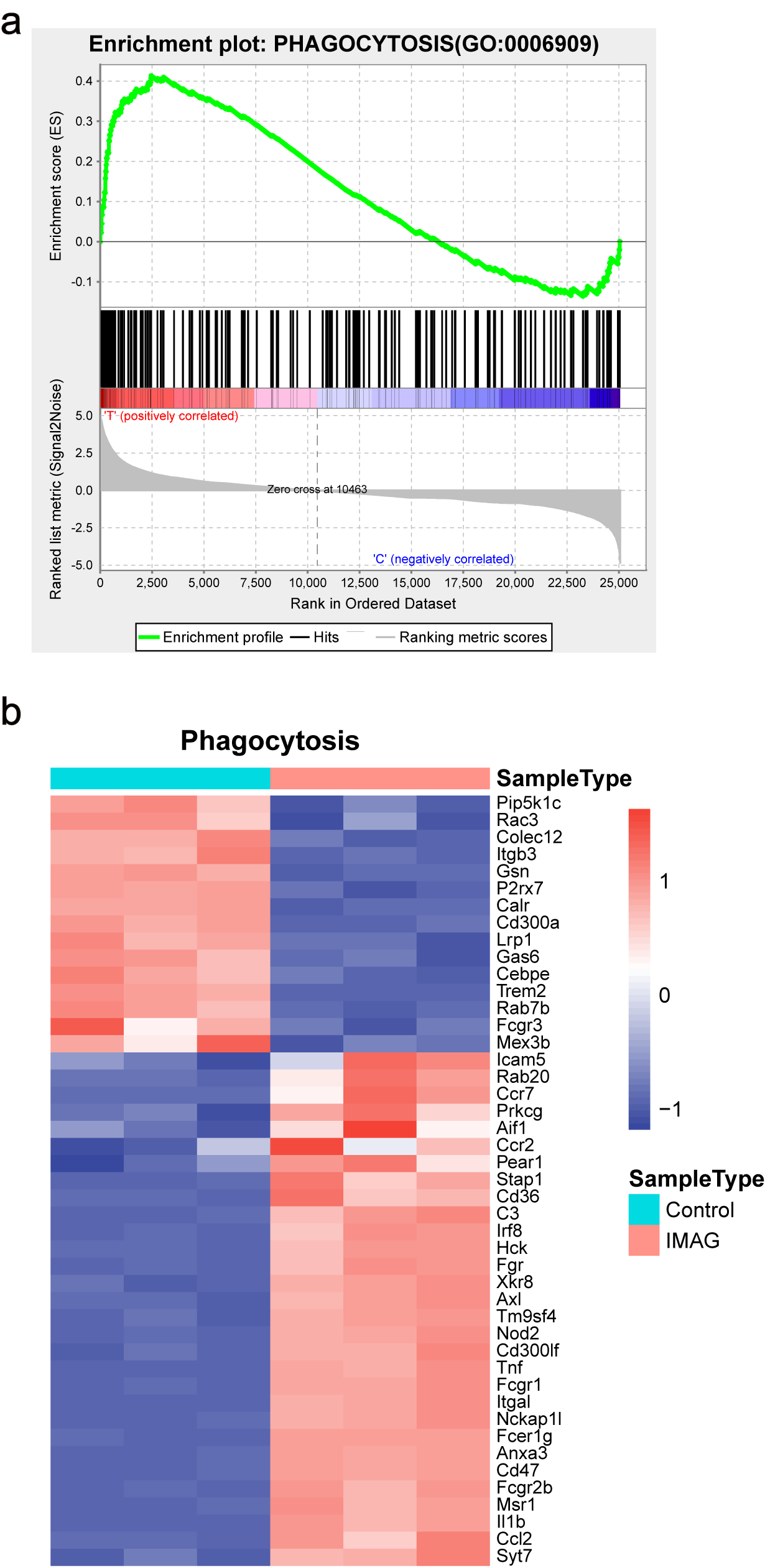
**

**Supplementary Fig. 34. GSEA and corresponding heatmap of DEGs in the phagocytosis pathway in IMAG-treated macrophages compared to controls.**

**
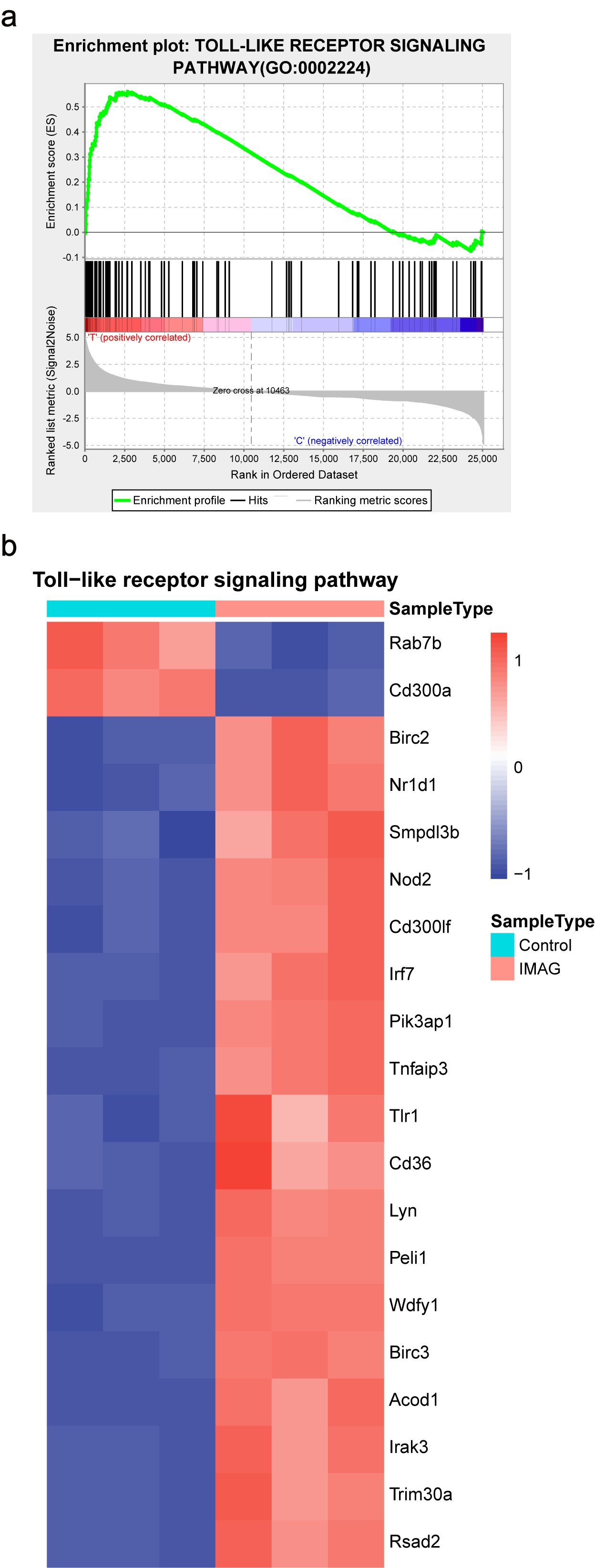
**

**Supplementary Fig. 35. GSEA and corresponding heatmap of DEGs in the toll-like receptor signaling pathway in IMAG-treated macrophages compared to controls.**

**
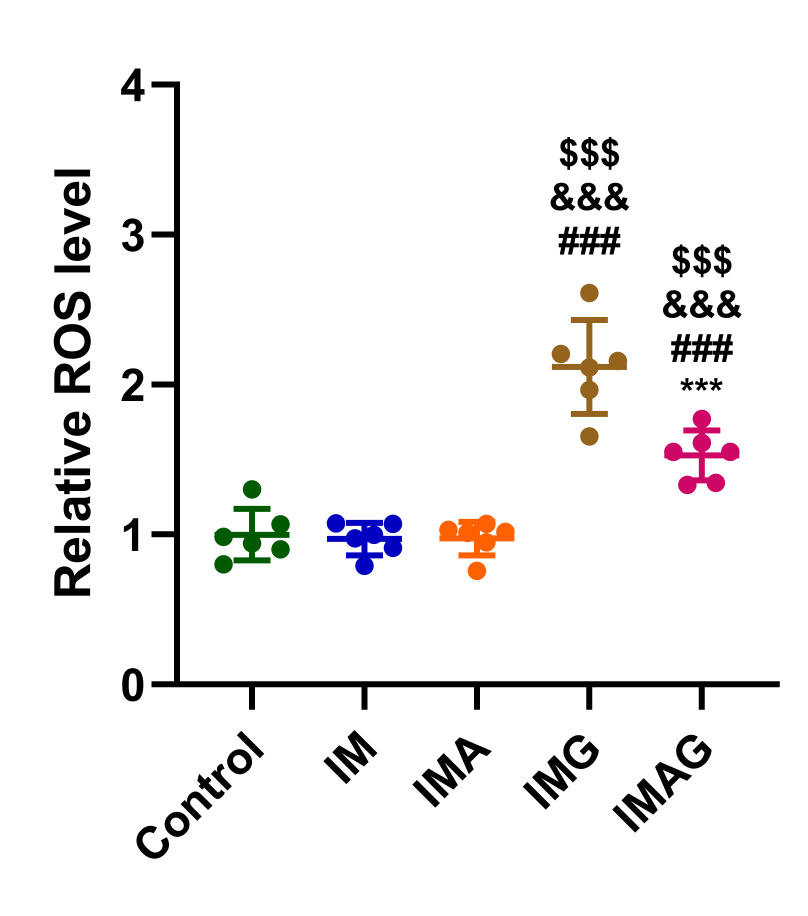
**

**Supplementary Fig. 36. Quantification of intracellular ROS relative fluorescence intensity in macrophages across different groups (n=6).** Note: ^$$$^*p* < 0.001 versus the control group; ^&&&^*p* < 0.001 versus IM group; ^###^*p* < 0.001 versus IMA group; ^***^*p* < 0.001 versus IMG group. Data are presented as mean ± SD. Statistical significance was determined by one-way ANOVA.

**
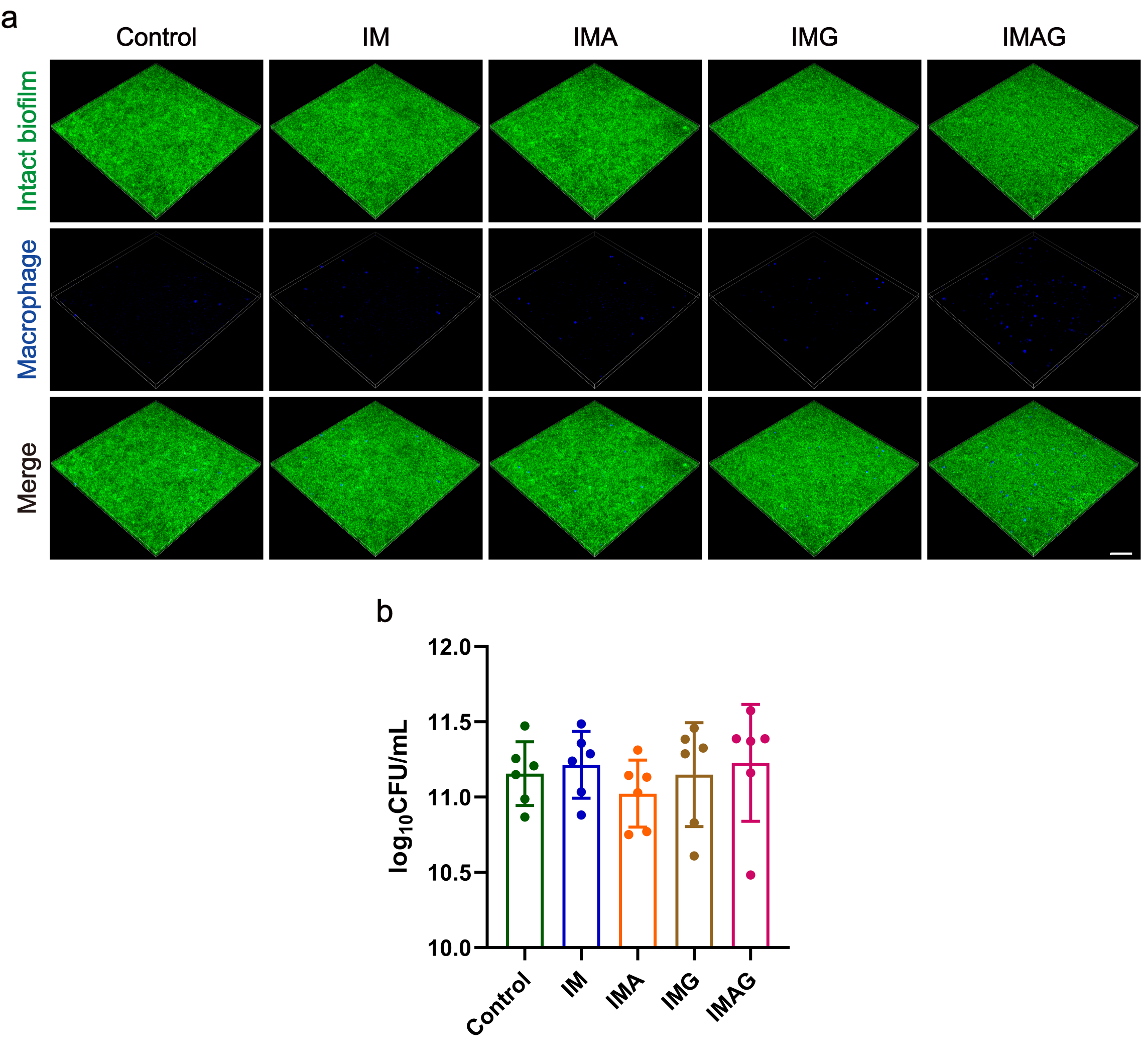
**

**Supplementary Fig. 37. a,** Representative CLSM images showing macrophage adhesion and invasion to untreated biofilms in different groups, with *S. aureus* biofilm shown in green and macrophages in blue. Scale bar, 200 μm. **b,** Bacterial load in untreated biofilms after co-culture with different groups of macrophages (n=6). Data are presented as mean ± SD. Statistical significance was determined by one-way ANOVA.

**
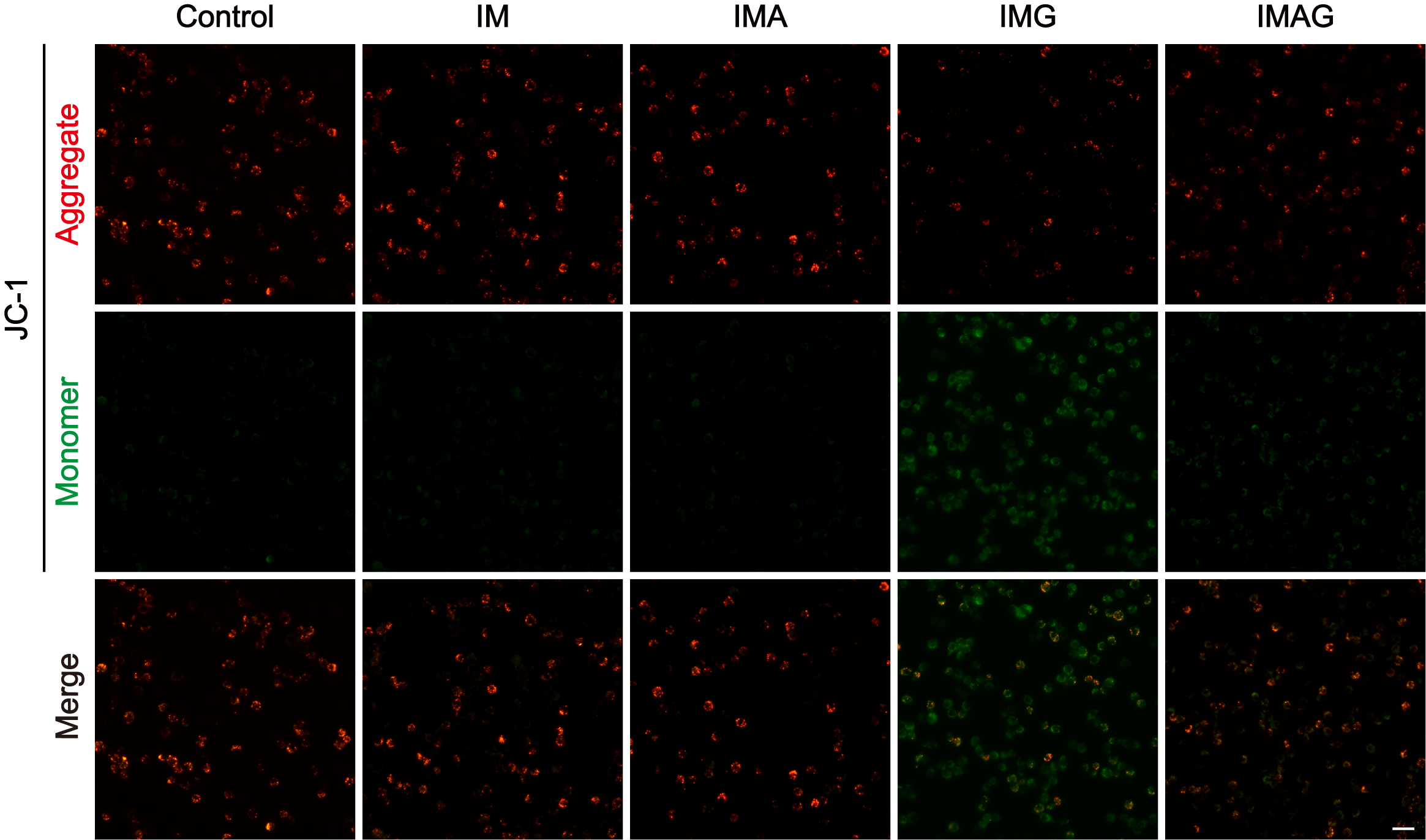
**

**Supplementary Fig. 38. Representative JC-1 staining images of mitochondria in macrophages from each treatment group.** Red indicates JC-1 aggregates, and green indicates JC-1 monomers. Scale bar, 25 μm.


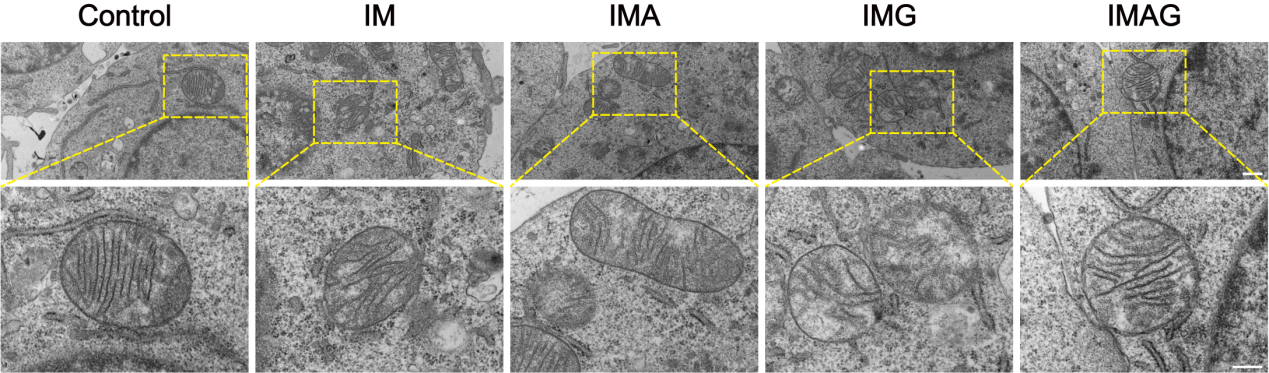


**Supplementary Fig. 39. Representative TEM images of mitochondrial morphology in macrophages from each treatment group.** Scale bars, 500 nm and 250 nm.

**
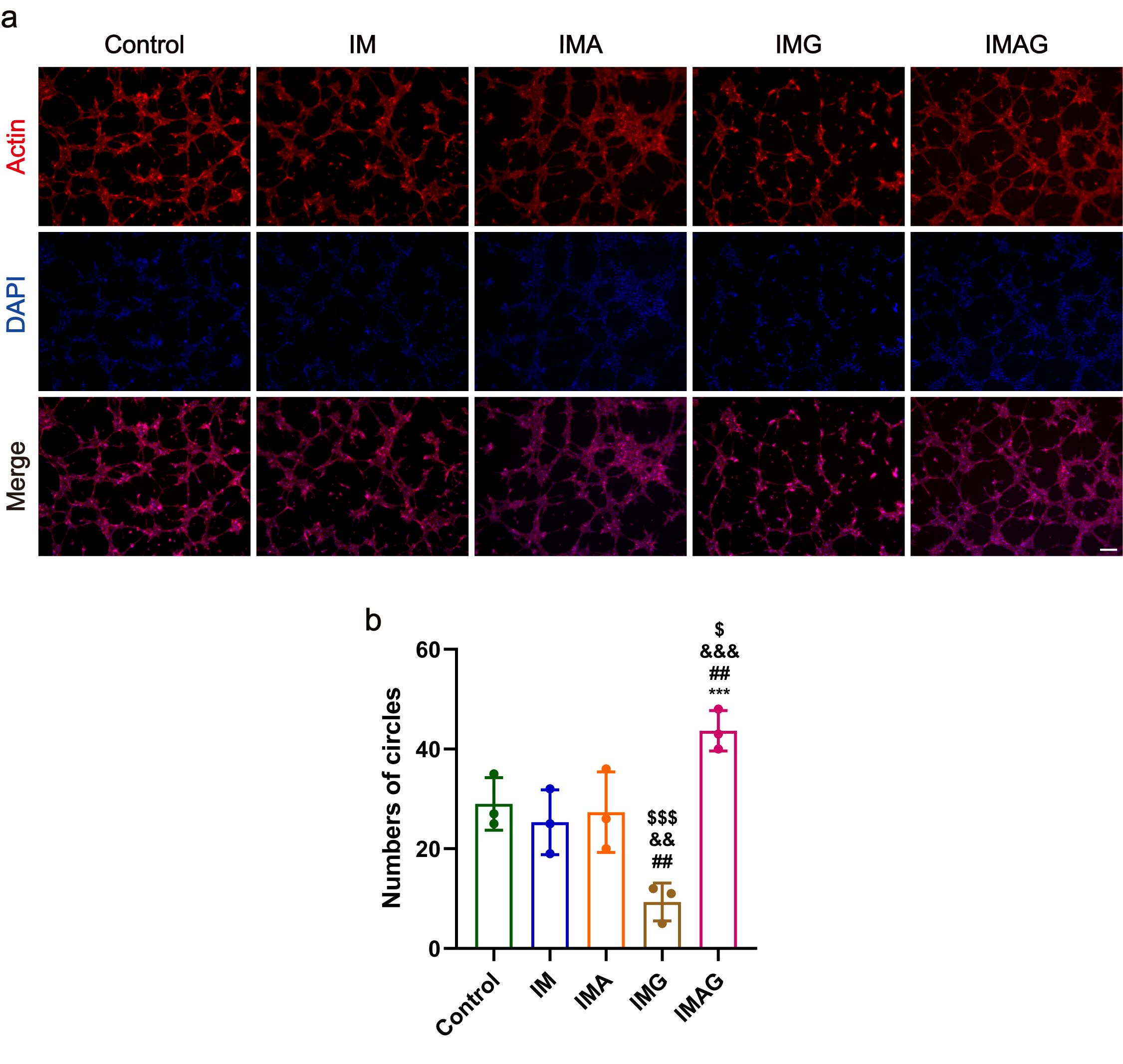
**

**Supplementary Fig. 40. Tube Formation Assay. a,** Representative images of angiogenesis formed by HUVECs in different groups, with the cytoskeleton in red and nuclei in blue. Scale bar, 200 μm. **b,** Number of vascular circles formed in each group (n=3). Note: ^$^*p* < 0.05 and ^$$$^*p* < 0.001 versus the control group; ^&&^*p* < 0.01 and ^&&&^*p* < 0.001 versus IM group; ^##^*p* < 0.01 versus IMA group; ^***^*p* < 0.001 versus IMG group. Data are presented as mean ± SD. Statistical significance was determined by one-way ANOVA.


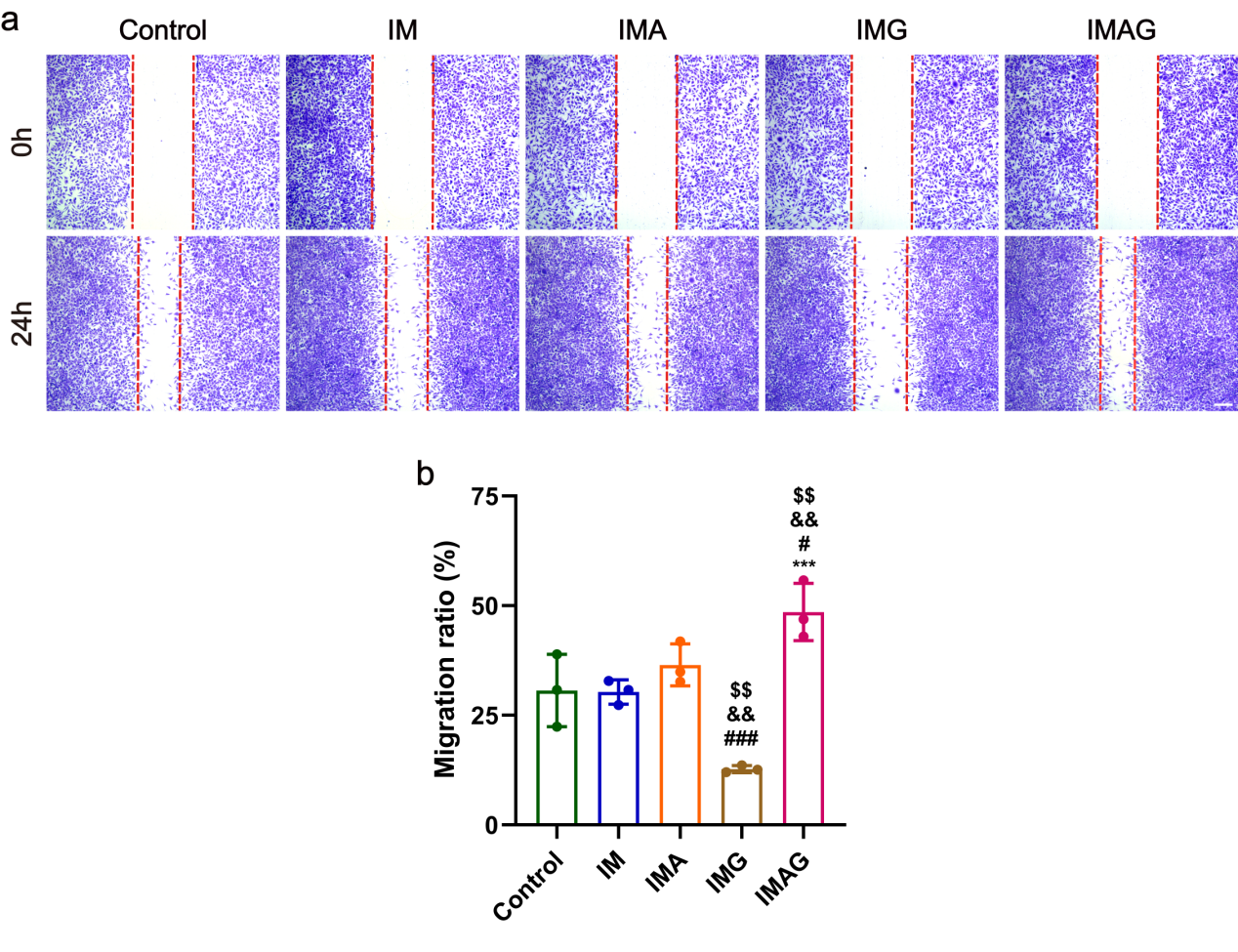


**Supplementary Fig. 41. Cell Migration Assay. a,** Representative images of fibroblast migration at 0 hours and 24 hours post-treatment. Scale bar, 200 μm. **b,** Migration rates in each group (n=3). Note: ^$$^*p* < 0.01 versus the control group; ^&&^*p* < 0.01 versus IM group; ^#^*p* < 0.05 and ^###^*p* < 0.001 versus IMA group; ^***^*p* < 0.001 versus IMG group. Data are presented as mean ± SD. Statistical significance was determined by one-way ANOVA.


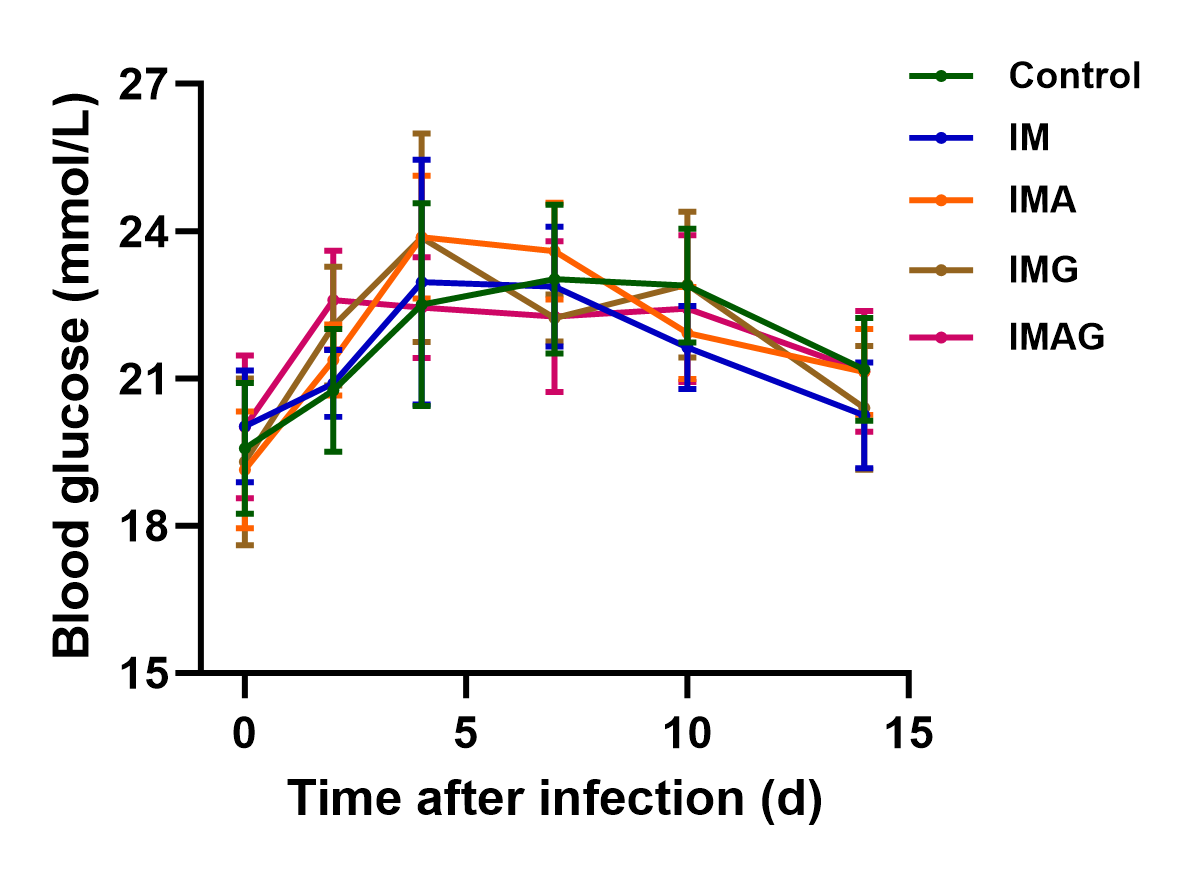


**Supplementary Fig. 42. Blood glucose monitoring in the implant-associated infection model (n=6).** Data are presented as mean ± SD.

**
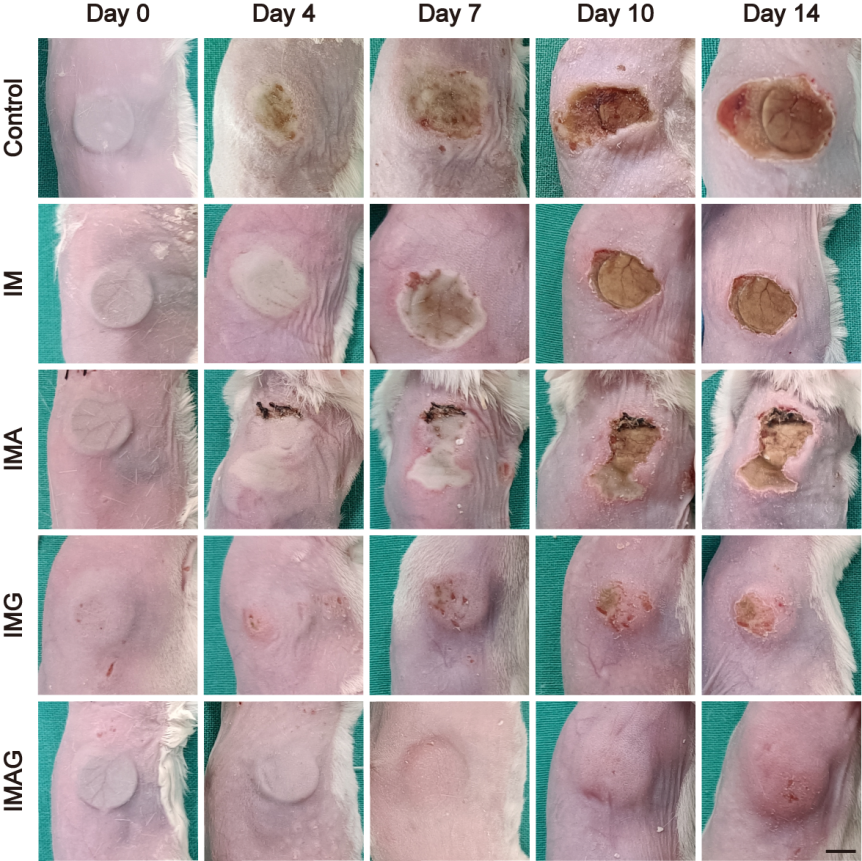
**

**Supplementary Fig. 43. Representative images of infected peri-implant tissues from different treatment groups at various time points.** Scale bar, 500 μm.

**
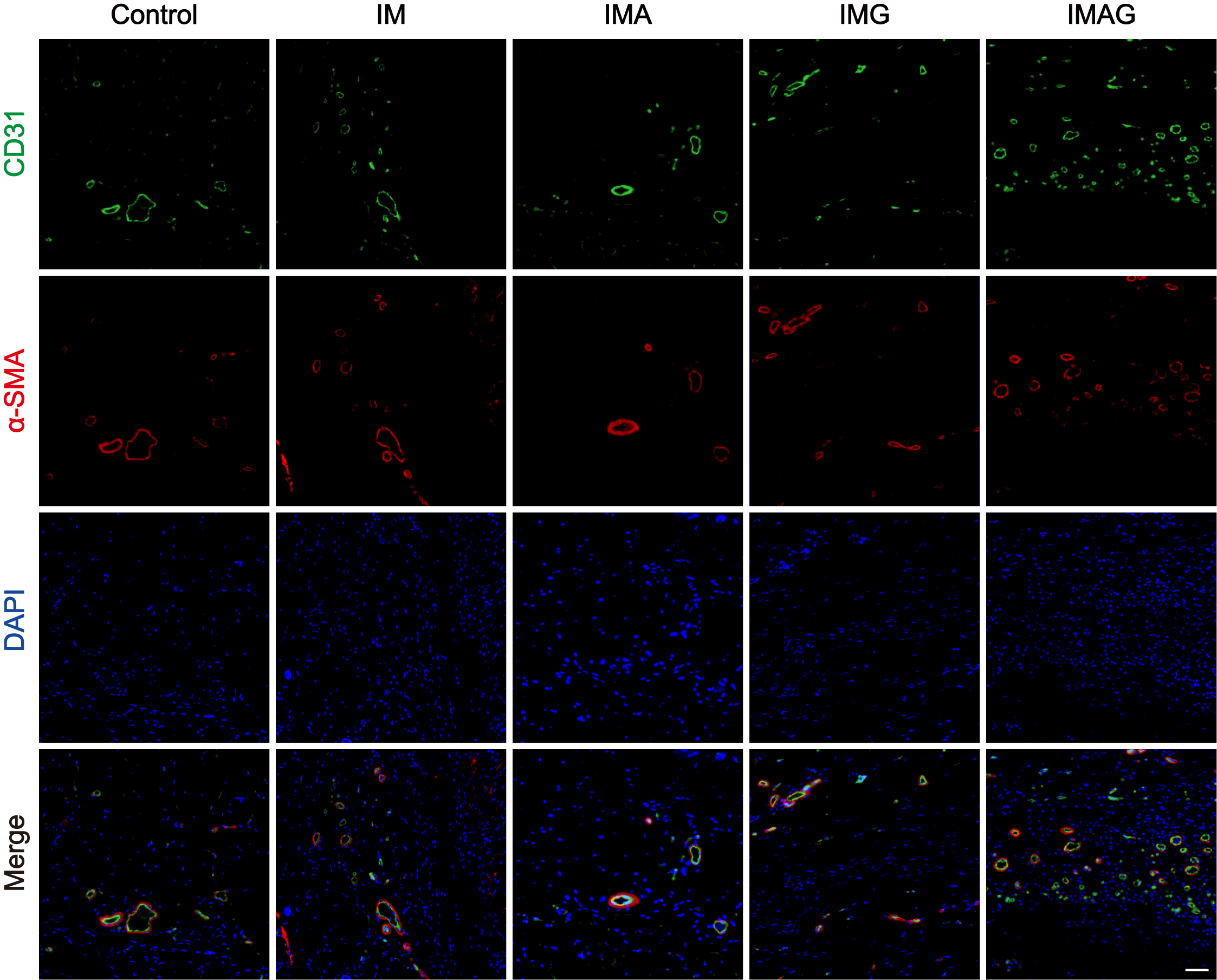
**

**Supplementary Fig. 44. Angiogenesis on day 14 post-infection.** Representative immunofluorescence images of angiogenesis markers CD31 (green) and α-SMA (red) at the infection sites on day 14. Scale bar, 50 μm.

**
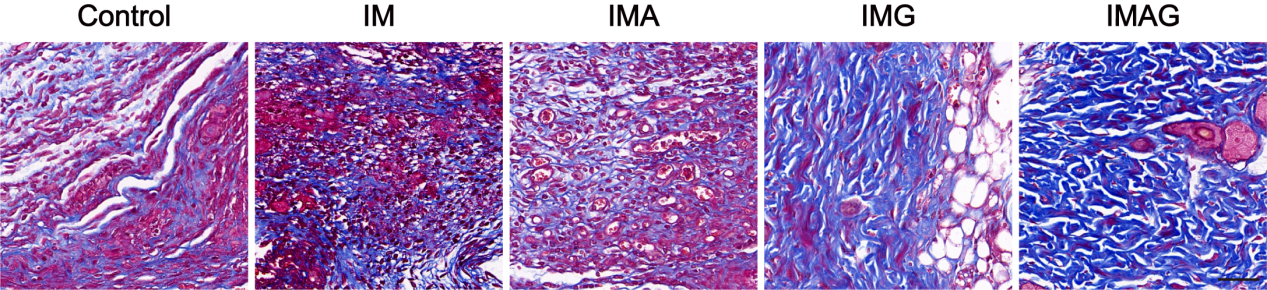
**

**Supplementary Fig. 45. Collagen deposition on day 14 post-infection.** Representative masson staining images of peri-implant tissues on day 14. Scale bar, 50 μm.


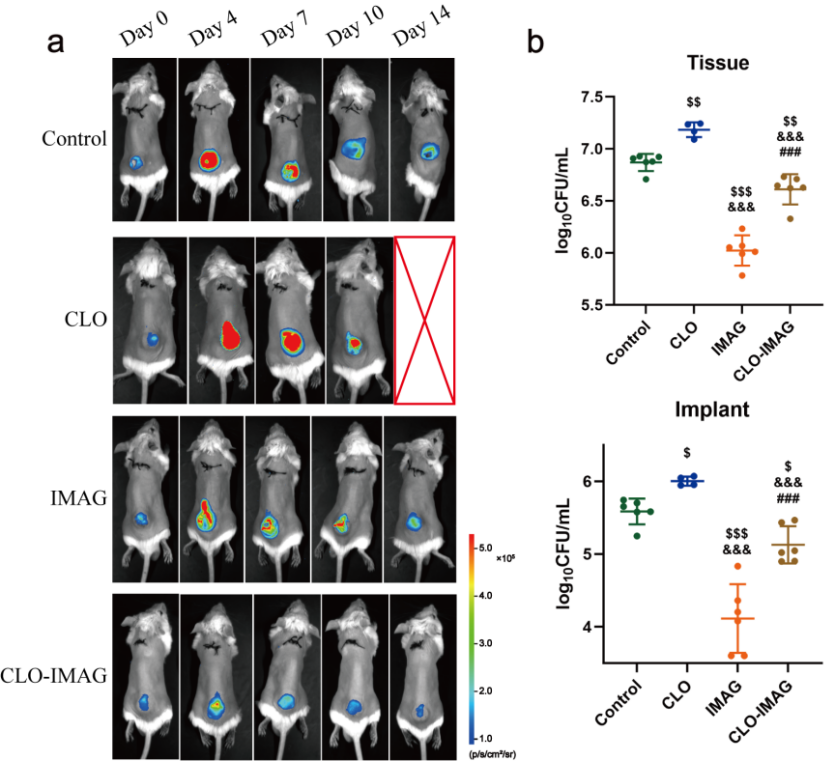


**Supplementary Fig. 46. Macrophage depletion in a diabetic mouse model of implant-associated infection. a,** Representative bioluminescence images after infection. **b,** Bacterial CFU counts in periprosthetic tissues and implants on day 14 (Control, IMAG, CLO-IMAG, n=6; CLO, n=4). Note: ^$^*p* < 0.05, ^$$^*p* < 0.01 and ^$$$^*p* < 0.001 versus the control group; ^&&&^*p* < 0.001 versus CLO group; ^###^*p* < 0.001 versus IMAG group. Data are presented as mean ± SD. Statistical significance was determined by one-way ANOVA.


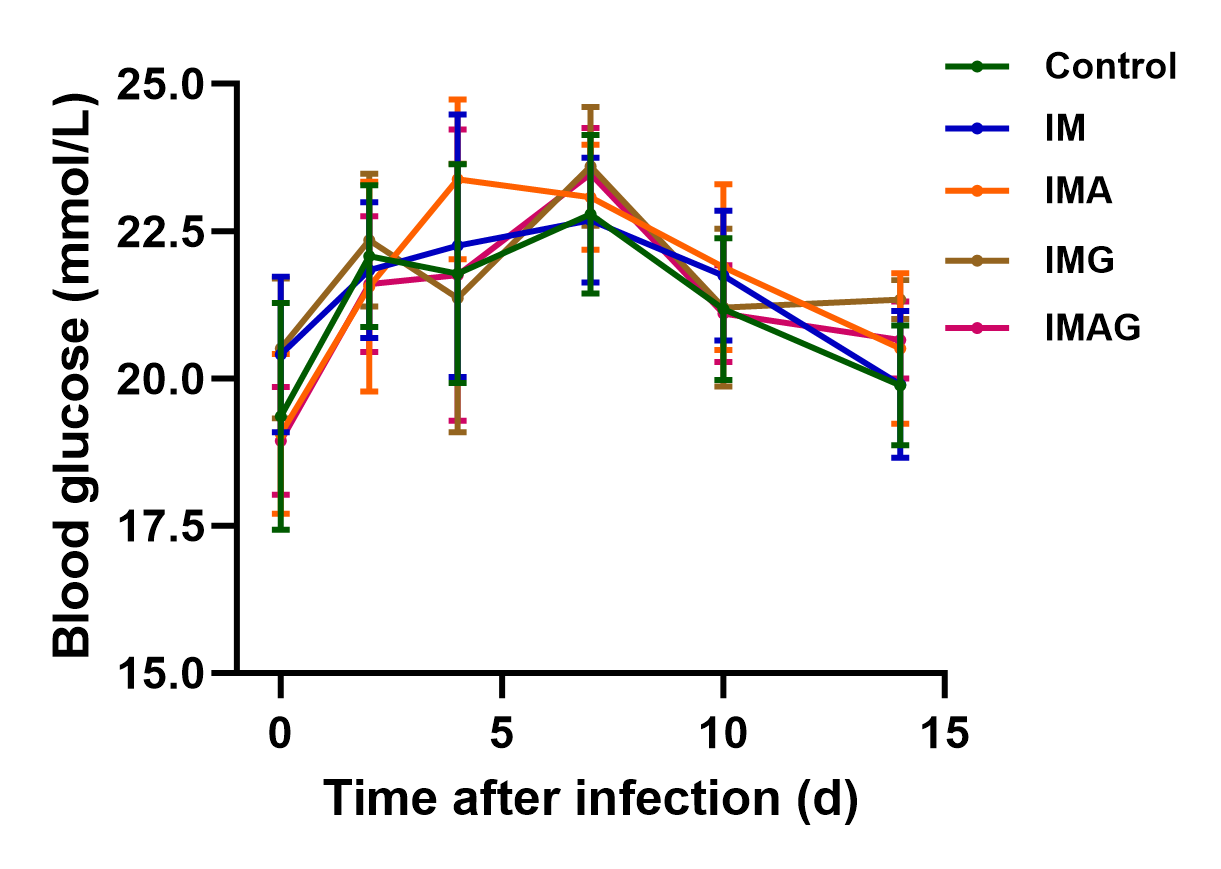


**Supplementary Fig. 47. Blood glucose monitoring in the wound infection model (n=6).** Data are presented as mean ± SD.


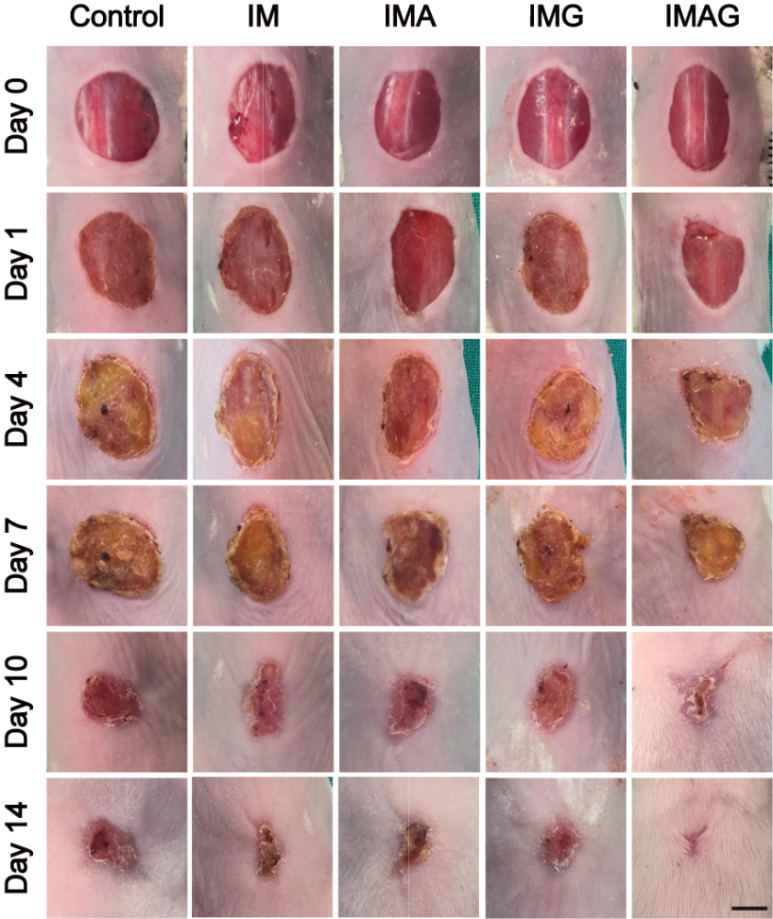


**Supplementary Fig. 48 Representative images of wounds from different treatment groups on days 0, 1, 4, 7, 10, and 14 post-infection.** Scale bar, 500 μm.

**
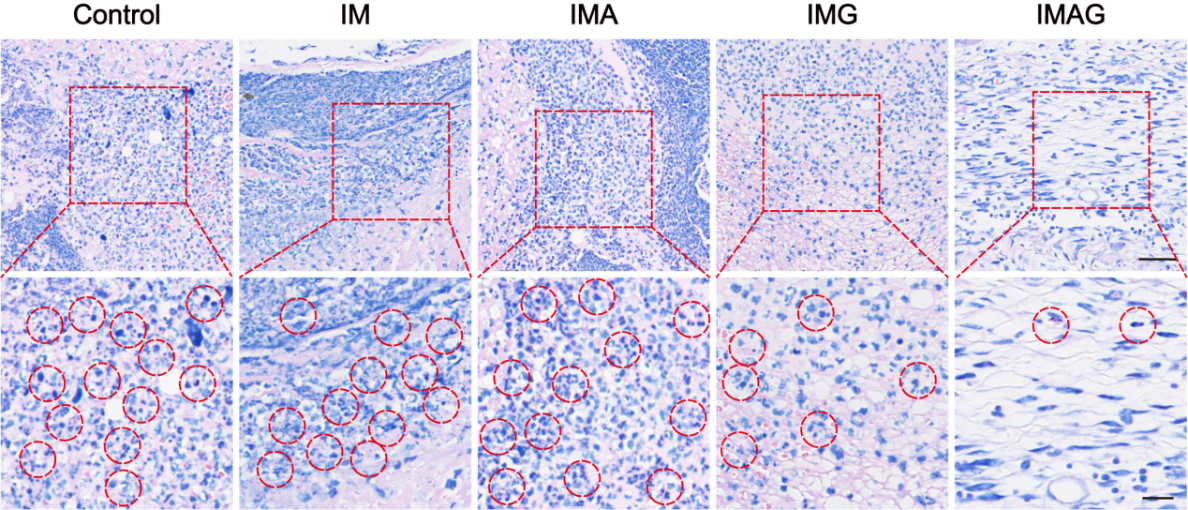
**

**Supplementary Fig. 49. Representative images of Giemsa-stained infected skin on day 14.** Scale bars, 50 μm and 25 μm.


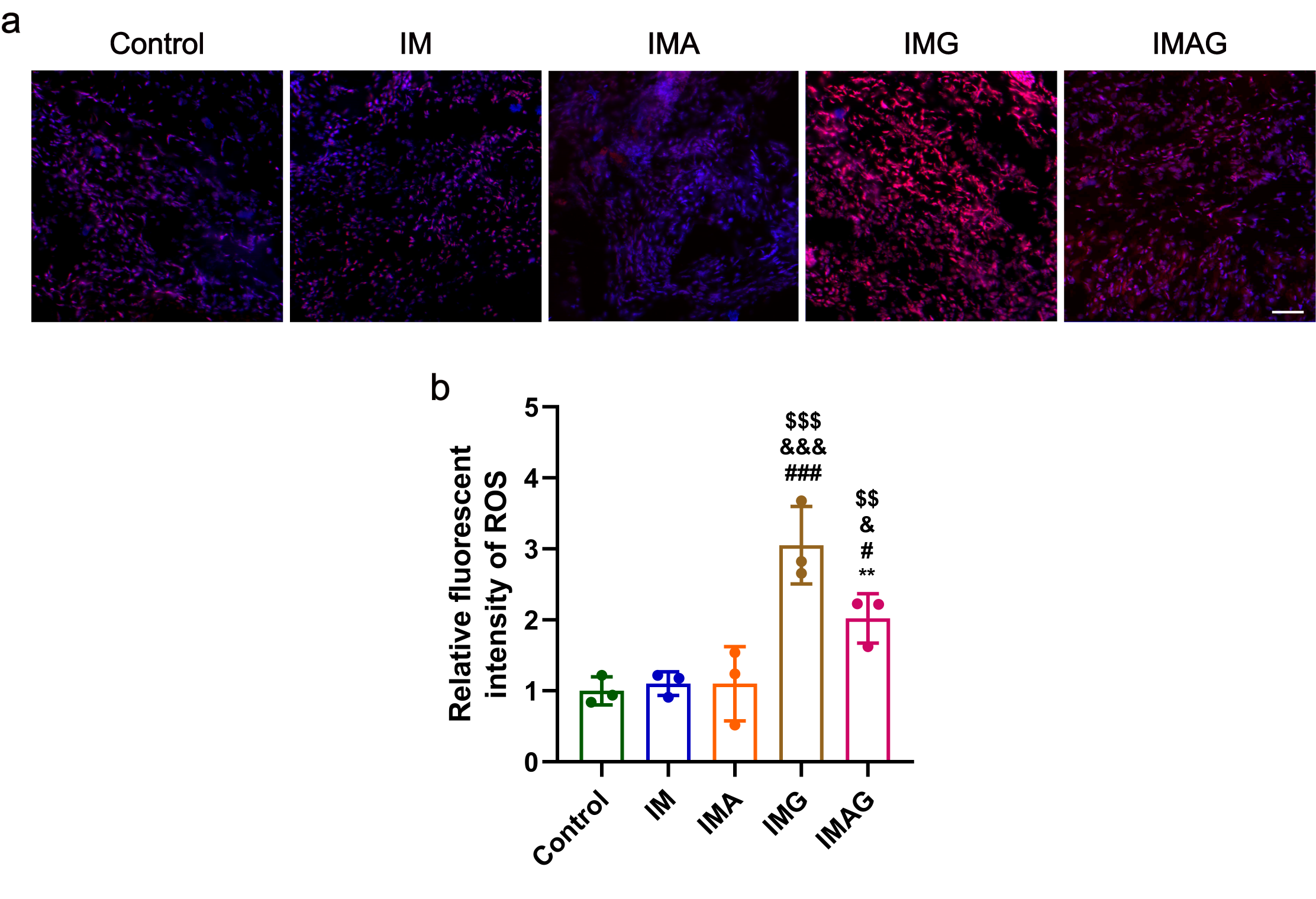


**Supplementary Fig. 50. Representative ROS staining images and relative fluorescence intensity of infected skin on day 4 post-infection (n=3).** Scale bar, 50 μm. Note: ^$$^*p* < 0.01 and ^$$$^*p* < 0.001 versus the control group; ^&^*p* < 0.05, ^&&^*p* < 0.01 and ^&&&^*p* < 0.001 versus IM group; ^#^*p* < 0.05, ^##^*p* < 0.01 and ^###^*p* < 0.001 versus IMA group; ^**^*p* < 0.01 and ^***^*p* < 0.001 versus IMG group. Data are presented as mean ± SD. Statistical significance was determined by one-way ANOVA.


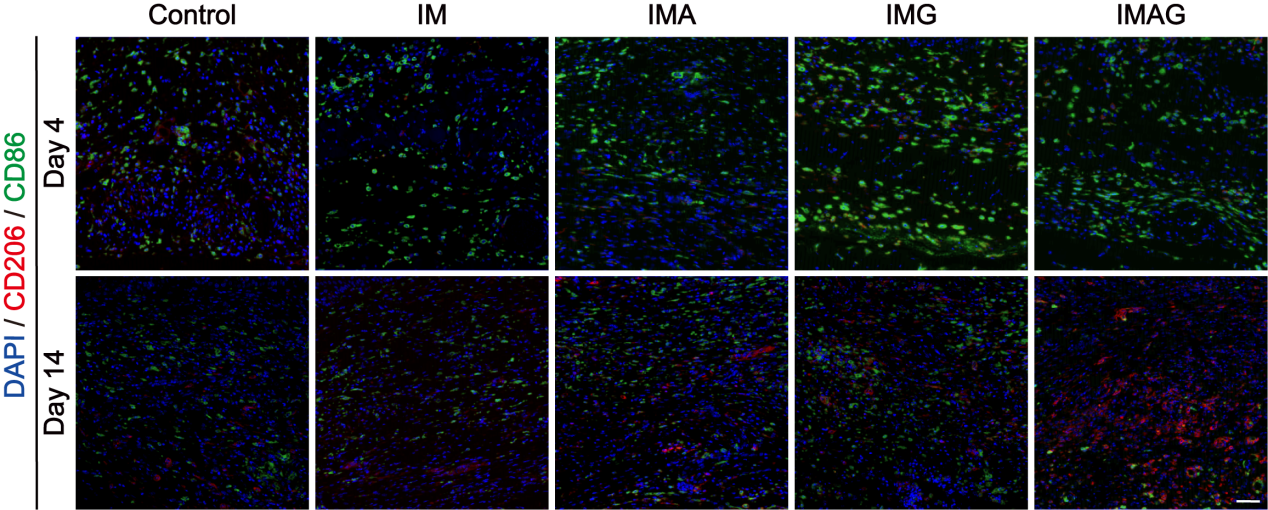


**Supplementary Fig. 51. Macrophage phenotype changes in infected wounds.** Representative immunofluorescence images of CD86 (green) and CD206 (red) in infected wounds on days 4 and 14 post-surgery. Scale bar, 50 μm.


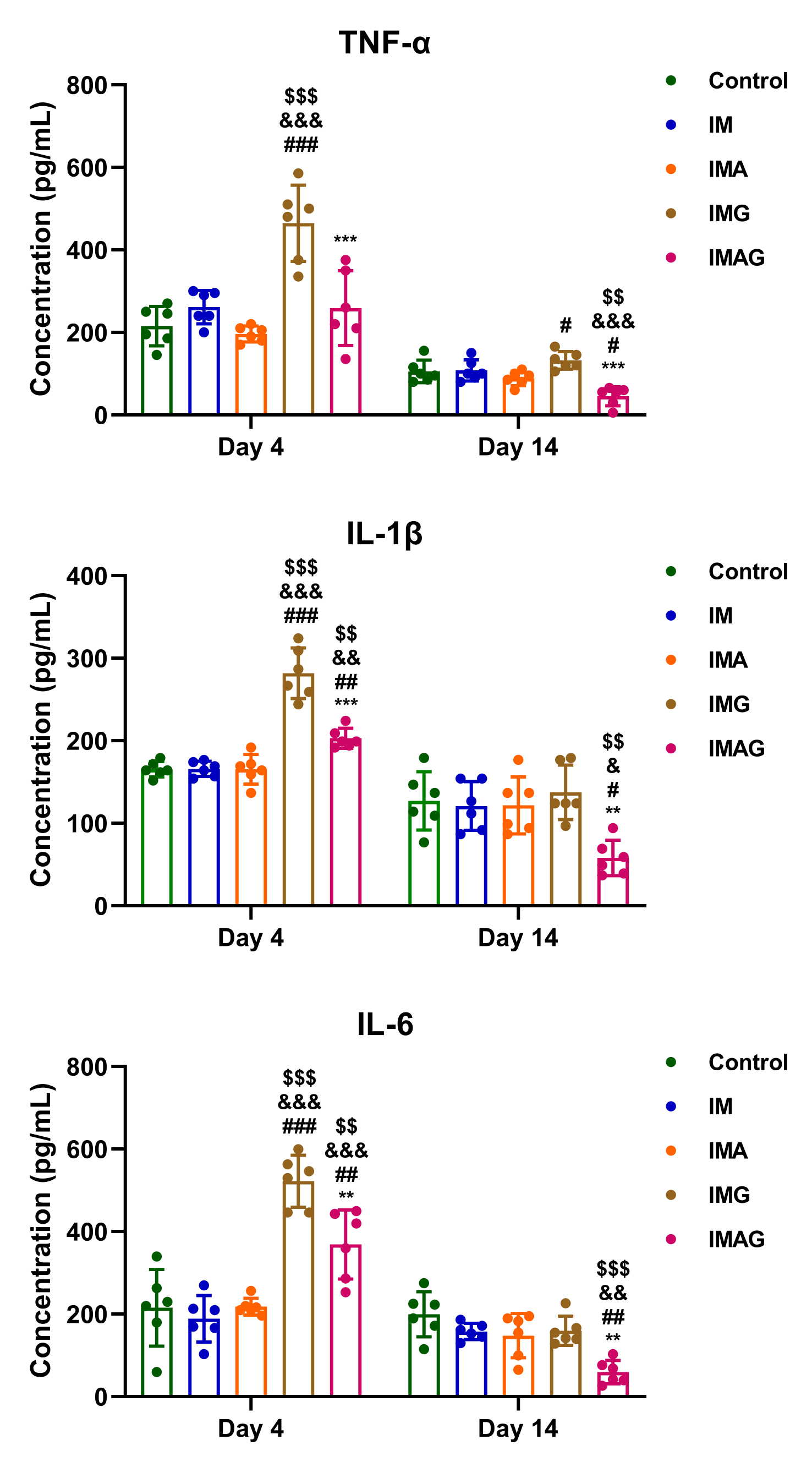


**Supplementary Fig. 52. ELISA results of TNF-α, IL-1β, and IL-6 in infected skin tissues from different groups on days 4 and 14 post-infection (n=6).** Note: ^$$^*p* < 0.01 and ^$$$^*p* < 0.001 versus the control group; ^&^*p* < 0.05 and ^&&&^*p* < 0.001 versus IM group; ^#^*p* < 0.05 and ^###^*p* < 0.001 versus IMA group; ^**^*p* < 0.01 versus IMG group. Data are presented as mean ± SD. Statistical significance was determined by one-way ANOVA.


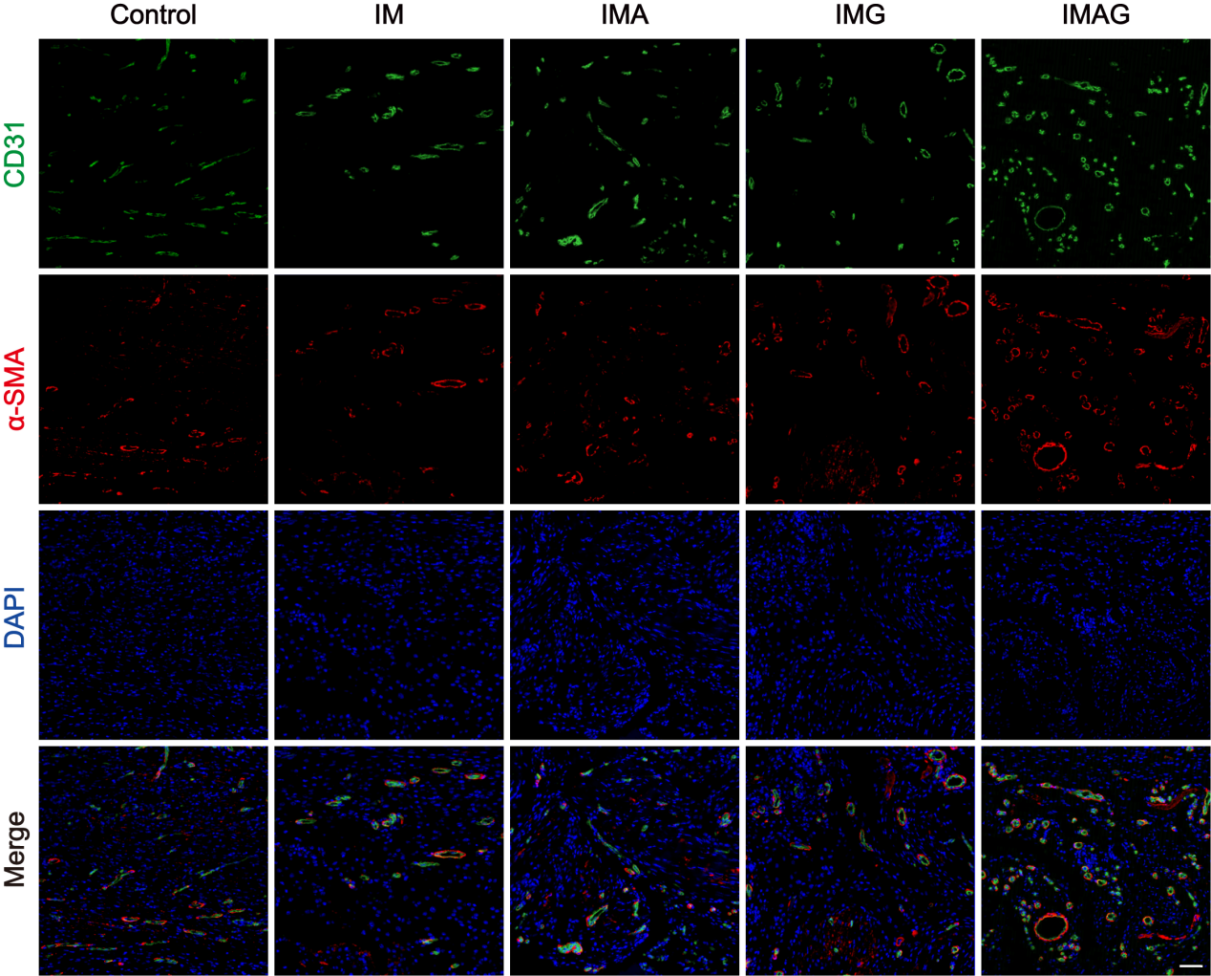


**Supplementary Fig. 53. Angiogenesis in each group on day 14.** Representative immunofluorescence images of angiogenesis markers CD31 (green) and α-SMA (red) in infected wounds on day 14 post-surgery. Scale bar, 50 μm.

**
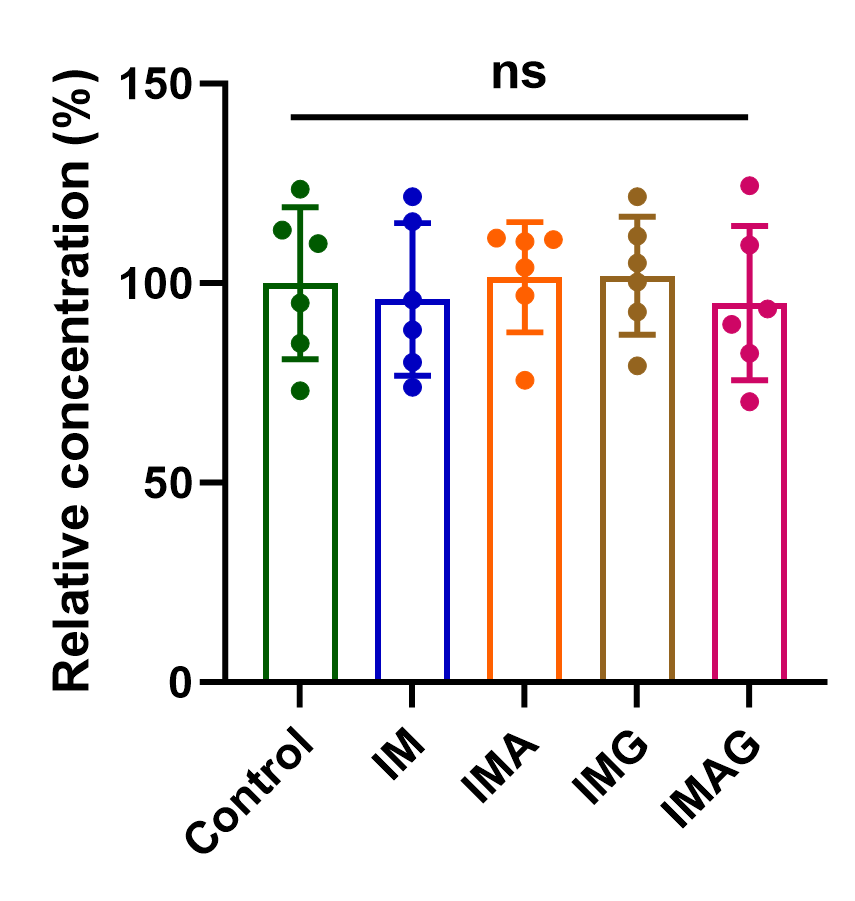
**

**Supplementary Fig. 54. Relative concentration of 3-NT in mice after different treatments (n = 6).** Data are presented as mean ± SD. Statistical significance was determined by one-way ANOVA.

**
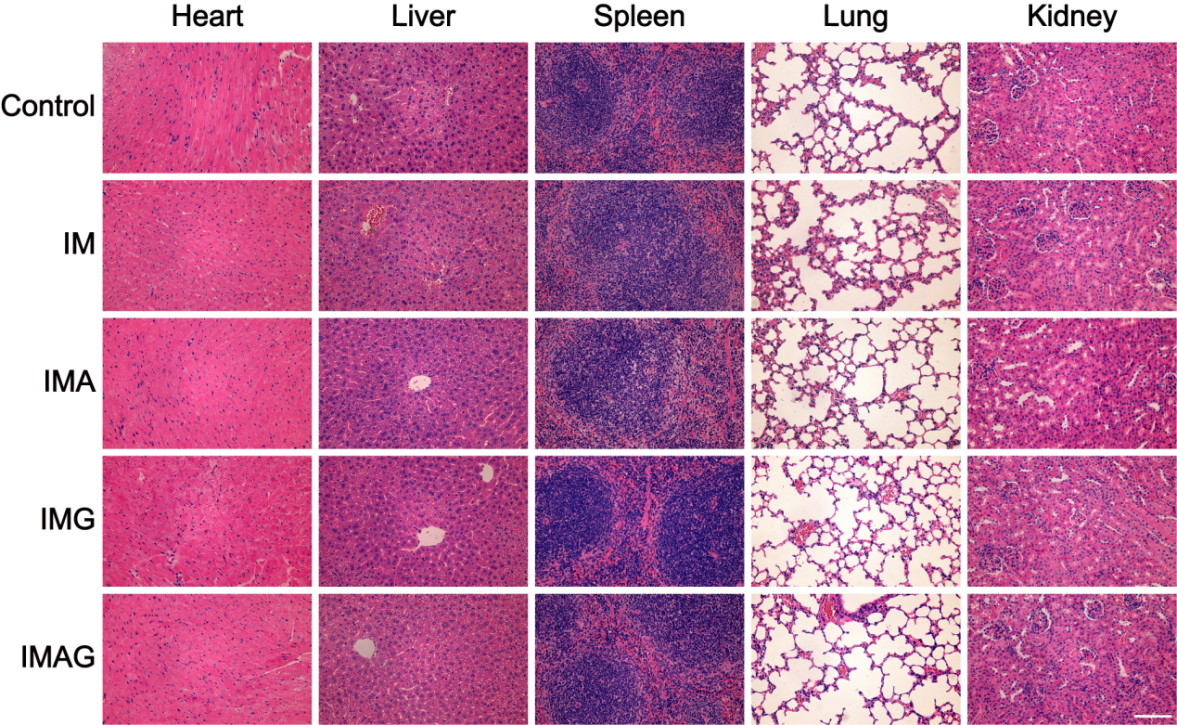
**

**Supplementary Fig. 55. Representative H&E staining images of major organs from different groups of mice.** Scale bar, 100 μm.


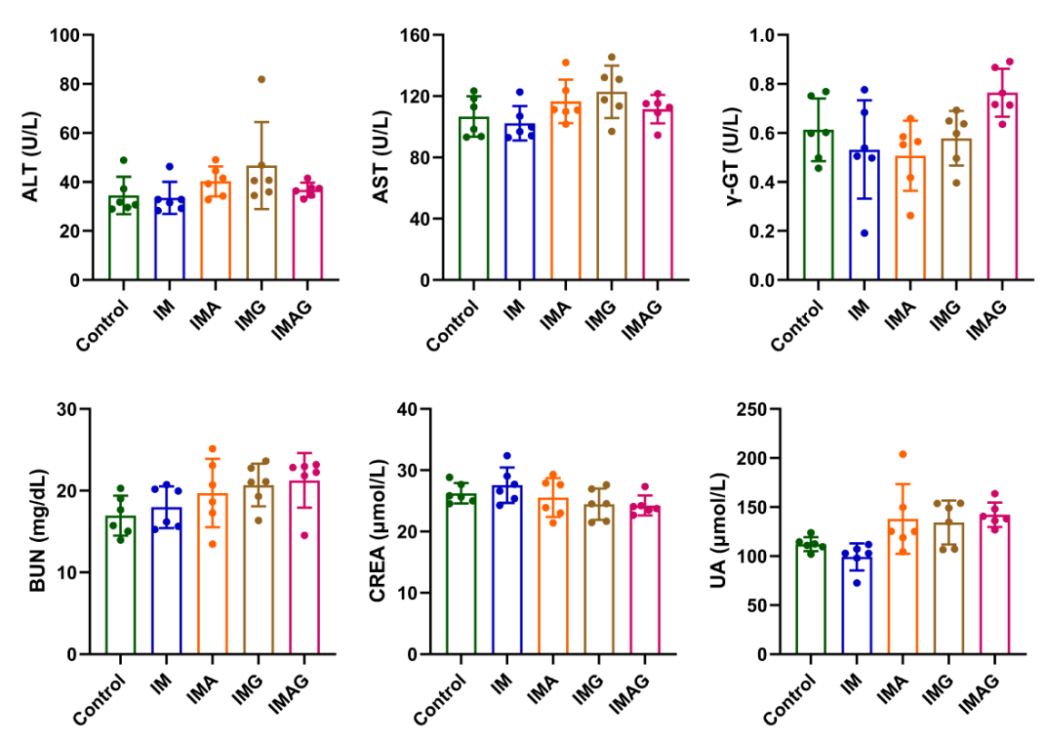


**Supplementary Fig. 56. Serum biochemistry analyses across groups (n=6).** Data are presented as mean ± SD. Statistical significance was determined by one-way ANOVA.


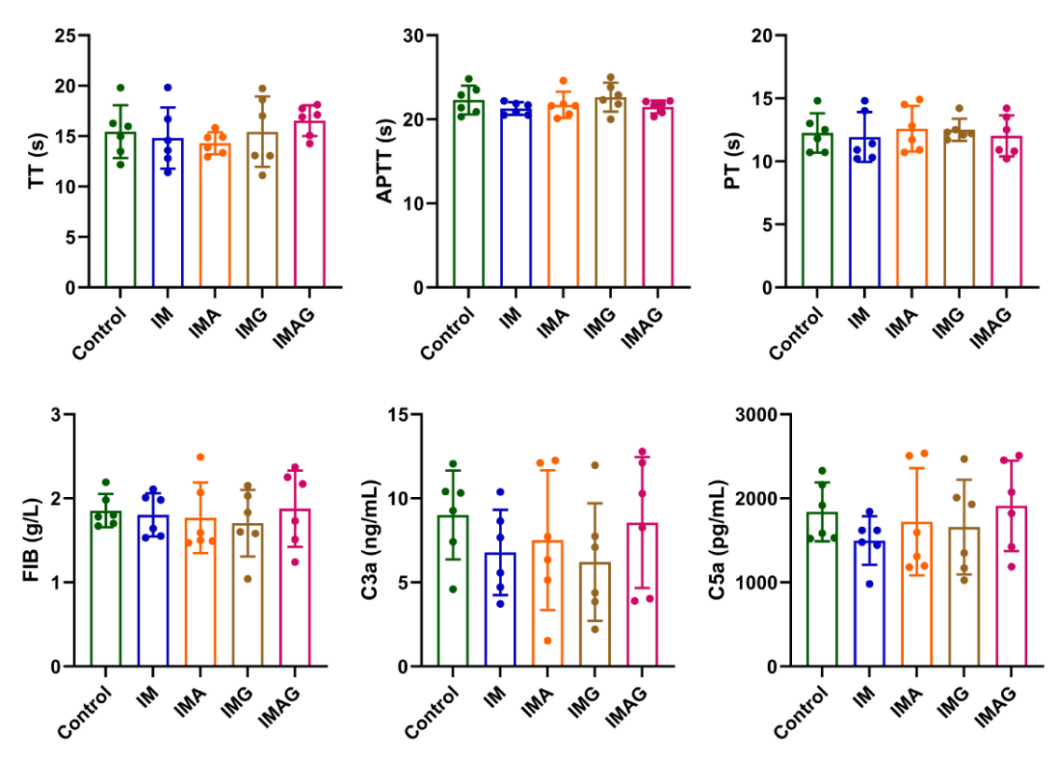


**Supplementary Fig. 57. Coagulation parameters and serum complement (C3a, C5a) levels in mice after nanoparticle treatment (n = 6).** Data are presented as mean ± SD. Statistical significance was determined by one-way ANOVA.

**Supplementary Table 1. Fold reduction of vancomycin MBEC against *S. aureus* biofilms under IMAG co-treatment.**

| Strain | Type | Vancomycin (μg/mL) | | Fold reduction |
| --- | --- | --- | --- | --- |
|  |  | MBEC (alone) | MBEC  (with IMAG) |  |
| ST8 | MRSA | 2048 | 1024 | 2 |
| 73156 | MSSA | 1024 | 256 | 4 |
| 73574 | MSSA | 512 | 128 | 4 |
| 82237 | MSSA | 1024 | 512 | 2 |
| 77939 | MSSA | 2048 | 512 | 4 |
| 73547 | MSSA | 2048 | 1024 | 2 |
| 81682 | MRSA | 1024 | 768 | 1.33 |
| 73247 | MRSA | 4096 | 2048 | 2 |
| 82300 | MRSA | 2048 | 1536 | 1.33 |
| 85136 | MRSA | 4096 | 2048 | 2 |
| 83838 | MRSA | 4096 | 1536 | 2.67 |

**Note: Fold reduction = MBEC_van, alone_ /MBEC_van, with IMAG_**

**Supplementary Table 2. Primers used in RT-PCR assays in this study.**

| Primer | Primers Sequence (5’−3’) |
| --- | --- |
| gyrB-F | GGTGGCGACTTTGATCTAGC |
| gyrB-R | TTATACAACGGTGGCTGTGC |
| metK-F | GTTCTGCAACACCAATCGCA |
| metK-R | GGCGGATGCTTCAGTGGTAA |
| serA-F | CGCCGGTGTTGGTGTAGATA |
| serA-R | AAGTGATTGGTGTGCTTGCG |
| thrB-F | TGGCAAGAGGGTTAGGTTCG |
| thrB-R | TCGGCGCAACATTATCAGGA |
| gcvPB-F | GAAGCTGAGGCTGGATTCGT |
| gcvPB-R | GCGCACATGGTGAATGGAC |
| sdhA-F | TGACCCACGCAAAGTACCAA |
| sdhA-R | CACCTAAGCGGTTACCACCA |
| citB-F | CATCTCTCCAGCAGGTGCAA |
| citB-R | AGCAAACGTACCTCGAACCA |
| rplJ-F | TCAGGCGTTATGGAAGGCAA |
| rplJ-R | AAGCGAAGTTGCGTACAGGA |
| rpsR-F | ATGGCAGGTGGACCAAGAAG |
| rpsR-R | AGCTGAAGTACCAGTTACACGA |
| malF-F | TGACACAACTGCACCCATGT |
| malF-R | ACCAGTGTCAGGGCAGAATG |
| glnQ-F | AAACCAATGATGCGTGTGCC |
| glnQ-R | CGTGCGCTATGCCAAGAATC |
| β-actin-F | GGCTGTATTCCCCTCCATCG |
| β-actin-R | CCAGTTGGTAACAATGCCATGT |
| IL-1β-F | GAAATGCCACCTTTTGACAGTG |
| IL-1β-R | TGGATGCTCTCATCAGGACAG |
| IL-6-F | TAGTCCTTCCTACCCCAATTTC |
| IL-6-R | TTGGTCCTTAGCCACTCCTTC |
| TNF-α-F | CTGAACTTCGGGGTGATCGG |
| TNF-α-R | GGCTTGTCACTCGAATTTTGAGA |
